# Proteogenomic analysis of psoriasis reveals discordant and concordant changes in mRNA and protein abundance

**DOI:** 10.1186/s13073-015-0208-5

**Published:** 2015-08-04

**Authors:** William R. Swindell, Henriette A. Remmer, Mrinal K. Sarkar, Xianying Xing, Drew H. Barnes, Liza Wolterink, John J. Voorhees, Rajan P. Nair, Andrew Johnston, James T. Elder, Johann E. Gudjonsson

**Affiliations:** Department of Dermatology, University of Michigan School of Medicine, Ann Arbor, MI 48109-2200 USA; Department of Biological Chemistry, University of Michigan School of Medicine, Ann Arbor, MI 48109-2200 USA

## Abstract

**Background:**

Psoriasis is a chronic disease characterized by the development of scaly red skin lesions and possible co-morbid conditions. The psoriasis lesional skin transcriptome has been extensively investigated, but mRNA levels do not necessarily reflect protein abundance. The purpose of this study was therefore to compare differential expression patterns of mRNA and protein in psoriasis lesions.

**Methods:**

Lesional (PP) and uninvolved (PN) skin samples from 14 patients were analyzed using high-throughput complementary DNA sequencing (RNA-seq) and liquid chromatography-tandem mass spectrometry (LC-MS/MS).

**Results:**

We identified 4122 differentially expressed genes (DEGs) along with 748 differentially expressed proteins (DEPs). Global shifts in mRNA were modestly correlated with changes in protein abundance (*r* = 0.40). We identified similar numbers of increased and decreased DEGs, but 4-fold more increased than decreased DEPs. Ribosomal subunit and translation proteins were elevated within lesions, without a corresponding shift in mRNA expression (*RPL3*, *RPS8*, *RPL11*). We identified 209 differentially expressed genes/proteins (DEGPs) with corresponding trends at the transcriptome and proteome levels. Most DEGPs were similarly altered in at least one other skin disease. Psoriasis-specific and non-specific DEGPs had distinct cytokine-response patterns, with only the former showing disproportionate induction by IL-17A in cultured keratinocytes.

**Conclusions:**

Our findings reveal global imbalance between the number of increased and decreased proteins in psoriasis lesions, consistent with heightened translation. This effect could not have been discerned from mRNA profiling data alone. High-confidence DEGPs were identified through transcriptome-proteome integration. By distinguishing between psoriasis-specific and non-specific DEGPs, our analysis uncovered new functional insights that would otherwise have been overlooked.

**Electronic supplementary material:**

The online version of this article (doi:10.1186/s13073-015-0208-5) contains supplementary material, which is available to authorized users.

## Background

Psoriasis is a common inflammation-driven disease affecting 2–3 % of adults with direct and indirect costs totaling 35 billion dollars in the United States alone [1, 2]. Psoriasis lesions develop as a consequence of abnormal keratinocyte (KC) proliferation, which may be driven by key pro-inflammatory cytokines, including tumor necrosis factor (TNF), interleukin (IL)-17A and IL-23 [3]. Such cytokines are manufactured by immune cells that infiltrate psoriasis lesions (e.g., T cells, macrophages and neutrophils), creating a pathological cascade of events in which immunocytes, cytokines and KCs interact to promote lesion development [4]. To better understand this process, mRNA profiles from psoriasis lesions have been extensively studied using microarray or RNA-seq technology [5–8]. Such transcriptome studies have uncovered numerous protein-coding mRNAs with altered expression in psoriasis lesions, which has helped to better define cellular pathways activated within lesions, suggesting new disease mechanisms and possible drug targets [5–8]. Nonetheless, it cannot be assumed that changes in mRNA expression reliably predict changes in protein abundance or activity [9–13]. Ultimately, therefore, transcriptome–proteome integration is needed to fully understand psoriasis from a systems biology standpoint. This can reduce false-positive identifications and extend transcriptome findings by identifying those differentially expressed mRNAs impacting protein abundance, which may be more consequential for the disease process [14, 15].

The complexity of the human proteome exceeds that of the transcriptome, with more than 290,000 unique human peptides having been identified in one large-scale effort [16]. Such molecular diversity is driven in part by alternative splicing, which can generate thousands of protein isoforms from a single mRNA molecule [17]. In normal human skin, the exact number of proteins remains uncertain, but collagen chains, elastin, cytoskeletal keratins and vimentin are most abundant [18]. Using two-dimensional (2D) gel electrophoresis, an early study identified 21 skin proteins, including eight proteins with 2-fold increased abundance in psoriasis lesions (SERPINB4, GSTP1, SERPINB5, ARHGDIA, HSPB1, KRT14, KRT17, YWHAQ) and two others with 2-fold decreased abundance (KRT15, CALR) [19]. A subsequent study used 2D electrophoresis combined with liquid chromatography tandem mass spectrometry (LC-MS/MS) to identify 36 proteins elevated 2-fold in psoriasis lesions (e.g., GSTP1, SFN, PRDX2), which were functionally related to diverse processes, such as apoptosis, defense response, inflammatory response, redox balance and cell proliferation [20]. More recently, isobaric tag for relative and absolute quantitation (iTRAQ) labeling was used to identify 1217 proteins in laser capture microdissected epidermis from psoriasis lesional and uninvolved skin [21]. Of these 1217 proteins, 241 were differentially expressed within lesions, many of which (25/214) were associated with IL-1B signaling [21]. This trend appears to agree well with transcriptome studies, which have also shown that genes differentially expressed in psoriasis lesions include a disproportionate number of IL-1B targets [8, 22].

The association between mRNA and protein abundance depends on many factors, including post-transcriptional mechanisms, translation rates, mRNA/protein half-life, and the intracellular localization of mRNAs and their associated proteins [9–11]. Despite apparent agreement between mRNA and protein studies of psoriasis, formal comparisons to quantitatively assess transcriptome-proteome correspondence have not been carried out. This has been difficult for two reasons. First, early studies using 2D gel electrophoresis could only identify a small number (<100) of highly abundant proteins [19, 20], compared with the thousands of protein-coding mRNAs detected by microarray or RNA-seq [5–8]. Second, prior studies have not applied transcriptome and proteome analysis to the same human samples, which would allow transcriptome–proteome comparisons on a sample-specific basis, thereby accounting for molecular-level heterogeneity among psoriasis lesions [23]. In human and mouse, mRNA–protein abundance correlations (*r*) have often been in the range of 0.40–0.60 [24], although broader ranges of correlations have been noted in other species or in cells under stress conditions (e.g., 0.04 < *r* < 0.95) [10]. Along these lines, psoriasis lesions can be viewed as a stressful cellular microenvironment, with reactive oxygen species that may have an impact on the stability of proteins and their higher-order conformations [20, 25]. Beyond this, mRNA stability and translation rate may be influenced by structural features, such as gene length and GC content, which were previously shown to associate with gene expression shifts in psoriasis [26]. Potentially, such unique features of psoriasis lesions may limit our ability to predict shifts in protein abundance based upon changes in mRNA alone.

In this study, we used RNA-seq and LC-MS/MS to profile mRNA and protein expression within the same set of lesional (PP) and uninvolved (PN) skin samples (*n* = 14 patients). This allowed us to assess correspondence between the psoriasis transcriptome and proteome with respect to individual samples and patients. Our analysis identified mRNA–protein pairs with discordant and concordant abundance shifts in the comparison between lesions and uninvolved skin. This allowed us to identify functionally related groups of genes for which transcriptome data do not accurately predict protein abundance. Additionally, from among thousands of mRNAs with significantly altered expression in psoriasis lesions, we delineated a narrowed set of differentially expressed mRNAs with significantly altered protein abundance. These “differentially expressed genes/proteins” (DEGPs) represent high-confidence targets for future studies of psoriasis disease mechanisms, therapies and biomarkers.

## Methods

### Ethics statement

All samples were obtained with informed written consent from volunteer patients in accordance with Declaration of Helsinki principles. All protocols were approved by an institutional review board (University of Michigan, Ann Arbor, MI, IRB number HUM00037994).

### Patient cohort

Skin biopsies were obtained from a cohort of 14 patients (October 2008 to August 2014), which included eight males and six females between the ages of 24 and 71 years (mean age 49.1 years; Additional file 1). Patients discontinued systemic therapies for 2 weeks prior to biopsy collection (1 week for topical treatment). Two 6 mm punch biopsies were collected from each patient following local lidocaine injection. Paired samples from each patient included one lesional (PP) skin biopsy and one uninvolved (PN) biopsy of macroscopically normal skin (sun-protected buttock or upper thigh region). Uninvolved biopsies were at least 10 cm away from any active psoriasis lesion. Each biopsy was divided into two sections and each section was flash frozen in liquid nitrogen and stored at −80 °C. The two sections were subsequently processed independently, with one section used for RNA extraction and the other used for total protein extraction (described below).

### High-throughput sequencing (RNA-seq)

RNA extraction was performed using RNAeasy columns (Qiagen) starting with one-half of each biopsy. RNA quality was subsequently evaluated using the Agilent 2100 Bioanalyzer (Agilent Technologies), which revealed intact ribosomal RNA profiles (18S and 28S) for all samples. Sequencing of fragmented cDNA was performed using the Illumina Genome Analyzer IIx. This yielded an average of 29.3 million 50 bp reads per sample (range: 22.3–42.2 million; Additional file 2a). Illumina adaptor sequences were removed using CutAdapt (version 1.6) with a maximum error rate setting (−e) of 5 % and a minimum post-processing read length (−m) of 20 bp [27]. An initial quality check of reads was performed using FastQC [28], which indicated nucleotide bias affecting the initial 10 bp at the 3′ end of reads. These 10 bases were removed using the “fastx_trimmer” function from the FASTX-Toolkit [29], after which a second FastQC analysis confirmed lack of nucleotide bias along the entire length of reads. Reads were then passed through running sum and window-based trimming functions [30]. First, reads were filtered using the CutAdapt running sum filter, using a PHRED quality threshold of 30 (−q) and minimum sequence length (−−minimum-length) of 20 bp [27]. Second, we applied the window-based “fastq_quality_filter” to retain only those reads with PHRED quality greater than 30 for at least 50 % of the read length [29]. Finally, reads were passed through the “fastx_artifacts_filter” function [29]. Following these preprocessing steps, there remained an average of 28.6 million reads per sample (range 21.9–41.3 million; Additional file 2b).

Filtered reads were mapped to the University of California, Santa Cruz (UCSC) human genome (hg19) using TopHat2 (version 2.0.12) [31]. Read mapping was performed using UCSC gene model annotations supplied as a GTF file, with multi-mapping of reads disallowed (i.e., using the –g 1 option). TopHat2 alignment files were sorted and indexed using SAMtools (version 0.1.18) [32]. Mapping quality was assessed using RSeQC and RNA-SeQC [33, 34], which indicated an average read mapping rate of 95.4 % (Additional file 2c), with 92.7 % of reads mapped to intragenic sequences (Additional file 2d) and an average coverage per base of 9.84 reads (Additional file 2e). Overlap between mapped reads and known human genes was tabulated using the htseq-count function from the HTSeq python library (version 0.5.4p3) [35]. Reads excluded from tabulation included those ambiguously associated with more than one feature (i.e., −m intersection-strict) as well as any reads with quality score lower than 10 (−a 10) [35]. Cufflinks (version 2.2.1) was used to calculate fragments per kilobase of exon per million fragments mapped (FPKM) for each gene feature [36]. Counts and FPKM estimates for human genes (hg19) are available from Gene Expression Omnibus (GEO; GSE67785) and raw sequence data are available from the Sequence Read Archive (link to GEO submission [37]).

The UCSC hg19 genome annotation includes coordinates for 19,225 protein-coding human genes. Of these, our analyses include only those genes for which expression was detected with respect to at least 25 % of PP and PN samples (i.e., at least 7 of the 28 samples). Two criteria were applied to determine whether a gene feature had detectable expression in a given sample [26]: first, a count per million mapped reads (cpm) greater than 0.25 was required; secondly, the lower bound on the 95 % FPKM confidence interval generated by Cufflinks needed to be greater than zero. Applying these criteria yielded 15,616 skin-expressed protein-coding genes. After skin-expressed genes were identified, samples were clustered based upon FPKM values for the 15,616 genes. This yielded the expected pattern, with clear separation between lesional and uninvolved skin samples, suggesting an absence of outliers (Additional file 2f). This conclusion was also supported by plotting the 28 samples with respect to the first two principal component axes calculated from FPKM values (Additional file 2g). Finally, for each patient, we examined fold changes (PP/PN) for genes shown to have altered expression in psoriasis lesions in a recent meta-analysis of microarray datasets (*n* = 237 patients) [38]. Based upon meta-analysis fold changes, we identified the top 100 PP-increased and top 100 PP-decreased genes; inspection of PP/PN fold changes for these genes in our current dataset yielded the expected trends for all 14 patients (Additional file 2h).

Differentially expressed genes (DEGs) with significantly altered expression in PP versus PN skin were identified using edgeR (*n* = 14 paired PP and PN samples) [39]. Raw gene counts were normalized using weighted trimmed mean of M-values (i.e., calcNormFactors method “TMM”) [39]. For each skin-expressed gene, dispersions were estimated using the Cox-Reid-adjusted likelihood method (function estimateGLMTrendedDisp) followed by fitting of negative binomial generalized log-linear models (function glmFit). Models were fit with two covariates (patient and sample type) and differentially expressed genes were identified using likelihood ratio tests (function glmLRT), in which the log-likelihood was compared between the full model (both covariates) and reduced model (only patient as a covariate) [39]. To control the false discovery rate (FDR), raw *p* values were adjusted using the Benjamini-Hochberg procedure [40].

### Liquid chromatography-tandem mass spectrometry

The second half of each skin biopsy was processed for LC-MS/MS analysis. Processing steps included custom protein extraction, SDS-PAGE, manual unbiased band excision, and in-gel digestion with trypsin. Each sample was washed twice with 0.5 mL phosphate-buffered saline. Samples were homogenized by mechanical disruption in a Bullet Blender (NextAdvance) with 0.8 mL urea lysis buffer (100 mM HEPES, pH 8.0, 1× Roche Complete, 1× Roche PhosStop) and 0.5 mm stainless steel beads. The homogenate was centrifuged to pellet the beads and debris. The supernatant was removed and heated at 100 °C for 15 minutes. The sample was centrifuged at 15,000 g for 10 minutes and supernatant removed. Protein quantification was performed on the extracted material using a Qubit fluorometry assay (Invitrogen).

Each sample (10 μg) was processed by SDS-PAGE using a 10 % Bis-Tris NuPAGE gel (Invitrogen) with MES buffer system. The gel was run approximately 2 cm. The mobility region was excised into 20 equal-sized segments and in-gel digestion was performed on each using a robot (ProGest, DigiLab) with the following protocol: (1) washed with 25 mM ammonium bicarbonate followed by acetonitrile; (2) reduced with 10 mM dithiothreitol at 60 °C followed by alkylation with 50 mM iodoacetamide at RT; (3) digested with trypsin (Promega) at 37 °C for 4 h; (4) quenched with formic acid and the supernatant was analyzed directly without further processing.

Each gel digest was analyzed by nano LC-MS/MS with a Waters NanoAcquity HPLC system interfaced to a ThermoFisher Q Exactive. Peptides were loaded on a trapping column and eluted over a 75 μm analytical column at 350 nL/minute; both columns were packed with Jupiter Proteo resin (Phenomenex; injection volume 30 μL). The mass spectrometer was operated in data-dependent mode, with the Orbitrap operating at 60,000 FWHM and 17,500 FWHM for MS and MS/MS respectively. The 15 most abundant ions were selected for MS/MS.

### LC-MS/MS data analysis

Data were searched using a local copy of the Mascot search engine (Matrix Sciences Inc.) with the following parameters: (1) enzyme trypsin/P; (2) database Uniprot Human (concatenated forward and reverse plus common contaminants); (3) fixed modification carbamidomethyl (C); (4) variable modifications oxidation (M), acetyl (N-term), pyro-Glu (N-term Q), deamidation (N/Q); (5) mass values monoisotopic; (6) peptide mass tolerance 10 ppm; (7) fragment mass tolerance 0.02 Da; (8) max missed cleavages 2. Mascot DAT files were parsed into the Scaffold software (Proteome Software Inc.) for validation, filtering and to create a non-redundant list per sample [41]. Data were filtered using 99 % probability for protein and 95 % probability for peptide (prophet scores), with 1.0 % FDR for protein and peptide probability requiring at least two unique peptides per protein.

Relative protein abundance was quantified using normalized spectral counts (i.e., normalized SpCs; Scaffold quantitative values) [42]. Absolute protein abundance was quantified using the normalized spectral abundance factor (NSAF). NSAF values were calculated using the equation NSAF = (SpC/MW)/Σ(SpC/MW)_N_, where SpC is spectral counts, MW is protein molecular weight in kDa, and N is the total number of proteins in one sample [43]. Among all 28 samples, there were 2454 proteins with SpC > 1 in at least one sample. For differential expression analyses, our analysis included 2232 proteins with SpC > 1 in at least 4 of the 28 samples. Of these 2232 proteins, 23 were associated with keratins included within the International Protein Index contaminants database (e.g., KRT2, KRT10, KRT15) [44]. These were retained in our analyses, however, since keratins are expected to be among the most abundant proteins in skin biopsies [18]. For each of the 2232 proteins, a least-squares regression model was fit to normalized SpC values, with covariates corresponding to patient and sample type (PP or PN; R function lm). Differentially expressed proteins (DEPs) were then identified based upon significance of regression coefficients associated with the sample type covariate. FDR-adjusted *p* values were calculated from raw *p* values using the Benjamini-Hochberg procedure [40].

### RT-PCR, western blot and immunohistochemistry

Select genes and proteins were further evaluated using RT-PCR, western blot and immunohistochemistry. These analyses were performed using an independent set of skin biopsies not evaluated by RNA-seq or LC-MS/MS, including lesional skin from psoriasis patients (PP), uninvolved skin from psoriasis patients (PN), and normal skin from control subjects (NN). Following RNA extraction (Qiagen RNeasy columns), RNA was reverse transcribed using the High Capacity cDNA Transcription kit (Applied Biosystems Inc., Foster City, CA, USA) and PCR was performed using the 7990HT Fast Real-Time PCR system (Applied Biosystems). Taqman primers were purchased from Life Technologies (catalog numbers 4331182 and 4351372; RPS8, Hs01374307_g1; RPS3A, Hs00832893_sH; RPL11, Hs00831112_s1; GAPDH, Hs99999905_m1; FABP5, Hs02339439_g1; SERPINB4, Hs01691258_g1). Western blots were performed using antibodies directed against RPL7A (Lifespan Biosciences, LS-C287612/60982, 1:1000 dilution), RPS8 (Lifespan Biosciences, LS-C192101, 1:500 dilution), EEF1A1 (Lifespan Biosciences, LS-C99327/61002, 1:1000 dilution), RPS3A (Lifespan Biosciences, PA5-29398, 1:500 dilution), RPL11 (Lifespan Biosciences, PA5-34604, 1:1000 dilution) and Beta-actin (Sigma, A5316, 1:10,000 dilution). For immunohistochemistry analysis, diaminobenzidine staining of paraffin embedded tissue was performed using anti-RPL7A (Lifespan Biosciences, LS-C287612/60982, 1:20 dilution), anti-FABP5 (R&D Systems, AF3077, 1:200 dilution), and anti-SERPINB4 (Lifespan Biosciences, LS-C172653, 1:200 dilution) antibodies.

### Integration with additional data resources

Primary data from the current study (RNA-seq and LC-MS/MS) were compared and integrated with data generated from large-scale proteomics projects [16, 18, 45, 46], as well as with gene expression datasets deposited within the GEO database [47]. Secondary proteomics data were obtained from supplemental files of research publications [16, 18, 45], or from project data deposited in the PRoteomics IDEntifications database (PRIDE; accession number PRD000053) [46, 48]. Normalized expression data were obtained either from GEO series matrix files or generated directly from raw CEL files (available as GEO supplemental files for Affymetrix datasets). Normalization of Affymetrix CEL files was performed using robust multichip average [49]. Normalized expression matrices were analyzed to identify DEGs, with comparisons performed between two treatments in all cases. This was done using linear model analysis with moderated t-statistics (R package limma) [50], yielding ordered gene lists for each two-treatment comparison, with genes ranked according to evidence for differential expression (i.e., using log_10_-transformed *p* values derived from linear models). A total of 2178 ordered gene lists were generated in this fashion, based upon an aggregate total of 21,337 unique GEO microarray samples (Additional file 3). Primary gene sets derived from the current study were screened against these 2178 ordered gene lists, which allowed us to identify experiments in which the genes were disproportionately increased or decreased [i.e., gene set enrichment analysis (GSEA)] [6, 51, 52].

## Results

### Detection of 15,616 protein-coding mRNAs (RNA-seq) and 2232 proteins (LC-MS/MS) in lesional and uninvolved skin (*n* = 14 patients)

The transcriptome and proteome of lesional (PP) and uninvolved (PN) skin biopsies from 14 psoriasis patients was analyzed using RNA-seq and LC-MS/MS, respectively. As expected, fewer proteins were detected using LC-MS/MS (2232) compared with the number of protein-coding mRNAs detected by RNA-seq (15,616) (see “Methods”). mRNAs associated with detected proteins were expressed at higher levels, with such mRNAs having FPKM values 10–17 times greater than other mRNAs (Additional file 4). We estimate that mRNAs with FPKM < 10 are associated with proteins for which abundance is usually below the LC-MS/MS detection threshold (Additional file 4).

Of 2232 detected proteins, we could match 2172 with a detected protein-coding mRNA. Among such mRNA–protein pairs, the average NSAF correlated with average FPKM (*r*_*s*_ = 0.52 and 0.45 for PP and PN skin, respectively; Additional file 5a, b). This correlation was lower with respect to individual patient samples (0.26 ≤ *r*_*s*_ ≤ 0.53; Additional file 6). The most abundant mRNAs encoded S100s (*S100A9*, *S100A8*, *S100A7*), keratins (*KRT10*, *KRT1*, *KRT14*) and ribosomal proteins (*RPS27*, *RPS6*, *RPS12*, *RPS8*) (Additional file 5c). The most abundant proteins, however, consisted almost exclusively of keratins (e.g., KRT10, KRT14, KRT1; Additional file 5d).

### Identification of 4122 DEGs and 748 DEPs in psoriasis lesions (*n* = 14 patients)

Comparison of gene expression between PP and PN skin biopsies yielded 4122 DEGs, including 1865 PP-increased DEGs (fold change > 1.50, FDR < 0.05) and 2257 PP-decreased DEGs (fold change < 0.67 and FDR < 0.05). Similarly, using LC-MS/MS, we identified 748 DEPs, including 616 PP-increased DEPs (fold change > 1.50 and FDR < 0.05) and 132 PP-decreased DEPs (fold change < 0.67 and FDR < 0.05). Thus, whereas similar numbers of PP-increased and PP-decreased DEGs were identified, we detected 4.7-fold more PP-increased DEPs than PP-decreased DEPs.

Comparisons between studies and technologies help to establish repeatability [53]. Using 2D gel electrophoresis, Carlén et al. [19] identified eight PP-increased proteins; however, we identified only three of these as PP-increased DEPs (SERPINB4, KRT17, SERPINB5) and as a group the eight proteins were not disproportionately elevated (*p* = 0.63; Additional file 7). Of two PP-decreased proteins identified by Carlén et al. [19], one was significantly decreased in our study (KRT15; *p* = 0.003; Additional file 6d) but the other (CALR) was not detected. Better agreement was obtained with respect to 36 PP-increased proteins identified by Ryu et al. [20] (2D gel electrophoresis). Of these 36, we identified 15 as PP-increased DEPs and as a group the 36 proteins were disproportionately elevated (*p* = 0.033; Additional file 8). Unexpectedly, 3 of the 36 were PP-decreased DEPs (SERPINF1, TF, APCS; Additional file 8).

Consistent with prior work [26], PP-decreased DEGs were significantly longer than PP-increased DEGs (Additional file 9a). This relationship was also observed among high-abundance mRNAs associated with LC-MS/MS-detected proteins (Additional file 9c). Despite this, however, PP-increased and PP-decreased DEPs did not differ significantly with respect to average molecular weight (*p* = 0.38; Additional file 9f).

### Shifts in mRNA and protein abundance show modest correlation in the comparison between psoriasis lesions and uninvolved skin (*r*_s_ = 0.40)

**Fig. 1:**
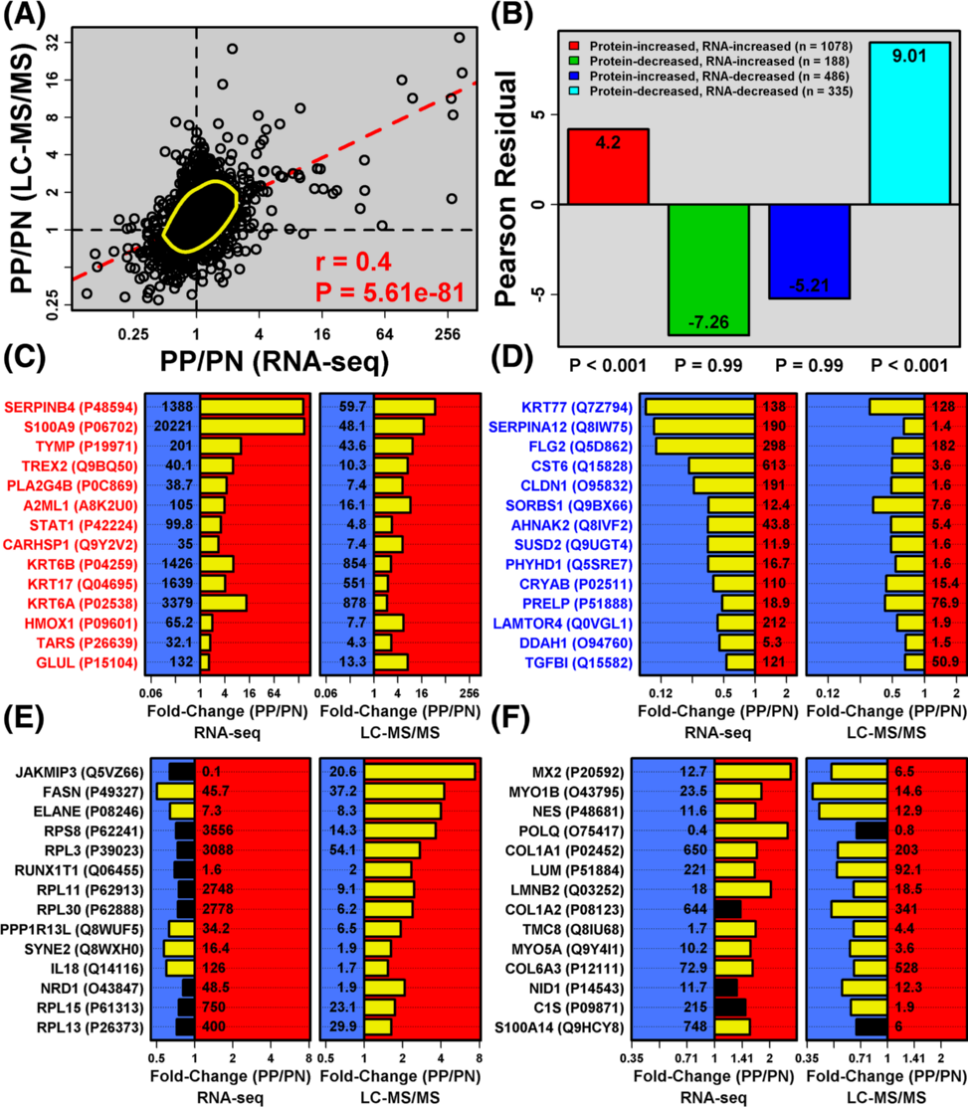
Shifts in mRNA and protein abundance show modest correlation in the comparison between psoriasis lesions and uninvolved skin (*r*_*s*_ = 0.40). **a** Correlation between fold changes (PP/PN) calculated by RNA-seq and LC-MS/MS (2172 mRNA–protein pairs). The *dashed red line* is a least-squares regression estimate and the *yellow ellipse* outlines the middle 50 % of data points nearest to the bivariate mean (Mahalanobis distance). **b** Pearson residuals. Each mRNA–protein pair was assigned to one of four groups (see legend). Residuals reflect the degree to which counts in each group (*n*) differ from those expected under the null hypothesis (i.e., random association between changes in mRNA and protein abundance). Positive residuals indicate over-abundance of mRNA–protein pairs, while negative residuals indicate under-abundance. **c** mRNA–protein pairs showing the strongest increase in mRNA and protein abundance. **d** mRNA–protein pairs showing the strongest decrease in mRNA and protein abundance. **e** mRNA–protein pairs with discordant changes in mRNA and protein abundance (PP-decreased mRNA; PP-increased protein). **f** mRNA–protein pairs with discordant changes in mRNA and protein abundance (PP-increased mRNA; PP-decreased protein). In (**c**–**f**), mRNA–protein pairs were chosen based upon the strength of *p* values derived from tests for differential mRNA and protein abundance (PP versus PN skin; *yellow bars* indicate DEGs/DEPs). Average FPKM or NSAF is listed at the base of each bar. These values were calculated for both PP and PN skin, respectively, and the higher of the two values is listed

Average fold changes (PP/PN) calculated by RNA-seq and LC-MS/MS were correlated across the 2172 mRNA–protein pairs (*r*_*s*_ = 0.40; Fig. 1a). Consistent with this, there was significant over-abundance of mRNAs/proteins altered in the same direction (Fig. 1b), and under-abundance of mRNAs/proteins altered in opposite directions (Fig. 1b). With respect to individual patients, the correlation between mRNA/protein fold changes was significant in 13 of 14 cases (*p* < 0.05; −0.01 ≤ *r*_*s*_ ≤ 0.35; Additional file 10). Fold change correlations were not weaker among low-abundance proteins (*r*_*s*_ = 0.44) compared with high-abundance proteins (*r*_s_ = 0.42) (Additional file 11).

xWe could identify individual genes and proteins showing concordance or discordance at the transcriptome and proteome levels (Fig. 1c–f). Increased mRNA–protein pairs included SERPINB4, S100A9, and TYMP, while decreased mRNA–protein pairs included KRT77, SERPINA12 and FLG2 (Fig. 1c, d). In contrast, FASN, ELANE and IL18 were decreased by RNA-seq but increased by LC-MS/MS (Fig. 1e). Conflicting trends were likewise observed for MX2, POLQ and S100A14 (increased by RNA-seq; decreased by LC-MS/MS; Fig. 1f).

### Translation machinery and ribosomal proteins are elevated in psoriasis lesions despite decreased mRNA levels

**Fig. 2:**
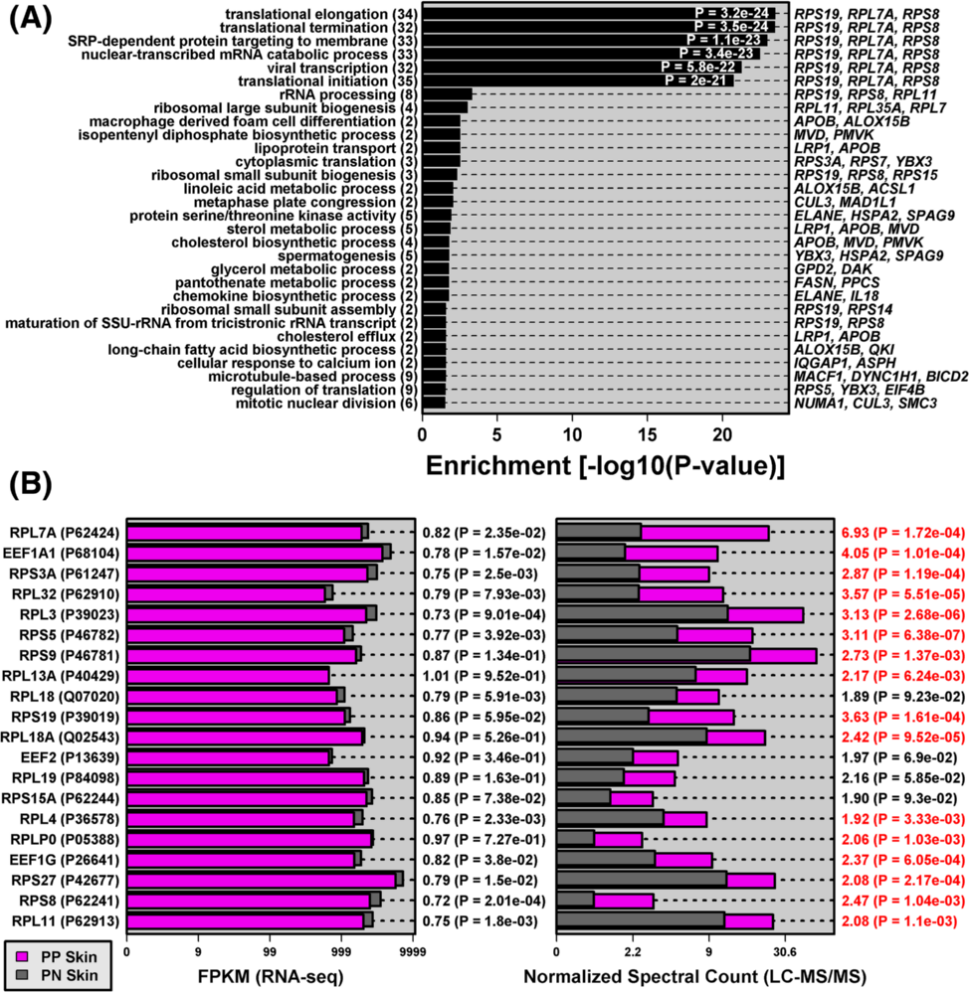
Translation machinery and ribosomal proteins are elevated in psoriasis lesions despite decreased mRNA levels. **a** GO biological process (BP) terms enriched among genes associated with 124 mRNA–protein pairs with discordant PP versus PN changes (i.e., PP-decreased mRNA, *p* < 0.10; PP-increased protein, *p* < 0.10). GO BP terms were enriched with respect to a background set of 2087 genes with detectable expression (RNA-seq) and associated with a protein identified by LC-MS/MS. **b** Genes and proteins associated with the “translational elongation” GO BP term. Fold-changes (PP/PN) and *p* values are listed in the right margin of each panel (*n* = 14 patients; red font indicates DEPs)

LC-MS/MS indicated that ribosomal subunit proteins were elevated in psoriasis lesions, even though corresponding mRNAs were decreased or unaltered according to RNA-seq (Fig. 1e). This was indicative of a broader trend involving translation-associated mRNAs and proteins. We identified 124 mRNA–protein pairs for which the mRNA tended to be decreased in psoriasis lesions (*p* < 0.10), even though the associated protein was increased (*p* < 0.10). Gene ontology (GO) analysis of the 124 genes showed enrichment for biological process terms such as translational elongation, translational termination, translational initiation and rRNA processing (Fig. 2a). Among mRNA–protein pairs associated with translational elongation, several were PP-increased DEPs for which the corresponding mRNA was decreased (RPS3A, RPS5, RPS27, RPL11 and RPS8; Fig. 2b). Using independent patient samples, we confirmed that mRNA expression of *RPS8*, *RPS3A* and *RPL11* is decreased in lesional skin (RT-PCR; Additional file 12a–c). In contrast, for several translation-associated proteins (RPL7A, RPS8, EEF1A1, RPS3A, RPL11), western blot indicated similar or increased abundance in lesional skin (Additional file 12d), with heavy staining of ribosomal protein in the lesional epidermis (Additional file 12e). Within psoriasis lesions, therefore, changes in mRNA expression do not predict shifts in the abundance of translation-associated proteins.

### Proteins expressed by immunocytes, developing tissues and transformed cell lines are biased towards increased expression in psoriasis lesions

Gene expression in full-thickness biopsies of psoriasis lesions is influenced by cellular composition and may, therefore, be associated with KC proliferation and/or infiltration of lesions by immunocytes [6–8, 23]. To address this at the protein level, we assessed whether proteins associated with specific cell populations or tissues are biased towards increased or decreased expression in lesions. For this purpose, we utilized proteome expression atlases, including a skin anatomy atlas [18], a human proteome map [16], the ProteomicsDB database [45], and the CPL/MUW database [46].

**Fig. 3:**
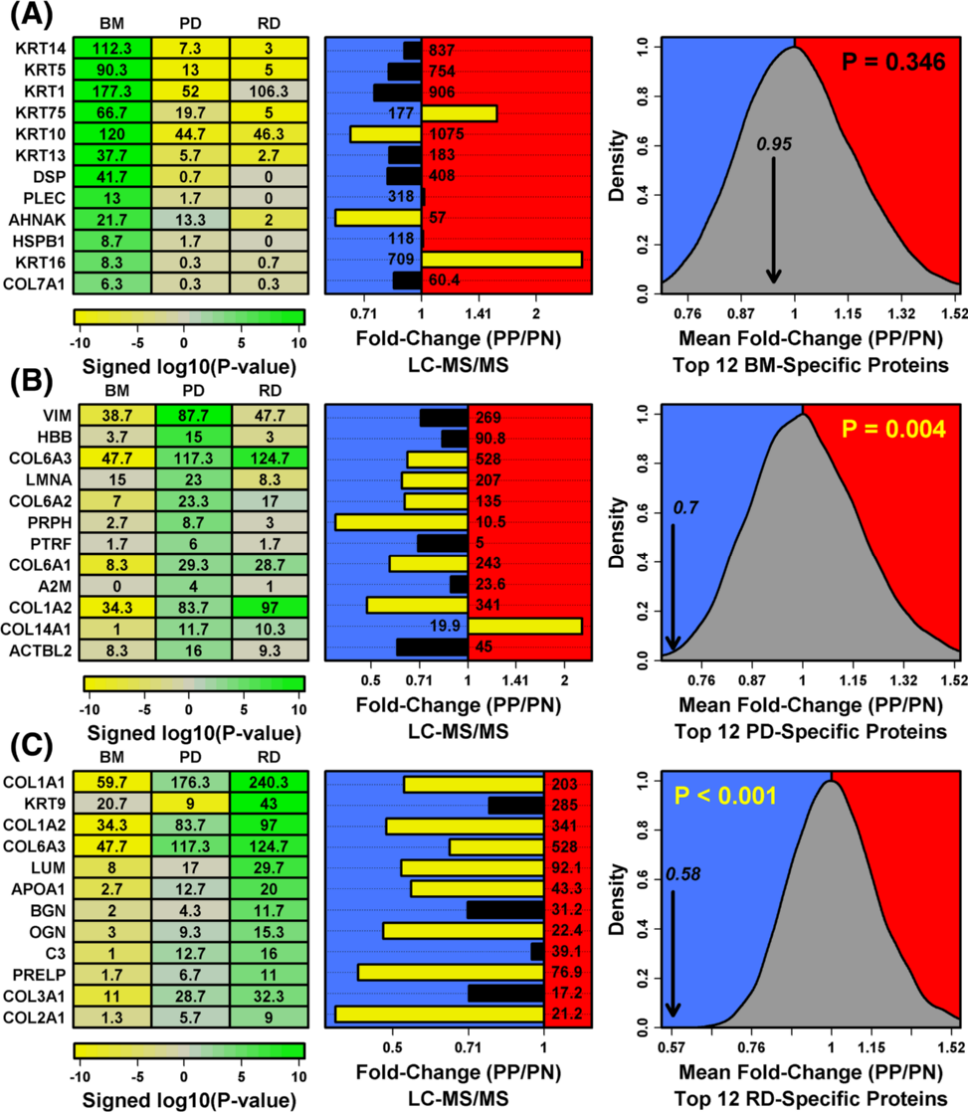
Proteins specifically expressed in papillary and reticular dermis are decreased in psoriasis lesions (skin anatomy atlas). Proteomic skin atlas data were used to identify proteins specifically expressed in basement membrane (*BM*) (**a**), papillary dermis (*PD*) (**b**), and reticular dermis (*RD*) (**c**). *Left*: Abundance of proteins is compared between compartments. Heatmaps show log_10_-transformed signed *p* values for the 12 proteins most specific to each compartment, where *p* values are derived from least-square regression models comparing abundance of proteins in each compartment to abundance in the other two (negative values indicate lower abundance; positive values indicate higher abundance). *Middle*: LC-MS/MS-estimated fold-changes (PP/PN) for each protein (*n* = 14 patients; *yellow bars* indicate DEPs). Average NSAF was calculated for PP and PN samples, respectively, and the higher of the two NSAF values is listed at the base of each bar. *Right*: Average fold change (PP/PN) was calculated for the 12 compartment-specific proteins (*black arrow*). This average value was compared with that obtained in 10,000 simulation trials in which 12 proteins were sampled randomly from 2194 proteins detected by LC-MS/MS. *P* values were calculated using the grey null distribution generated from the 10,000 simulation trials

Most DEGs from expression studies have been skin-specific, often associated with KCs or fibroblasts [6–8, 23]. We therefore first used a human skin proteome atlas to understand how shifts in protein abundance relate to skin microanatomy [18]. Proteins specific to the basement membrane region (KRT14, KRT5, KRT1) were decreased only slightly in psoriasis lesions with no significant overall trend (*p* = 0.346; Fig. 3a). In contrast, proteins specifically expressed by either the papillary or reticular dermis were significantly biased towards decreased expression (COL6A3, LMNA, COL1A1, COL1A2; *p* ≤ 0.004; Fig. 3b, c). Dermis-derived proteins are therefore biased towards decreased expression in psoriasis, in agreement with observations at the mRNA level [6–8, 23].

**Fig. 4:**
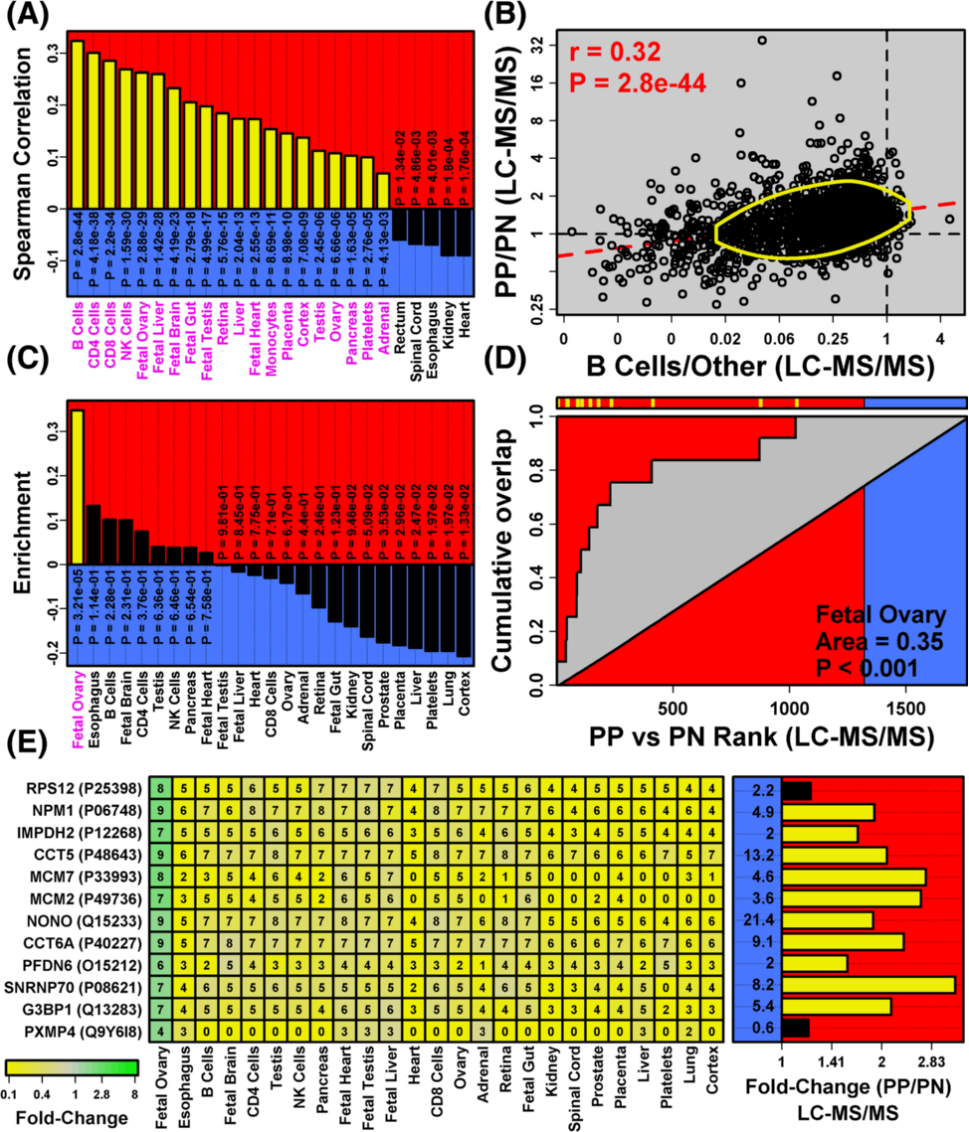
Proteins expressed in immunocytes and fetal tissues are elevated in psoriasis lesions (human proteome map). **a** Human proteome map cell types ranked by the correlation between cell type-specific expression and LC-MS/MS-estimated fold-change (PP/PN) (1764 proteins; *magenta font* indicates *p* < 0.05). **b** Association between B-cell-specific expression and fold change (PP/PN). B-cell-specific expression is quantified based upon the ratio of SpC values between B cells and all other cell types (*B cells/Other*, *horizontal axis*). The *dashed red line* is the least-square regression estimate and the *yellow ellipse* outlines the 50 % of proteins nearest to the bivariate mean (Mahalanobis distance). **c** Human proteome map cell types ranked according to how strongly the 12 best signature proteins for each cell type are enriched among proteins with elevated abundance in PP skin (*magenta font* indicates *p* < 0.05). **d** Enrichment of fetal ovary signature proteins among PP-increased proteins. The 1764 proteins were ranked in descending order from left to right based upon the LC-MS/MS-estimated fold change (PP/PN). The figure shows cumulative overlap between this ranked list of proteins and the 12 fetal ovary signature proteins (*vertical axis*). The ranking of each signature protein is indicated (*yellow hash marks*, *top*). Enrichment of the 12 proteins among PP-increased proteins is demonstrated by the positive area between the cumulative overlap curve and diagonal. **e** Fetal ovary signature proteins and their relative abundance across human proteome map cell types (heatmap of normalized SpC values). The LC-MS/MS-estimated fold change (PP/PN) is shown for each protein (*right*; *yellow bars* indicates DEPs). Average NSAF in PP samples is listed at the base of each bar

LC-MS/MS may be insensitive to low-abundance proteins derived from minority cell types, such as infiltrating immune cells. Surprisingly, however, analysis of human proteome map data showed that immunocyte-specific protein expression correlated positively with LC-MS/MS-estimated fold changes (PP/PN), with strong trends observed for several lymphocyte subsets (i.e., B cells, CD4 T cells, CD8 T cells and natural killer cells; Fig. 4a, b). The human proteome map, for instance, includes 1764 proteins detected by our analysis; among these, there was significant positive correlation between B-cell-specific expression and LC-MS/MS-estimated PP/PN fold changes (*r*_*s*_ = 0.32; *p* = 2.8 × 10^−44^; Fig. 4b). Proteins abundant within lymphoid organs (lymph node and spleen) were also elevated in psoriasis lesions (Additional file 13; ProteomicsDB). These trends suggest that, despite their low abundance, proteins derived from immune cells in psoriasis lesions are detected using LC-MS/MS.

Psoriatic KCs are proliferative and do not complete terminal differentiation as observed in normal skin [54–56]. Two signals from our data were consistent with this. First, proteins expressed by fetal tissues were biased towards increased expression in psoriasis lesions (Fig. 4a). The 12 proteins most specifically expressed in fetal spleen, for instance, all trended towards increased abundance (e.g., RPS12, NPM1, IMPDH2; Fig. 4e). Second, proteins expressed by transformed cell lines were elevated in lesions (e.g., Hep3B, HepG2 and jurkat; Additional file 14). Of 12 signature HepG2 proteins, for example, nearly all trended towards increased abundance (e.g., GADD45GIP1, ILF3, DNAJB1; Additional file 14e). Proliferation and transformation proteins expressed by undifferentiated cells are thus elevated in psoriasis lesions.

### Identification of 209 DEGPs with consistent changes in mRNA/protein abundance in psoriasis lesions versus uninvolved skin

**Fig. 5:**
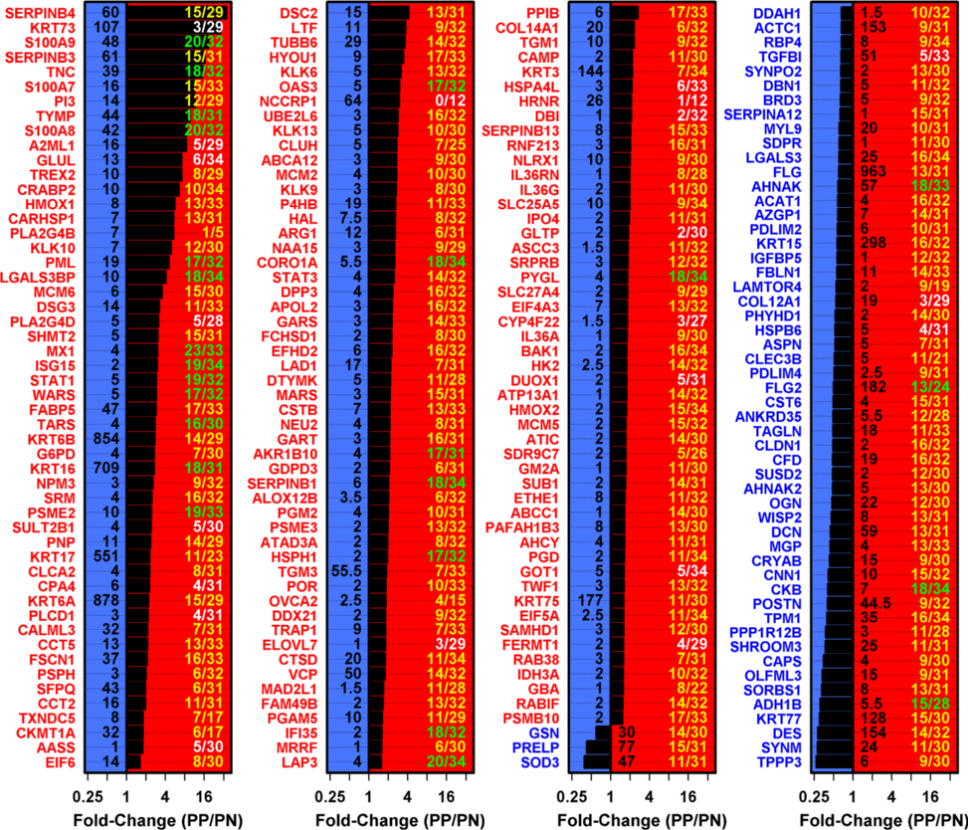
Differentially expressed genes/proteins with consistent changes in mRNA/protein abundance in psoriasis lesions versus uninvolved skin. We identified 209 DEGPs with corresponding and significant changes in mRNA and protein abundance in PP skin compared with PN skin (*n* = 14 patients). The figure lists the 209 DEGPs along with LC-MS/MS-estimated fold changes (PP/PN; *horizontal axis*). Average NSAF is listed at the base of each bar. Average NSAF was calculated with respect to PP and PN samples, respectively, and the higher of the two average values is listed. The fraction listed for each protein (*right*) indicates the proportion of skin diseases evaluated in which a corresponding change in mRNA expression was observed (*white font* indicates relatively psoriasis-specific; *green font* indicates relatively non-specific). The fraction denominator differs among DEGPs because skin diseases were analyzed using various microarray platforms, with some platforms lacking probes for assaying expression of certain DEGPs

Numerous mRNAs with altered expression in psoriasis lesions (DEGs) have been identified by microarray/RNA-seq studies [5–8], but it cannot be assumed that such DEGs encode DEPs [9–13]. In this study, changes in mRNA were only modestly correlated with shifts in protein abundance (*r*_*s*_ = 0.40; Fig. 1a). We therefore intersected the 4122 DEGs and 748 DEPs to define 209 DEGPs with corresponding and significant transcriptome and proteome trends (Fig. 5). DEGPs provide a high-confidence molecular fingerprint of psoriasis and, as expected, we confirmed changes in mRNA and protein abundance for select DEGPs (FABP5 and SERPINB4) using RT-PCR and immunohistochemistry (Additional file 15). GO biological process terms enriched among PP-increased DEGPs included defense response to virus, response to type I interferon, cell death, regulation of MHC class I biosynthetic process, neutrophil aggregation and establishment of skin barrier (*p* < 0.05; Additional file 16a). Similarly, terms enriched among PP-decreased DEGPs included cell growth, response to superoxide, tissue development and organ morphogenesis (*p* < 0.05; Additional file 16b).

### GSEA screening of ordered gene lists provides DEGP biomarker assessment and functional characterization in skin-associated cell types

GO terms are generic with regard to cell type and thus may not provide insights into cell type-specific gene functions. We therefore assembled 2178 ordered gene lists, where each list was derived from a microarray comparison performed using a skin-relevant cell type (Additional file 3). GSEA was then used to screen gene lists and identify experiments in which PP-increased and PP-decreased DEGPs were altered but in opposite directions (Additional file 17).

Psoriasis plaque development proceeds in coordination with an underlying cytokine network [4, 57, 58]. Consistent with this, we identified cytokines that induce PP-increased DEGPs and repress PP-decreased DEGPs in cultured KCs (e.g., IL-20, IL-19, IL-24, IL-22, IL-17A, IL-17C; Additional file 17a). Differences among these cytokines, however, were subtle, with each cytokine tending to be similarly effective at inducing psoriasis-like changes in gene expression (Additional file 17a). Analysis of KC gene perturbation experiments revealed that DEGP expression is positively associated with activity of *TP63*, *ERK1*, and *FSTL1*, but negatively associated with activity of *SNAI2*, *ETS1*, and *RELA* (Additional file 17b). DEGPs showed psoriasis-like changes in expression when KCs were treated with cathelicidin antimicrobial peptide (LL37) or scratched (wounded) in culture; conversely, DEGPs showed psoriasis-opposite changes in expression when KCs were treated with human papillomavirus oncoprotein E6, heparanase inhibitor (BIPBIPU) or dexamethasone (Additional file 17c). Psoriasis-like changes in DEGP expression were also observed in fibroblasts transduced with OKSM reprogramming factors (OCT4, KLF4, SOX2, c-MYC) [59], demonstrating pathway-level overlap between psoriasis and induced pluripotency (Additional file 17d).

DEGPs can provide psoriasis biomarkers, which might prove useful for tracking treatment responses or validating psoriasiform mouse phenotypes [60–64]. To illustrate this, we evaluated changes in DEGP expression in lesions of patients receiving biologic treatment (e.g., etanercept, ixekizumab, guselzumab; Additional file 17e). As expected, PP-increased DEGPs were repressed with therapy and PP-decreased DEGPs were elevated. These trends were most prominent in patients following at least 2 weeks of biologic therapy, but could be discerned as early as 1 day following the start of etanercept therapy (Additional file 17e). Although length of treatment was more important than type, we could discern that most biologics and even UVB treatment (10 weeks) elicited psoriasis-opposite expression patterns more effectively than treatment with the SIRT1 activator SRT2104 (Additional file 17e). We next evaluated expression of DEGP orthologues in psoriasiform mouse phenotypes [64–68]. Differences among top-ranked mouse phenotypes were minor, but the most psoriasis-like changes in DEGP expression were observed in 12-O-tetradecanoylphorbol-13-acetate (TPA)-treated mice lacking chemokine decoy receptor D6 (D6-KO) [69], mice with KC-specific overexpression of the angiopoietin receptor Tie2 (K5-Tie2) [70], and mice with K14-driven deletion of GlcCer-synthesizing enzyme UDP-glucose:ceramide glucosyltransferase (UGCG; K14-Ugcg-KD) [71] (Additional file 17f).

### Most DEGPs are not psoriasis-specific but are similarly altered in skin cancers and/or lesions from other inflammatory skin diseases

Expression shifts in psoriasis lesions may be associated with disruption of homeostasis or cutaneous inflammation, which is characteristic of many skin conditions [6, 60, 72, 73]. For this reason, only a fraction of genes with altered expression in psoriasis lesions are expected to be psoriasis-specific [6, 60, 72, 73]. Consistent with this, we noted strong and significant overlap between DEGPs and sets of genes altered in other skin conditions, with the strongest overlap observed for Mediterranean spotted fever eschars, eczema, squamous cell carcinoma and acne (Additional file 17k). In fact, only one DEGP (NCCRP1) was not significantly and similarly altered in another skin disease (Fig. 5). All other DEGPs could be placed along a continuum, with some showing greater psoriasis specificity (e.g., DBI, GLTP, HRNR, KRT73, ELOVL7), and others showing similar expression shifts in many or most skin diseases (e.g., MX1, S100A8, S100A9, STAT1, LAP3) (Fig. 5).

### Stratified GSEA reveals that psoriasis-specific and non-specific DEGPs have divergent responses to IL-17A and pro-differentiation stimuli

Given that DEGPs showed varying degrees of psoriasis specificity, we asked whether functional properties of the most psoriasis-specific DEGPs differ from those of the least specific. These analyses were performed using only PP-increased DEGPs, since there were too few PP-decreased DEGPs to enable robust comparisons based on psoriasis specificity (Fig. 5).

**Fig. 6:**
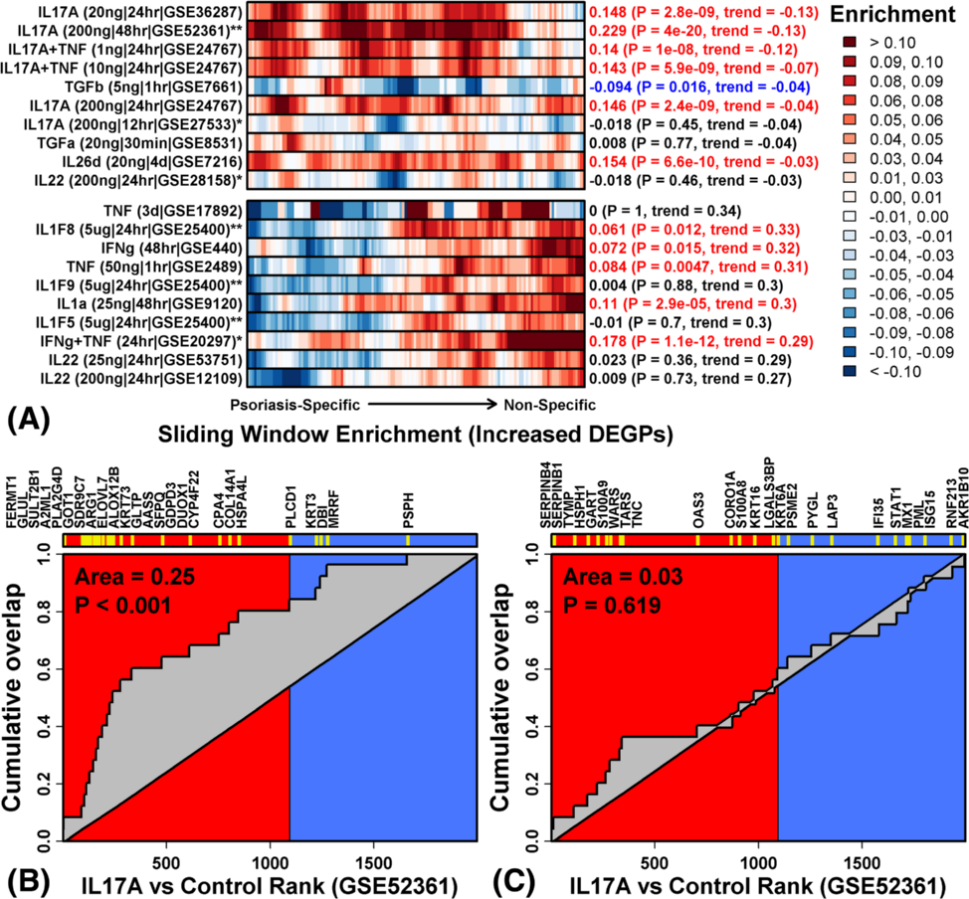
Psoriasis-specific and non-specific DEGPs have divergent cytokine responses (stratified GSEA). **a** Cytokine treatments that differentially impact expression of psoriasis-specific and non-specific DEGPs (cultured KCs; *HaCaT cells; **reconstituted epidermis). The 153 PP-increased DEGPs were ranked according to the percentage of skin diseases in which the mRNA’s expression was correspondingly increased. Sliding DEGP windows (10 DEGPs/window) were analyzed to assess enrichment of DEGPs among genes induced by the cytokine treatment (*red* indicates windows with cytokine-induced DEGPs; *blue* indicates windows with cytokine-repressed DEGPs). Robust regression was used to quantify the left-to-right trend in enrichment statistics, and listed experiments were associated with the most negative (*top*) or positive (*bottom*) enrichment statistic trends (out of 59 experiments screened). The concentration, treatment duration, and GEO series identifier is listed for each cytokine treatment. Enrichment statistics and *p* values were also calculated using the complete set of 153 DEGPs (*right margin*; *red*/*blue font* indicates FDR < 0.05, Wilcoxon rank sum test). **b** The 25 most psoriasis-specific PP-increased DEGPs are enriched among genes induced by IL17A (*p* < 0.001; *GSE52361*). Genes were ranked along the horizontal axis according to how strongly they are induced by IL17A (*left*/*red*, IL17A-induced; *right*/*blue*, IL17A-repressed). The figure shows cumulative overlap between the 25 psoriasis-specific DEGPs and this ranked gene list. **c** The 25 most non-specific PP-increased DEGPs are not enriched among genes induced by IL17A (*p* = 0.619; *GSE52361*). Genes were ranked along the horizontal axis according to how strongly they are induced by IL17A (*left*/*red*, IL17A-induced; *right*/*blue*, IL17A-repressed). The figure shows cumulative overlap between the 25 non-specific DEGPs and this ranked gene list

Using a “stratified GSEA” approach, we identified cytokine treatments that induce expression of the most psoriasis-specific DEGPs but not non-specific DEGPs. Six of the seven top-ranked cytokine treatments identified from this analysis involved IL-17A (Fig. 6a). Of the most psoriasis-specific DEGPs, the majority were induced by IL-17A (e.g., FERMT1, GLUL, SULT2B1; Fig. 6b); in contrast, the most non-specific DEGPs were not disproportionately induced by IL-17A and several were repressed (e.g., AKR1B10, RNF213, ISG15; Fig. 6c). In contrast to this trend, most other cytokines associated with the psoriasis gene expression profile in the current study (Additional file 17a), and in previous studies [6, 8, 22, 74], appeared to primarily target non-specific DEGPs; such cytokines include TNF, interferon (IFN)-γ, IFN-α and IL-1-family cytokines (e.g., IL1-F8, IL1-F9 and IL-1A; Fig. 6a).

**Fig. 7:**
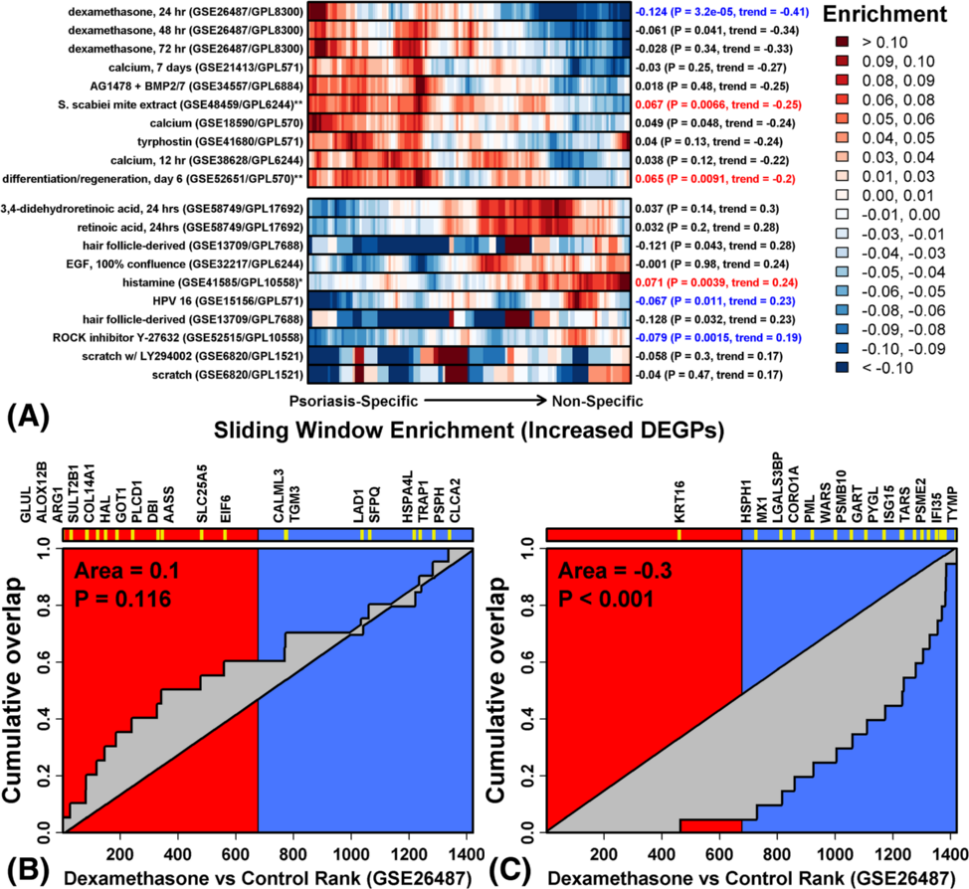
Psoriasis-specific and non-specific DEGPs have divergent responses to differentiation-associated stimuli (stratified GSEA). **a** Non-cytokine treatments that differentially impact expression of psoriasis-specific and non-specific DEGPs (cultured KCs; *HaCaT cells; **reconstituted epidermis). The 153 PP-increased DEGPs were ranked according to the percentage of skin diseases in which the mRNA’s expression was correspondingly increased. Sliding DEGP windows (10 DEGPs/window) were analyzed to assess enrichment of DEGPs among genes induced by each treatment (*red* indicates windows with induced DEGPs; *blue* indicates windows with repressed DEGPs). Robust regression was used to quantify the left-to-right trend in enrichment statistics, and the listed experiments were associated with the most negative (*top*) or positive (*bottom*) trends (out of 170 experiments screened). Enrichment statistics and *p* values were also calculated using the complete set of 153 DEGPs (*right margin*; *red*/*blue font* indicates FDR < 0.05, Wilcoxon rank sum test). **b** The 20 most psoriasis-specific PP-increased DEGPs are marginally enriched among genes induced by dexamethasone (*p* = 0.116; 48 h treatment, *GSE26487*). Genes were ranked along the horizontal axis according to how strongly they are induced by dexamethasone (*left*/*red*, dexamethasone-induced; *right*/*blue*, dexamethasone-repressed). The figure shows cumulative overlap between the 20 psoriasis-specific DEGPs and this ranked gene list. **c** The 20 most non-specific PP-increased DEGPs are enriched among genes repressed by dexamethasone (*p* < 0.001; 48 h treatment, *GSE26487*). Genes were ranked along the horizontal axis according to how strongly they are induced by dexamethasone (*left*/*red*, dexamethasone-induced; *right*/*blue*, dexamethasone-repressed). The figure shows cumulative overlap between the 25 non-specific DEGPs and this ranked gene list

Psoriasis-specific and non-specific DEGPs also showed divergent responses to treatments regulating KC differentiation (Fig. 7). Psoriasis-specific DEGPs were induced by treatments promoting KC differentiation, whereas non-specific DEGPs were repressed. Such pro-differentiation treatments included dexamethasone, calcium, epidermal growth factor receptor inhibitor (tyrphostin and AG1478) and epidermal regeneration (within devitalized human dermis matrix) (Fig. 7a). The most non-specific DEGPs, for example, were overwhelmingly repressed by dexamethasone (*p* < 0.001; Fig. 7c), but psoriasis-specific DEGPs tended to be increased (*p* = 0.116; Fig. 7b). In contrast, psoriasis-specific DEGPs were repressed by pro-proliferative treatments interfering with KC differentiation, whereas non-specific DEGPs were induced by these treatments (Fig. 7a). Consistent with these trends, psoriasis-specific and non-specific DEGPs were oppositely regulated by RNA interference treatments targeting pro- and anti-differentiation genes, respectively (e.g., *SNAI2*, *ANCR*, *STAU*, *ZNF750*; Additional file 18).

Our initial analysis identified a number of mouse skin phenotypes showing psoriasis-like changes in DEGP expression, but among top-ranked phenotypes the strength of this trend was similar and it was difficult to make distinctions quantitatively (Additional file 17f). Closer inspection using stratified GSEA, however, revealed that, for several mouse phenotypes, psoriasis-like changes in DEGP expression were primarily driven by activation of non-specific rather than psoriasis-specific responses (e.g., D6-KO, K5-Stat3c and K14-ADAM17-KO; Additional file 18b). Conversely, other mouse phenotypes manifested increased expression of DEGPs most specific to psoriasis (e.g., K5-Tie2, Krt1-KO and imiquimod; Additional file 18b). Applying GSEA to groups of DEGPs with differing psoriasis specificity thus uncovered trends not discerned from aggregate GSEA analysis of all DEGPs.

## Discussion

**Fig. 8:**
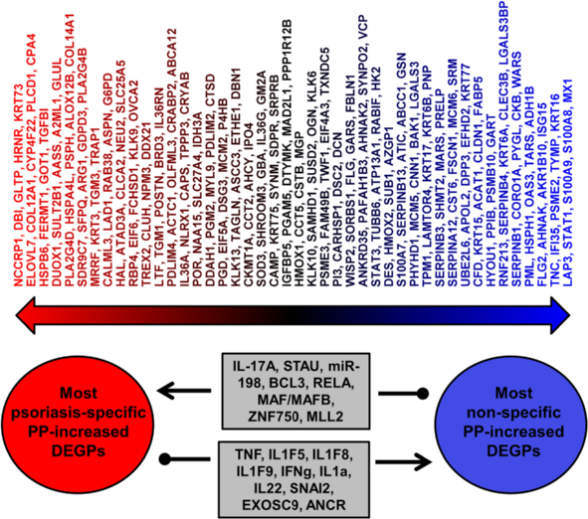
Cytokines and genes with differential effects on psoriasis-specific and non-specific DEGPs (cultured KCs). We identified 153 DEGPs for which RNA-seq and LC-MS/MS indicated elevated mRNA and protein abundance in psoriasis lesions. Nearly all DEGPs were similarly elevated in at least one other skin disease. Nonetheless, all DEGPs can be placed along a continuum separating those that are most psoriasis-specific (similarly altered in few other diseases) from those that are most non-specific (similarly altered in many other diseases). Analysis of microarray data using stratified GSEA revealed different cytokine response profiles and gene functional associations in these two groups (Figs. 6 and 7). The figure shows cytokines and genes that positively regulate expression of psoriasis-specific DEGPs but repress expression of non-specific DEGPs (e.g., IL-17A, STAU, miR-198). Conversely, the figure shows cytokines and genes that repress expression of psoriasis-specific DEGPs but induce expression of non-specific DEGPs (e.g., TNF, IL-1F5, IL-1F8). *Arrows* denote activation and *round-tipped lines* denote repression

Technological advances have profoundly improved our ability to measure mRNA abundance, but proteins are the direct determinants of health and disease. We thus performed the first study using RNA-seq and LC-MS/MS to interrogate the transcriptome and proteome of lesions from an inflammatory skin disease (psoriasis vulgaris). Our findings reveal moderate transcriptome-proteome correspondence, but also uncover “dark recesses” of psoriasis biology not illuminated by transcriptome analysis. Although RNA-seq did not reveal increased abundance of mRNAs associated with ribosome and translation proteins, LC-MS/MS indicated that peptides from such proteins are elevated in psoriasis lesions. By intersecting RNA-seq and LC-MS/MS findings, moreover, we could define 209 DEGPs showing consistent trends with both technologies. Subsequent bioinformatic analysis showed that most DEGPs exhibit similar mRNA expression shifts in other skin diseases. However, we noted disparities between DEGPs that are most and least psoriasis-specific, with only the former induced by IL-17A in cultured KCs (Fig. 8). Our findings thus uncover a new IL-17A signature, discernable at the transcriptome and proteome levels, which is prominent in psoriasis but attenuated or absent in other skin conditions.

Trademark features of psoriasis lesions include accelerated turnover of epidermal layers, aberrant KC differentiation, and enhanced proliferation of basal KCs [54, 55]. This proliferative phenotype requires heightened protein synthesis, which has previously been described in psoriasis but not fully understood [75, 76]. Freedberg first noted 7-fold elevated translation in psoriasis lesions and later suggested that such hyper-translation might be “a very central issue to the solution of the pathogenesis of psoriasis” [75]. This same author had indicated that increased translation may be attributable to deficiency of ribosome degradation, rather than de novo ribosome synthesis [76]. In agreement with this degradation-resistant ribosome hypothesis, LC-MS/MS showed that peptides associated with ribosomal proteins and elongation factors are more abundant in psoriasis lesions, even though encoding mRNAs are not elevated or decreased (Fig. 2; Additional file 12). Potentially, such ribosome-associated mRNAs are repressed in psoriasis lesions in response to increased abundance of the proteins they encode (i.e., negative feedback). Similar feedback mechanisms may operate genome-wide to limit transcriptome-proteome correspondence [77, 78], consistent with observations in this study (Fig. 1a) and some previously reported findings [9, 10].

Ribosomal protein accumulation may ultimately contribute to heightened translation by augmenting the quantity of protein generated per mRNA molecule in lesional skin, with global effects favoring increased rather than decreased protein abundance. Along these lines, we identified similar numbers of increased and decreased mRNAs, but 4.7-fold as many elevated (616) as decreased (132) proteins. Similar imbalances were observed in prior proteomic studies of psoriasis. Using 2D electrophoresis, for instance, Carlén et al. [19] identified eight PP-increased DEPs and two PP-decreased DEPs. Ryu et al. [20] did not report quantitative data, but identified 145 differentially expressed spots and noted that “most of the proteins were up-regulated.” These studies used a “top-down” proteomics approach differing from the “bottom-up” LC-MS/MS strategy we used. Such approaches differ in their resolution ability, sensitivity, and dynamic range [79], which may account for discrepancies between our study and earlier work at the level of individual proteins (e.g., see Additional files 7 and 8). Both technological strategies, however, support an imbalance that favors increased over decreased protein abundance in psoriasis lesions, consistent with heightened translational activity.

Previous studies have highlighted activation of the mammalian target of rapamycin (mTOR)/S6K pathway in psoriasis lesions [80–83], although our findings suggest that mechanisms governing hyper-translation in psoriasis are broader, involving proteins besides the canonical mTOR targets (Fig. 2). mTOR is a cellular hub that controls translation and cell growth through activation of target proteins, such as eukaryotic initiation factor 4E (eIF4E), eIF4E binding proteins (4E-BPs), and ribosomal S6 kinases (S6K1 and S6K2) [84, 85]. Within psoriasis lesions, mTOR kinase is elevated throughout the epidermis and activated phospho-mTOR (Ser2448) is prominent in the basal layer [80, 83]. Consistent with this, S6K1 (Thr389) is activated in psoriasis lesions [80] and ribosomal protein S6 is activated at multiple phosphorylation sites, with S6 (Ser235/236) more active in the suprabasal epidermis and S6 (Ser240) active throughout the epidermis and basal layer [80, 81]. An important question is whether targeted inhibition of these pathways will provide the basis for effective psoriasis treatment. Thus far, efforts to inhibit mTOR-associated pathways (e.g., rapamycin) have achieved only limited efficacy for treatment of plaque psoriasis [86, 87], although some efficacious topical agents are known to inhibit AKT/mTOR signaling [88, 89]. Rapalogs, next-generation mTOR inhibitors, and novel plant-derived phytochemicals thus continue to be investigated as possible mTOR-based psoriasis treatments [83, 90–92]. Based on our findings, an interesting possibility is that stronger therapeutic responses may be obtained by targeting specific stages of translation initiation, elongation or termination (Fig. 2). It is noteworthy, for instance, that cycloheximide, an inhibitor of translation elongation [93], is surprisingly well tolerated and effective as a topical treatment for psoriasis [94–96].

Transcriptome analysis of skin disease has provided a powerful tool and has so far identified thousands of differentially expressed mRNAs in psoriasis lesions (DEGs) [5–8]. It has seldom been possible, however, to investigate on a large-scale whether DEGs are in fact associated with DEPs. To address this, we overlapped RNA-seq and LC-MS/MS findings to identify 209 DEGPs with concordant mRNA and protein shifts in psoriasis lesions. These likely represent only a subset of true psoriasis DEGPs, since LC-MS/MS is not expected to comprehensively quantify all cellular proteins [42]. Nevertheless, DEGPs we identified can be viewed as DEGs with an additional layer of validation supporting their biological significance, affirming that DEGs are not false positives or associated with transient/unstable mRNAs that are only weakly translated. With DEGPs as a starting point, therefore, we used GSEA to screen 2178 ordered gene lists derived from microarray experiments performed with skin-associated cell types (Additional file 3). This data-driven approach uncovered experiments in which DEGPs were altered in a psoriasis-like or psoriasis-opposite fashion (Additional file 17). Two findings from this analysis were unexpected. First, we identified a psoriasis-like expression response in KCs treated in vitro with cathelicidin antimicrobial peptide (CAMP/LL37). CAMP was among PP-increased DEGPs identified in this study (Fig. 5) and may have important auto-antigen activity in psoriasis [97], either by forming complexes with self-derived nucleic acids to trigger innate immune responses [98], or by interacting with and stimulating T cells [99]. Secondly, induction of pluripotency in skin fibroblasts [59] generated shifts in DEGP expression mirroring those observed in human psoriasis (Additional file 17d). In a novel way, this result reflects the proliferative and de-differentiated status of psoriasis lesions, consistent with increased abundance of proteins expressed by fetal tissues (Fig. 4), transformed cell types (Additional file 14), and skin cancers (Additional file 17k).

Nearly all DEGPs we identified were similarly altered in other skin diseases besides psoriasis (Fig. 5). Expression shifts of DEGPs in psoriasis versus normal skin, for instance, paralleled those observed in the comparison between normal skin and eschars, eczema, and squamous cell carcinoma (Additional file 17). These conditions are mechanistically distinct from psoriasis, suggesting that DEGPs are to some degree associated with generic cutaneous responses, such as those arising from disrupted homeostasis and secondary inflammation [6, 60, 72, 73]. Based upon this, we used stratified GSEA, which identified microarray experiments in which expression of psoriasis-specific and non-specific PP-increased DEGPs are differentially altered (Figs. 6 and 7). This revealed that many cytokines previously associated with the psoriasis gene expression profile (e.g., TNF, IFN-a, IFN-g and IL-1 family) [6, 8, 22, 74] are primarily inducing a gene expression response that is not specific to psoriasis, but is instead associated with a broad spectrum of skin diseases (Figs. 6 and 8). In contrast, genes induced by IL-17A (or IL-17A plus TNF) in cultured KCs tend to be more psoriasis-specific and less commonly elevated in other skin conditions (Figs. 6 and 8). This unique aspect of IL-17A would have been overlooked by analyzing all PP-increased DEGPs in aggregate, without differentiating between psoriasis-specific and non-specific DEGPs. This result, however, resonates with clinical findings, which have emphasized that the most efficacious psoriasis therapies block IL-17A activity [100, 101], even when such treatments primarily target another cytokine such as TNF [102, 103].

We anticipate that the complete set of DEGPs identified in this study can be used as high-confidence biomarkers for future work. However, given distinct functional properties of psoriasis-specific and non-specific DEGPs (Fig. 8), it may be appropriate for such applications to assign greater emphasis to the most psoriasis-specific DEGPs, such as *NCCRP1*, *DBI* and *GLTP* (Fig. 5). For instance, as in previous studies [64], we here identified statistically significant similarities between the gene expression profile of human psoriasis and that of psoriasiform phenotypes in the laboratory mouse (Additional file 17f) [64–68]. We showed, however, that in some cases this similarity is driven by non-specific DEGPs, rather than those that are most specific to psoriasis (e.g., D6-KO, K5-Stat3c and K14-ADAM17-KO; Additional file 18b). Potentially, such mouse phenotypes may be useful as a model for any one of several human skin diseases, but may be less appropriately viewed as “psoriasis mouse models” per se. On the other hand, some mouse phenotypes exhibit psoriasis-matching expression patterns for those DEGs/DEGPs that are most psoriasis-specific (e.g., K5-Tie2, Krt1-KO and imiquimod; Additional file 18b). Potentially, these mouse phenotypes will better recapitulate unique mechanistic features of psoriasis lesion development, which appear to involve aberrant KC maturation and activation of IL-17A-directed pathways (Fig. 8). In these respects, findings from this study carry implications for a range of contexts in which selected mRNAs or proteins are employed as psoriasis biomarkers [60–63]. Such contexts may include the evaluation of psoriasis mouse models, but may also include the monitoring of treatment responses in psoriasis patients or screening of candidate drug compounds [60–63].

## Conclusions

Previous psoriasis studies have emphasized transcriptome analysis, leaving the psoriasis proteome less well explored and prompting the need for transcriptome–proteome integration. We thus used RNA-seq and LC-MS/MS to profile mRNA and protein abundance in the same set of skin samples from 14 psoriasis patients. This multi-omics strategy brings key aspects of psoriasis into focus, with three main implications for future work. First, in psoriasis and possibly other skin diseases, changes in mRNA and protein abundance are only modestly associated (*r*_*s*_ = 0.40). Transcriptome analysis alone, therefore, may not be sufficient for understanding key aspects of skin disease at the cellular level. Second, mechanisms underlying heightened translation in psoriasis lesions may be broader than currently understood, involving some proteins besides canonical mTOR targets (e.g., ribosomal subunits and translation factors). Potentially, therefore, therapies interfering with specific stages of translation may hold greater promise than treatments narrowly targeting mTOR/S6K. Finally, an unexpected finding was that DEGPs with psoriasis-specific and non-specific expression patterns have distinct functional properties. Only psoriasis-specific DEGPs, for example, were enriched with IL-17A targets. In gene expression studies of skin disease, therefore, stratified GSEA and other bioinformatic approaches to deconvolute disease-specific and non-specific signals may bring new insights into pathogenic mechanisms.

## Additional files

Additional file 1: 
**Clinical characteristics of psoriasis patients providing lesional and uninvolved biopsies for RNA-seq and LC-MS/MS analysis** **(** ***n*** **= 14).** The table lists sex, age range, body mass index (BMI), body weight (kg), psoriasis total body surface area (TBSA) and psoriasis area severity index (PASI). For some patients, information was absent from clinical records and is thus not included in the table (“unknown” entries). (PDF 120 kb) Additional file 2: 
**RNA-seq quality control.** RNA-seq was used to analyze 28 skin samples from 14 psoriasis patients (PP and PN skin). a Number of reads prior to filtering. b Number of reads after filtering. c Percentage of reads mapped to the UCSC human genome (hg19). d Percentage of reads mapping to intragenic regions. e Mean read coverage per genomic base. f Cluster analysis of the 28 samples. The dendrogram was generated using average linkage hierarchical clustering. Euclidean distance between samples was estimated based upon FPKM for the 15,616 protein-coding mRNAs detected in at least 25 % of samples (i.e., at least 7 of 28 samples). g Two dimensional principal component plot. The 28 samples were plotted with respect to the first two principal component axes. Principal component axes were calculated using FPKM values for the 15,615 protein-coding mRNAs. h Expression patterns of known psoriasis DEGs. PP-increased (*horizontal axis*) and PP-decreased DEG scores (*vertical axis*) were calculated for each patient, based upon DEGs identified from an earlier meta-analysis of microarray data (*n* = 237 patients). The PP-increased score (*horizontal axis*) is equal to the average fold-change (PP/PN) of the 100 DEGs most strongly elevated in the meta-analysis. The PP-decreased score (horizontal axis) is equal to the average fold change (PP/PN) of the 100 DEGs most strongly repressed in the meta-analysis. (PDF 3507 kb) Additional file 3: 
**Curated ordered gene lists from microarray experiments performed with skin-associated cell types.** We aggregated 2178 gene lists generated from microarray experiments included in Gene Expression Omnibus (GEO). Gene lists were derived from one of 11 types of experiments: a KCs stimulated with cytokines (59 lists; *HaCaT; **reconstituted epidermis); b KC gene perturbations (65 lists; *HaCaT; **reconstituted epidermis; “ + ” denotes overexpression of a gene rather than interfering RNA knockdown); c non-cytokine treatments applied to KCs (169 lists; *HaCaT; **reconstituted epidermis); d fibroblast treatments (182 lists; *non-primary cells); e psoriasis treatment responses (26 lists; *comparison to lesions prior to treatment rather than lesions from placebo-treated patients); f laboratory mouse phenotypes (35 lists); g Peripheral blood mononuclear cell (PBMC) treatments (461 lists); h T-cell treatments (358 lists; *transformed cell line rather than primary T cells); i dendritic cell treatments (360 lists); j macrophage treatments (337 lists), and (K) human skin diseases (123 lists; *comparison to uninvolved skin from patients rather than normal skin from independent controls). All lists except (f) were generated from experiments using human cells or tissues. For (f), lists of mouse genes were converted to human genes based upon a mapping of human-mouse orthologues. This additional file includes a description of each list along with GEO series accessions, GEO platform identifiers, and Pubmed IDs (PMIDs) of any associated publications. (XLSX 113 kb) Additional file 4: 
**Genes associated with LC-MS/MS-detected proteins have FPKM values 10–17 times greater than genes not associated with LC-MS/MS-detected proteins.** FPKM values for each sample were compared between genes associated with detected proteins (≥1 SpC) and all other protein-coding genes not associated with a detected protein. Boxes outline the middle 50 % of FPKM values among genes belonging to each group (i.e., 25th–75th percentile). The ratio between median FPKM values for genes belonging to each group is listed (right margin). (TIFF 306 kb) Additional file 5: 
**Correlation between mRNA and protein abundance as measured by RNA-seq (FPKM) and LC-MS/MS (NSAF). We identified 2172 mRNA–protein pairs for which the mRNA was detected by RNA-seq and the protein was detected by LC-MS/MS.** For each mRNA, average FPKM was calculated across the 14 patients in PP and PN skin, respectively. Likewise, for each protein, average NSAF was calculated across the 14 patients in PP and PN skin, respectively. Scatterplots compare average FPKM and NSAF values in PP (a) and PN (b) skin. The *dashed red line* is a least-square regression estimate and the *yellow ellipse* outlines the middle 50 % of data points nearest to the bivariate mean (Mahalanobis distance). c mRNAs most highly expressed in PP and PN skin (FPKM). mRNAs were ranked and selected based upon the larger of the two average FPKM values calculated for PP and PN skin, respectively. d Proteins most highly expressed in PP and PN skin (NSAF). Proteins were ranked and selected based upon the larger of the two average NSAF values calculated for PP and PN skin, respectively. (TIFF 1109 kb) Additional file 6: 
**Association between mRNA (FPKM) and protein (NSAF) abundance for each patient sample.** Scatterplots show the correlation between FPKM and NSAF values with respect to each individual patient sample (PP and PN). *Dashed red lines* represent the least-squares regression estimate and *yellow ellipses* encompass the 50 % of proteins nearest to the bivariate median (Mahalanobis distance). (PDF 1055 kb) Additional file 7: 
**Differentially expressed proteins previously identified by Carlén et al.** [19]. Carlén et al. identified eight proteins with significantly increased abundance in psoriasis lesions. a The eight proteins are not significantly enriched among the PP-increased proteins identified in our analysis (*p* = 0.63). Proteins we detected by LC-MS/MS were ranked in descending order according to the estimated PP/PN fold-change (*horizontal axis*; *red*, PP-increased; *blue*, PP-decreased). The cumulative overlap between the eight proteins and this ranked protein list is shown. *Yellow hash marks* (*top*) denote placement of the eight proteins relative to the ranked protein list from our analysis. b Association between SpC values in PP and PN skin for the eight proteins (*yellow symbols* indicate DEPs). c mRNA (RNA-seq) and protein (LC-MS/MS) fold change estimates. Average FPKM or NSAF values are listed at the base of each bar. Average values were calculated for PP and PN samples, respectively, and the higher of the two values is listed. d Carlén et al. identified KRT15 as decreased in PP skin. The bar graph shows the average normalized SpC value with respect to PP and PN skin samples (±1 standard error). (TIFF 1061 kb) Additional file 8: 
**Differentially expressed proteins previously identified by Ryu et al.** [20]. Ryu et al. identified 36 proteins with significantly increased abundance in psoriasis lesions compared with normal skin. a The 36 proteins significantly enriched among the PP-increased proteins identified in our analysis (*p* = 0.033). Proteins we detected by LC-MS/MS were ranked in descending order according to the estimated PP/PN fold-change (*horizontal axis*; *red*, PP-increased; *blue*, PP-decreased). The cumulative overlap between the 36 proteins and this ranked protein list is shown. *Yellow hash marks* (*top*) denote placement of the 36 proteins relative to the ranked protein list from our analysis. b Association between SpC values in PP and PN skin for the 36 proteins (*yellow symbols* indicate DEPs). c LC-MS/MS-estimated fold changes for each of the 36 proteins (*n* = 14 patients; *yellow bars* indicate DEPs). d Twelve of the 36 proteins showing the strongest mRNA and protein increase. e Twelve of the 36 proteins showing the strongest mRNA and protein decrease. (TIFF 1598 kb) Additional file 9: 
**Shifts in protein abundance in psoriasis are not associated with molecular weight, despite gene length bias affecting mRNA differential expression.** a Gene length bias affecting differential mRNA expression in psoriasis (15,616 skin-expressed genes). The figure shows the percentage of DEGs among genes within different bins varying by gene length (*red*, percentage of PP-increased DEGs; *blue*, percentage of PP-decreased DEGs). The percentage of PP-increased and PP-decreased DEGs is listed within each bar (*yellow*: *p* < 0.05, Fisher’s exact test). *Asterisks* above bars indicate significant over-abundance of DEGs (PP-increased plus PP-decreased) with respect to a given gene bin (FDR < 0.05, Fisher’s exact test). b Median gene length among PP-decreased DEGs is greater than among PP-increased DEGs. Boxes outline the middle 50 % of gene lengths in each DEG group (25th percentile, median and 75th percentile; whiskers span the 10th to 90th percentile). The median gene length is listed for each group along with the number of DEGs, with *p* value generated from the comparison of gene lengths between groups (Wilcoxon rank sum test). Parts (c) and (d) are the same as (a) and (b), respectively, except only 2088 genes associated with detected proteins are analyzed. Similarly, parts (e) and (f) are the same as (a) and (b), respectively, except 2194 proteins are analyzed to assess whether the percentage of PP-increased and PP-decreased DEPs differs according to protein molecular weight (kDa). (TIFF 1532 kb) Additional file 10: 
**Association between mRNA (RNA-seq) and protein (LC-MS/MS) fold changes (PP/PN) with respect to individual patients.** Fold changes generated by RNA-seq and LC-MS/MS were compared for each individual patient (2087 mRNA–protein pairs). *Dashed red lines* represent least-square regression estimates and *yellow ellipses* encompass 50 % of proteins nearest to the bivariate mean (Mahalanobis distance). (PDF 459 kb) Additional file 11: 
**The association between mRNA (RNA-seq) and protein (LC-MS/MS) fold changes (PP/PN) does not differ between low- and high-expressed genes.** The 2087 mRNA–protein pairs were divided into four groups based upon average FPKM. Average FPKM was calculated with respect to PP and PN samples, respectively, and the higher of the two values was used to assign mRNA–protein pairs to each group (approximately 520 pairs per group). The figure shows the association between RNA-seq- and LC-MS/MS-estimated fold changes for mRNAs with average FPKM values beneath the 25th percentile (a), within the 25–50th percentile (b), within the 50–75th percentile (c), and above the 75th percentile (d). *Dashed red lines* represent least-square regression estimates and *yellow ellipses* encompass the 50 % of proteins nearest to the bivariate mean (Mahalanobis distance). (TIFF 185 kb) Additional file 12: 
**Decreased abundance of ribosomal subunit mRNA and protein in psoriasis lesions.** a–c RT-PCR was used to measure expression of three genes in lesional skin from psoriasis patients (PP), uninvolved skin from psoriasis patients (PN), and normal skin from healthy control subjects (NN) (*n* = 8 per group; ±1 standard error). Gene expression for each sample is normalized to the expression of glyceraldehyde-3-phosphate dehydrogenase (*GAPDH*). Fold-changes from *p* values are listed for the comparison between PP and PN groups (*top*; paired Wilcoxon rank sum test) and between PP and NN groups (*bottom*; Wilcoxon rank sum test). b Western blot analysis of RPL7A, RPS8, EEF1A1, RPS3A and RPL11 in PP, PN and NN skin (*n* = 3 per group). c Immunohistochemical staining of RPL7A in lesional psoriasis skin. (TIFF 3429 kb) Additional file 13: 
**Proteins expressed in lymphoid organs are elevated in psoriasis lesions (ProteomicsDB).** a ProteomicsDB cell types ranked by the correlation between cell type-specific expression and LC-MS/MS-estimated fold change (PP/PN; 2198 proteins). b Association between cell type-specific expression and fold change (PP/PN) for lymph node. c ProteomicsDB cell types ranked according to how strongly the 12 best signature proteins for each cell type are enriched among those proteins with elevated abundance in PP skin. d Enrichment of spleen signature proteins among PP-increased proteins. e Spleen signature proteins and their relative abundance across ProteomicsDB cell types. See Fig. 4 legend for further details. (TIFF 1865 kb) Additional file 14: 
**Proteins expressed in transformed cells are elevated in psoriasis lesions (CPL/MUW database).** a CPL/MUW database cell types ranked by the correlation between cell type-specific expression and LC-MS/MS-estimated fold change (PP/PN; 1656 proteins). b Association between cell type-specific expression and fold change (PP/PN) for Hep3B cells. c CPL/MUW database cell types ranked according to how strongly the 12 best signature proteins for each cell type are enriched among those proteins with elevated abundance in PP skin. d Enrichment of HepG2 signature proteins among PP-increased proteins. e HepG2 signature proteins and their relative abundance across CPL/MUW cell types. See Fig. 4 legend for further details. (TIFF 1652 kb) Additional file 15: 
**Fatty acid binding protein 5 (FABP5) and serpin peptidase inhibitor 4 (SERPINB4) show correspondent increases in mRNA and protein abundance in psoriasis lesions.** (A) Expression of *FABP5* in lesional (PP), uninvolved (PN) and normal skin from healthy controls (NN) (RT-PCR; *n* = 8 per group). (B) FABP5 immunohistochemistry staining in PP skin. (C) FABP5 immunohistochemistry staining in PN skin. (D) Expression of *SERPINB4* in lesional (PP), uninvolved (PN) and normal skin from healthy controls (NN) (RT-PCR; *n* = 8 per group). For RT-PCR analyses (A and D), two sets of fold-changes and *p* values are listed (top: PP versus PN; bottom: PP versus NN). Gene expression for each sample is normalized to glyceraldehyde-3-phosphate dehydrogenase (*GAPDH*). (TIFF 4624 kb) Additional file 16: 
**GO biological process terms enriched among PP-increased DEGPs and PP-decreased DEGPs.** a Top-ranked GO biological process (BP) terms enriched among the 153 PP-increased DEGPs. b Top-ranked GO BP terms enriched among the 56 PP-decreased DEGPs. In both (a) and (b), enrichment was evaluated with respect to a background set of 2087 genes associated with mRNAs and proteins detected using RNA-seq and LC-MS/MS, respectively. The right margin lists example DEGPs associated with each GO BP term. (TIFF 1028 kb) Additional file 17: 
**DEGP enrichment with respect to ordered gene lists from microarray experiments.** We screened 2178 ordered gene lists to identify microarray experiments in which PP-increased and PP-decreased DEGPs were disproportionately increased or decreased but in opposite directions. The top-ranked 20 experiments are listed for each of 11 experiment types (a–k; see Additional file 3). Each figure (a–k) shows enrichment statistics for both the 153 PP-increased (*left panel*) and 56 PP-decreased DEGPs (*right panel*) with respect to each ordered gene list (described in left margin). Positive enrichment statistics indicate that DEGPs are elevated in the experiment relative to control samples. Negative enrichment statistics indicate that DEGPs are repressed in the experiment relative to control samples. *P* values are listed in the right margin (Wilcoxon rank sum test). For each experiment, *p* values were calculated for PP-increased DEGPs and PP-decreased DEGPs, respectively, and experiments are ranked according to the higher of these two *p* values. (PDF 6387 kb) Additional file 18: 
**Psoriasis-specific and non-specific DEGPs show divergent responses in KCs following gene perturbations and in mouse skin phenotypes.** Analyses shown in Figs. 6 and 7 were repeated with respect to KC gene perturbations (a) (65 lists; *HaCaT; **reconstituted epidermis; “ + ” denotes overexpression of a gene rather than RNA interference knockdown) and laboratory mouse phenotypes (b) (35 lists). For each ordered gene list set, the sliding window approach was used to identify the ten lists with the strongest enrichment differences between psoriasis-specific DEGPs and non-specific DEGPs (see Figs. 6 and 7 legends). (TIFF 2368 kb)

## Abbreviations

2D: two-dimensional
bp: base pair
DEG: differentially expressed gene
DEGP: differentially expressed gene/protein
DEP: differentially expressed protein
FDR: false discovery rate
FPKM: fragments per kilobase of exon per million fragments mapped
GEO: Gene Expression Omnibus
GO: gene ontology
GSEA: gene set enrichment analysis
IFN: interferon
IL: interleukin
KC: keratinocyte
LC-MS/MS: liquid chromatography-tandem mass spectrometry
MS: mass spectrometry
MS/MS: tandem mass spectrometry
mTOR: mammalian target of rapamycin
NN: normal skin from control subject
NSAF: normalized spectral abundance factor
PN: uninvolved normal skin from psoriasis patient
PP: lesional skin from psoriasis patient
SpC: spectral count
TNF: tumor necrosis factor
UCSC: University of California, Santa Cruz
