# Supplementary material for: Proteogenomic analysis of psoriasis reveals discordant and concordant changes in mRNA and protein abundance
Source: Genome Med. 2015 Aug 4;7(1):86. doi: 10.1186/s13073-015-0208-5 (PMC4527112; doi:10.1186/s13073-015-0208-5)
Supplement: Additional file 1: — Clinical characteristics of psoriasis patients providing lesional and uninvolved biopsies for RNA-seq and LC-MS/MS analysis ( n = 14). The table lists sex, age range, body mass index (BMI), body weight (kg), psoriasis total body surface area (TBSA) and psoriasis area severity index (PASI). For some patients, information was absent from clinical records and is thus not included in the table (“unknown” entries). (PDF 120 kb) [file 13073_2015_208_MOESM1_ESM.pdf]

Additional File 1. Clinical characteristics of psoriasis patients providing lesional and uninvolved biopsies for RNA-seq and LC-MS/MS analysis (*n* = 14). The table lists sex, age range, body mass index (BMI), body weight (kg), psoriasis total body surface area (TBSA) and psoriasis area severity index (PASI). For some patients, information was absent from clinical records and is thus not included in the table ("unknown" entries).

Patient ID	Sex	Age Range	BMI	Weight (kg)	TBSA*	PASI*
1	male	40-49	unknown	unknown	unknown	unknown
2	male	40-49	unknown	unknown	24	12.6
3	male	50-59	28.3	99.8	12.5	16.53
4	female	40-49	43.0	103.0	4.2	7.6
5	male	60-69	36.6	108.9	42	16.8
6	female	50-60	unknown	unknown	21	6.6
7	male	60-69	35.0	108.4	15	5.1
8	male	50-59	unknown	unknown	38	19.1
9	male	70-79	29.3	95.3	28	21.6
10	male	40-49	25.3	66.7	6	6.7
11	female	50-59	42.6	108.9	25	10.5
12	female	30-39	21.5	49.9	4.4	9.6
13	female	40-49	43.0	103.0	4.2	7.6
14	female	20-29	25.1	70.3	2.5	3.8

*Estimated by physical examination or self-evaluation at the time biopsies were collected
