# Supplementary material for: Proteogenomic analysis of psoriasis reveals discordant and concordant changes in mRNA and protein abundance
Source: Genome Med. 2015 Aug 4;7(1):86. doi: 10.1186/s13073-015-0208-5 (PMC4527112; doi:10.1186/s13073-015-0208-5)
Supplement: Additional file 2: — RNA-seq quality control. RNA-seq was used to analyze 28 skin samples from 14 psoriasis patients (PP and PN skin). a Number of reads prior to filtering. b Number of reads after filtering. c Percentage of reads mapped to the UCSC human genome (hg19). d Percentage of reads mapping to intragenic regions. e Mean read coverage per genomic base. f Cluster analysis of the 28 samples. The dendrogram was generated using average linkage hierarchical clustering. Euclidean distance between samples was estimated based upon FPKM for the 15,616 protein-coding mRNAs detected in at least 25 % of samples (i.e., at least 7 of 28 samples). g Two dimensional principal component plot. The 28 samples were plotted with respect to the first two principal component axes. Principal component axes were calculated using FPKM values for the 15,615 protein-coding mRNAs. h Expression patterns of known psoriasis DEGs. PP-increased (horizontal axis) and PP-decreased DEG scores (vertical axis) were calculated for each patient, based upon DEGs identified from an earlier meta-analysis of microarray data (n = 237 patients). The PP-increased score (horizontal axis) is equal to the average fold-change (PP/PN) of the 100 DEGs most strongly elevated in the meta-analysis. The PP-decreased score (horizontal axis) is equal to the average fold change (PP/PN) of the 100 DEGs most strongly repressed in the meta-analysis. (PDF 3507 kb) [file 13073_2015_208_MOESM2_ESM.pdf]

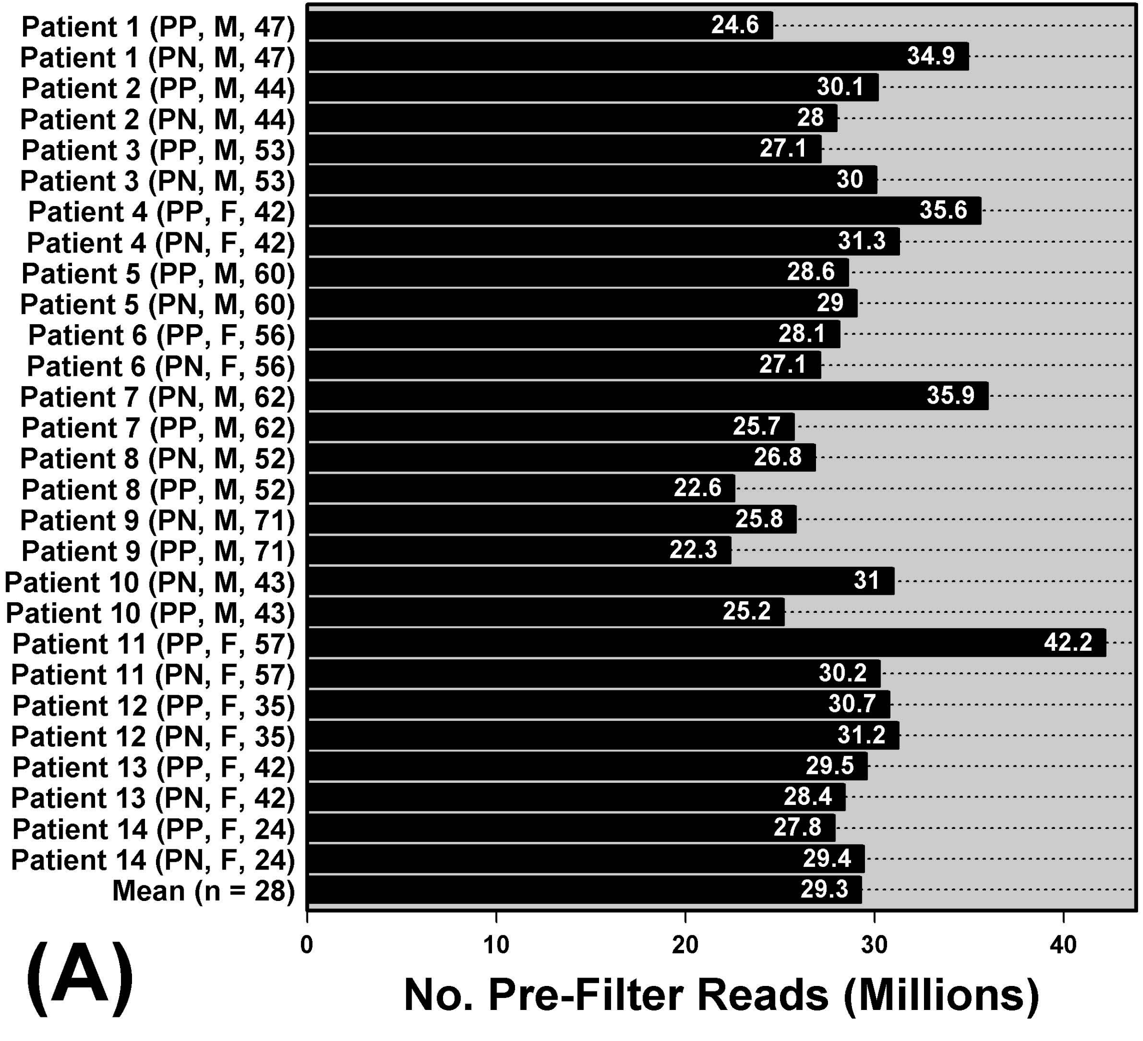
Patient 1 (PP, M, 47)
Patient 1 (PN, M, 47)
Patient 2 (PP, M, 44)
Patient 2 (PN, M, 44)
Patient 3 (PP, M, 53)
Patient 3 (PN, M, 53)
Patient 4 (PP, F, 42)
Patient 4 (PN, F, 42)
Patient 5 (PP, M, 60)
Patient 5 (PN, M, 60)
Patient 6 (PP, F, 56)
Patient 6 (PN, F, 56)
Patient 7 (PN, M, 62)
Patient 7 (PP, M, 62)
Patient 8 (PN, M, 52)
Patient 8 (PP, M, 52)
Patient 9 (PN, M, 71)
Patient 9 (PP, M, 71)
Patient 10 (PN, M, 43)
Patient 10 (PP, M, 43)
Patient 11 (PP, F, 57)
Patient 11 (PN, F, 57)
Patient 12 (PP, F, 35)
Patient 12 (PN, F, 35)
Patient 13 (PP, F, 42)
Patient 13 (PN, F, 42)
Patient 14 (PP, F, 24)
Patient 14 (PN, F, 24)
Mean (n = 28)
24.6
34.9
30.1
28
27.1
30
35.6
31.3
28.6
29
28.1
27.1
35.9
25.7
26.8
22.6
25.8
22.3
31
25.2
42.2
30.2
30.7
31.2
29.5
28.4
27.8
29.4
29.3
0
10
20
30
40
No. Pre-Filter Reads (Millions)
(A)

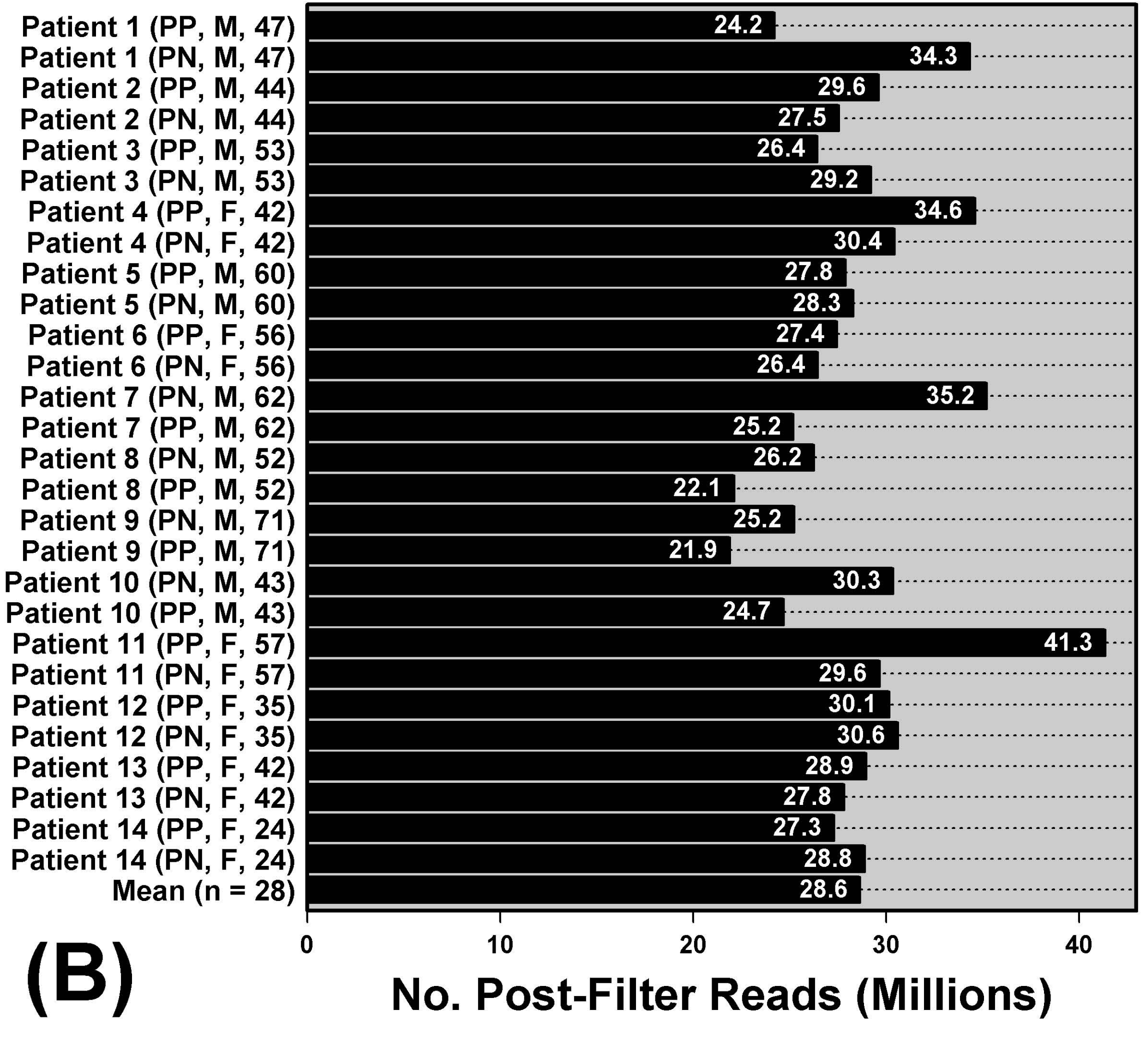

Patient 1 (PP, M, 47)
Patient 1 (PN, M, 47)
Patient 2 (PP, M, 44)
Patient 2 (PN, M, 44)
Patient 3 (PP, M, 53)
Patient 3 (PN, M, 53)
Patient 4 (PP, F, 42)
Patient 4 (PN, F, 42)
Patient 5 (PP, M, 60)
Patient 5 (PN, M, 60)
Patient 6 (PP, F, 56)
Patient 6 (PN, F, 56)
Patient 7 (PN, M, 62)
Patient 7 (PP, M, 62)
Patient 8 (PN, M, 52)
Patient 8 (PP, M, 52)
Patient 9 (PN, M, 71)
Patient 9 (PP, M, 71)
Patient 10 (PN, M, 43)
Patient 10 (PP, M, 43)
Patient 11 (PP, F, 57)
Patient 11 (PN, F, 57)
Patient 12 (PP, F, 35)
Patient 12 (PN, F, 35)
Patient 13 (PP, F, 42)
Patient 13 (PN, F, 42)
Patient 14 (PP, F, 24)
Patient 14 (PN, F, 24)
Mean (n = 28)
24.2
34.3
29.6
27.5
26.4
29.2
34.6
30.4
27.8
28.3
27.4
26.4
35.2
25.2
26.2
22.1
25.2
21.9
30.3
24.7
41.3
29.6
30.1
30.6
28.9
27.8
27.3
28.8
28.6
0
10
20
30
40
(B)
No. Post-Filter Reads (Millions)

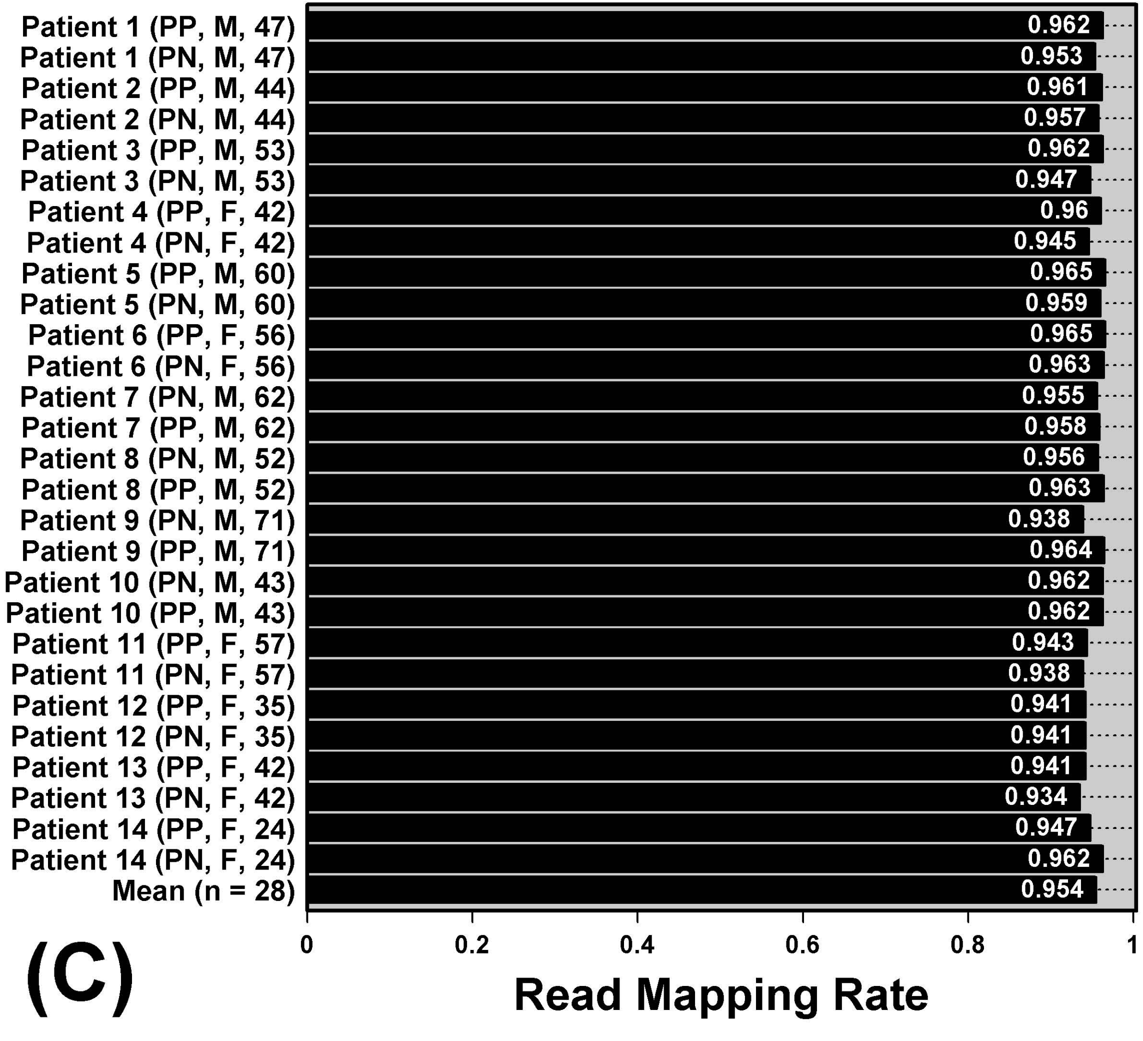
Patient 1 (PP, M, 47)
0.962
Patient 1 (PN, M, 47)
0.953
Patient 2 (PP, M, 44)
0.961
Patient 2 (PN, M, 44)
0.957
Patient 3 (PP, M, 53)
0.962
Patient 3 (PN, M, 53)
0.947
Patient 4 (PP, F, 42)
0.96
Patient 4 (PN, F, 42)
0.945
Patient 5 (PP, M, 60)
0.965
Patient 5 (PN, M, 60)
0.959
Patient 6 (PP, F, 56)
0.965
Patient 6 (PN, F, 56)
0.963
Patient 7 (PN, M, 62)
0.955
Patient 7 (PP, M, 62)
0.958
Patient 8 (PN, M, 52)
0.956
Patient 8 (PP, M, 52)
0.963
Patient 9 (PN, M, 71)
0.938
Patient 9 (PP, M, 71)
0.964
Patient 10 (PN, M, 43)
0.962
Patient 10 (PP, M, 43)
0.962
Patient 11 (PP, F, 57)
0.943
Patient 11 (PN, F, 57)
0.938
Patient 12 (PP, F, 35)
0.941
Patient 12 (PN, F, 35)
0.941
Patient 13 (PP, F, 42)
0.941
Patient 13 (PN, F, 42)
0.934
Patient 14 (PP, F, 24)
0.947
Patient 14 (PN, F, 24)
0.962
Mean (n = 28)
0.954
0
0.2
0.4
0.6
0.8
1
(C)
Read Mapping Rate

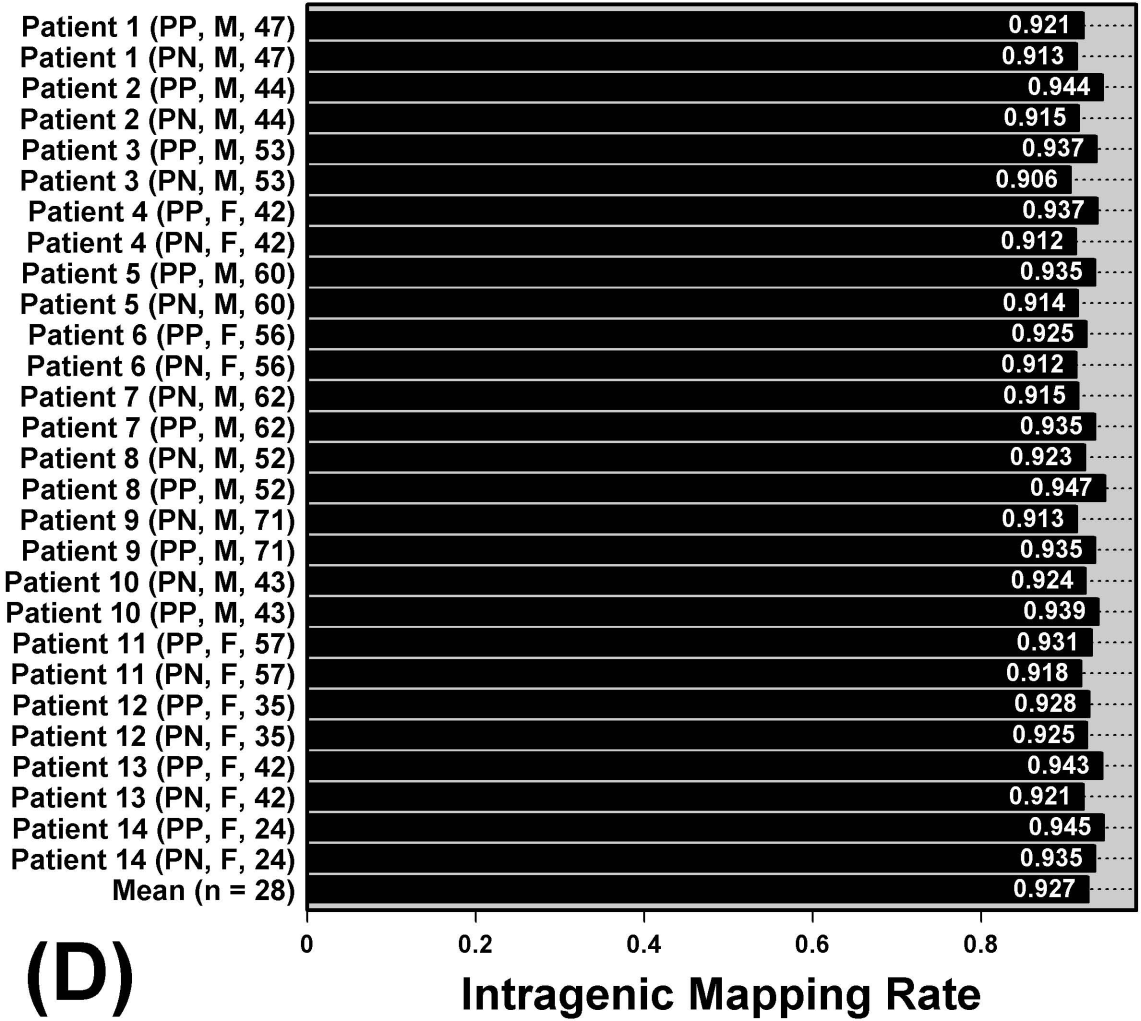
Patient 1 (PP, M, 47)
Patient 1 (PN, M, 47)
Patient 2 (PP, M, 44)
Patient 2 (PN, M, 44)
Patient 3 (PP, M, 53)
Patient 3 (PN, M, 53)
Patient 4 (PP, F, 42)
Patient 4 (PN, F, 42)
Patient 5 (PP, M, 60)
Patient 5 (PN, M, 60)
Patient 6 (PP, F, 56)
Patient 6 (PN, F, 56)
Patient 7 (PN, M, 62)
Patient 7 (PP, M, 62)
Patient 8 (PN, M, 52)
Patient 8 (PP, M, 52)
Patient 9 (PN, M, 71)
Patient 9 (PP, M, 71)
Patient 10 (PN, M, 43)
Patient 10 (PP, M, 43)
Patient 11 (PP, F, 57)
Patient 11 (PN, F, 57)
Patient 12 (PP, F, 35)
Patient 12 (PN, F, 35)
Patient 13 (PP, F, 42)
Patient 13 (PN, F, 42)
Patient 14 (PP, F, 24)
Patient 14 (PN, F, 24)
Mean (n = 28)
0.921
0.913
0.944
0.915
0.937
0.906
0.937
0.912
0.935
0.914
0.925
0.912
0.915
0.935
0.923
0.947
0.913
0.935
0.924
0.939
0.931
0.918
0.928
0.925
0.943
0.921
0.945
0.935
0.927
0
0.2
0.4
0.6
0.8
Intragenic Mapping Rate

(D)

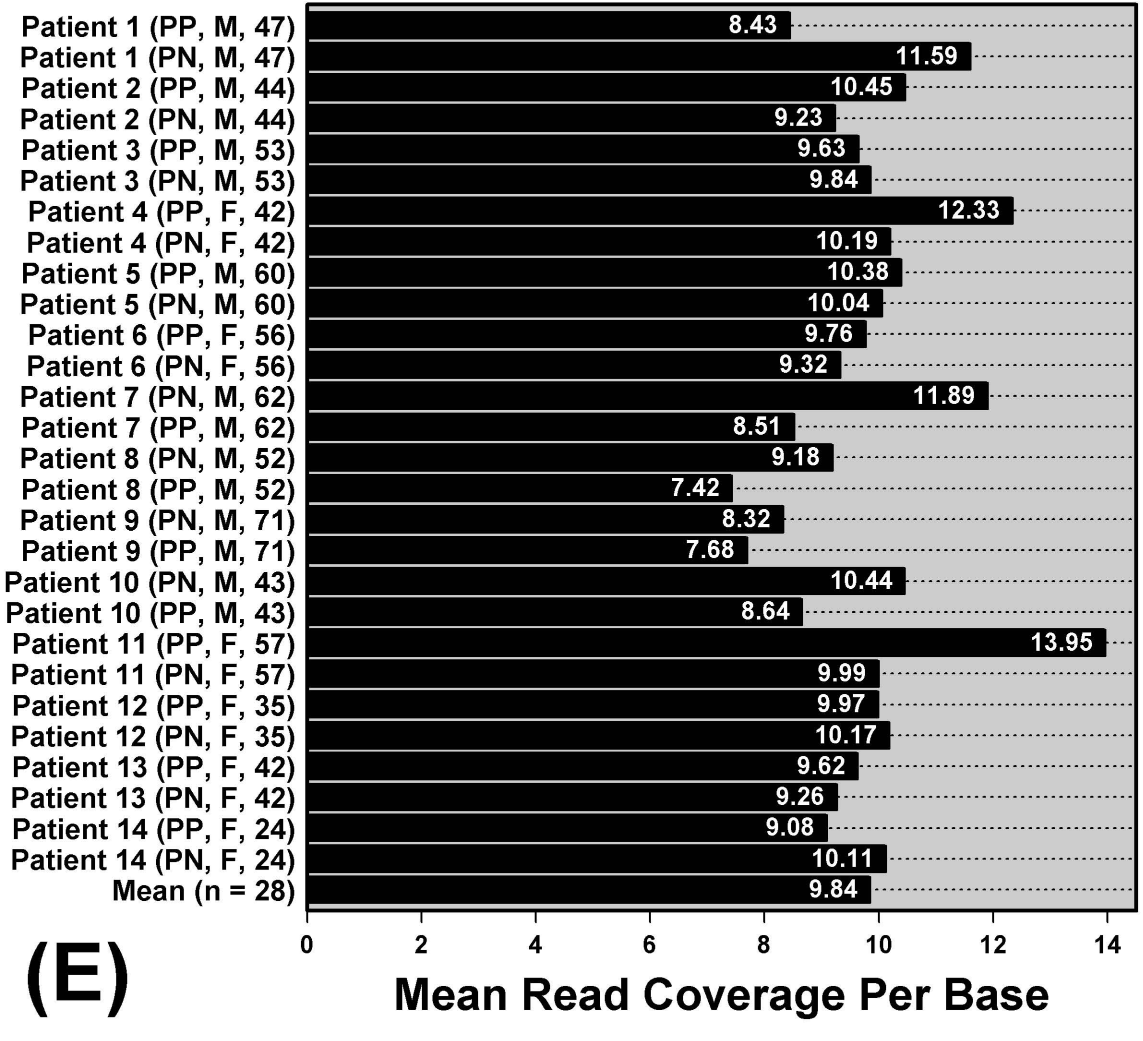
Patient 1 (PP, M, 47)
Patient 1 (PN, M, 47)
Patient 2 (PP, M, 44)
Patient 2 (PN, M, 44)
Patient 3 (PP, M, 53)
Patient 3 (PN, M, 53)
Patient 4 (PP, F, 42)
Patient 4 (PN, F, 42)
Patient 5 (PP, M, 60)
Patient 5 (PN, M, 60)
Patient 6 (PP, F, 56)
Patient 6 (PN, F, 56)
Patient 7 (PN, M, 62)
Patient 7 (PP, M, 62)
Patient 8 (PN, M, 52)
Patient 8 (PP, M, 52)
Patient 9 (PN, M, 71)
Patient 9 (PP, M, 71)
Patient 10 (PN, M, 43)
Patient 10 (PP, M, 43)
Patient 11 (PP, F, 57)
Patient 11 (PN, F, 57)
Patient 12 (PP, F, 35)
Patient 12 (PN, F, 35)
Patient 13 (PP, F, 42)
Patient 13 (PN, F, 42)
Patient 14 (PP, F, 24)
Patient 14 (PN, F, 24)
Mean (n = 28)
8.43
11.59
10.45
9.23
9.63
9.84
12.33
10.19
10.38
10.04
9.76
9.32
11.89
8.51
9.18
7.42
8.32
7.68
10.44
8.64
13.95
9.99
9.97
10.17
9.62
9.26
9.08
10.11
9.84
0
2
4
6
8
10
12
14
Mean Read Coverage Per Base
(E)

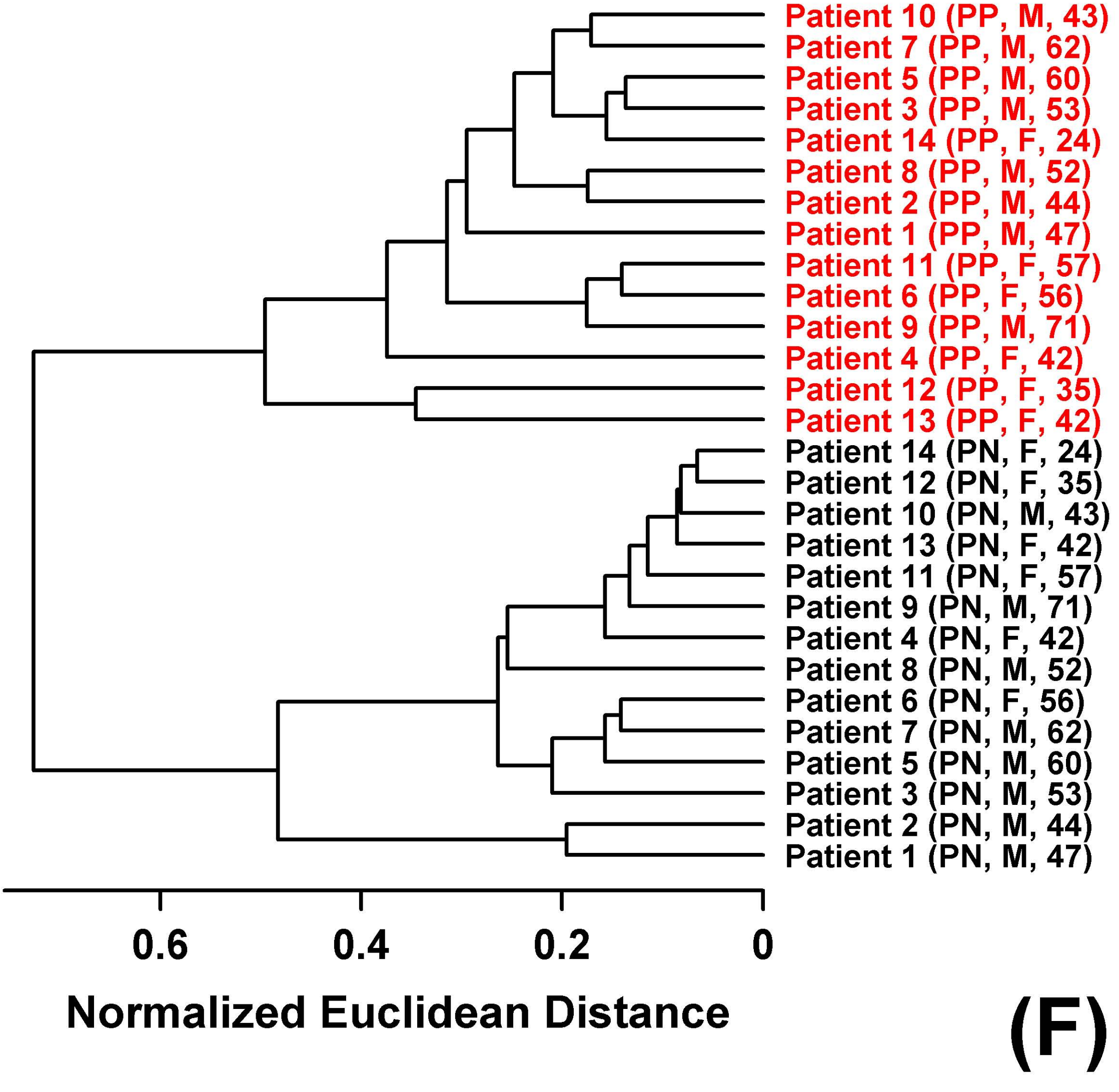
Patient 10 (PP, M, 43)
Patient 7 (PP, M, 62)
Patient 5 (PP, M, 60)
Patient 3 (PP, M, 53)
Patient 14 (PP, F, 24)
Patient 8 (PP, M, 52)
Patient 2 (PP, M, 44)
Patient 1 (PP, M, 47)
Patient 11 (PP, F, 57)
Patient 6 (PP, F, 56)
Patient 9 (PP, M, 71)
Patient 4 (PP, F, 42)
Patient 12 (PP, F, 35)
Patient 13 (PP, F, 42)
Patient 14 (PN, F, 24)
Patient 12 (PN, F, 35)
Patient 10 (PN, M, 43)
Patient 13 (PN, F, 42)
Patient 11 (PN, F, 57)
Patient 9 (PN, M, 71)
Patient 4 (PN, F, 42)
Patient 8 (PN, M, 52)
Patient 6 (PN, F, 56)
Patient 7 (PN, M, 62)
Patient 5 (PN, M, 60)
Patient 3 (PN, M, 53)
Patient 2 (PN, M, 44)
Patient 1 (PN, M, 47)
0.6
0.4
0.2
0
Normalized Euclidean Distance
(F)

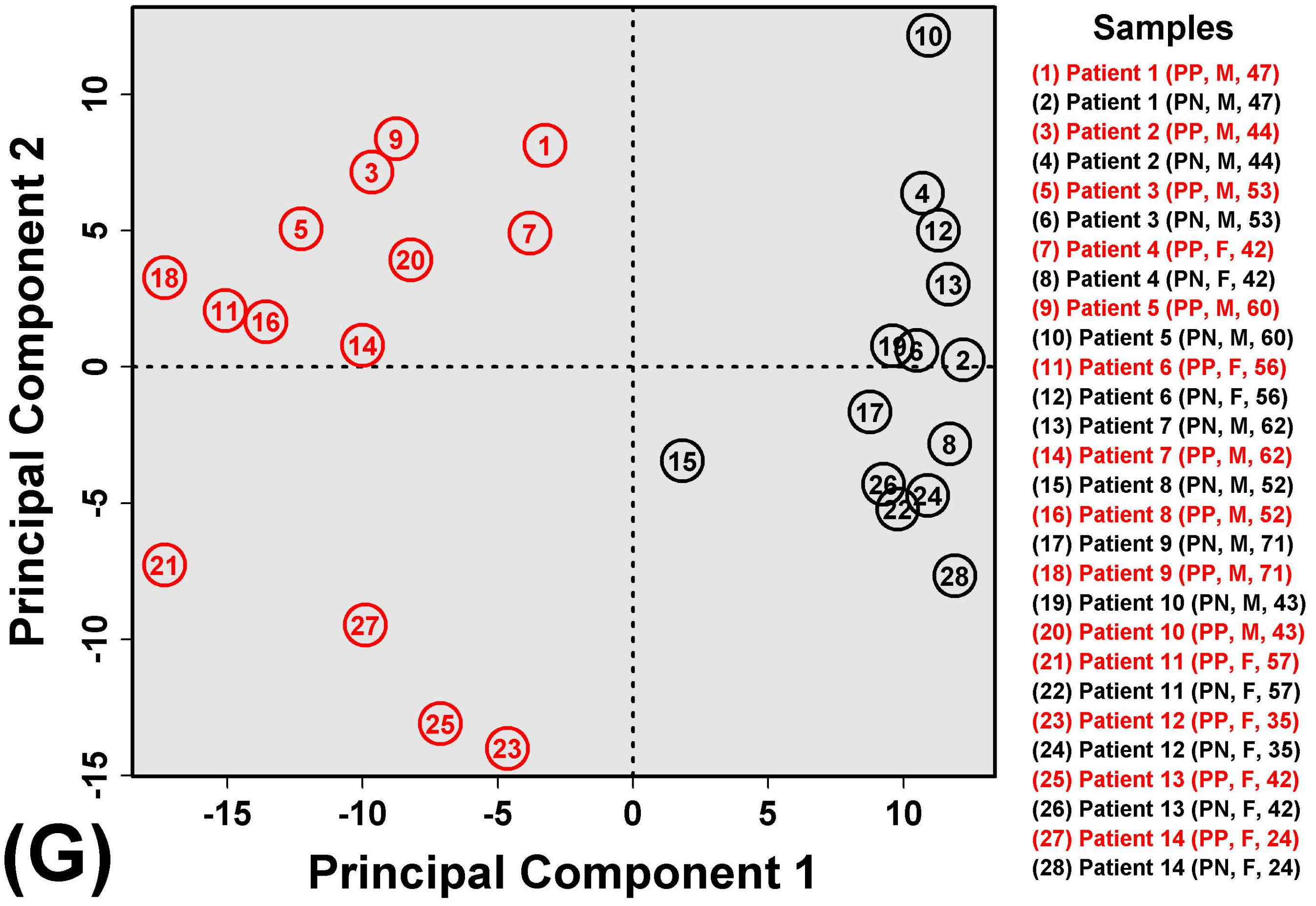
Samples
(1) Patient 1 (PP, M, 47)
(2) Patient 1 (PN, M, 47)
(3) Patient 2 (PP, M, 44)
(4) Patient 2 (PN, M, 44)
(5) Patient 3 (PP, M, 53)
(6) Patient 3 (PN, M, 53)
(7) Patient 4 (PP, F, 42)
(8) Patient 4 (PN, F, 42)
(9) Patient 5 (PP, M, 60)
(10) Patient 5 (PN, M, 60)
(11) Patient 6 (PP, F, 56)
(12) Patient 6 (PN, F, 56)
(13) Patient 7 (PN, M, 62)
(14) Patient 7 (PP, M, 62)
(15) Patient 8 (PN, M, 52)
(16) Patient 8 (PP, M, 52)
(17) Patient 9 (PN, M, 71)
(18) Patient 9 (PP, M, 71)
(19) Patient 10 (PN, M, 43)
(20) Patient 10 (PP, M, 43)
(21) Patient 11 (PP, F, 57)
(22) Patient 11 (PN, F, 57)
(23) Patient 12 (PP, F, 35)
(24) Patient 12 (PN, F, 35)
(25) Patient 13 (PP, F, 42)
(26) Patient 13 (PN, F, 42)
(27) Patient 14 (PP, F, 24)
(28) Patient 14 (PN, F, 24)
Principal Component 1
Principal Component 2
-15
-10
-5
0
5
10

(G)

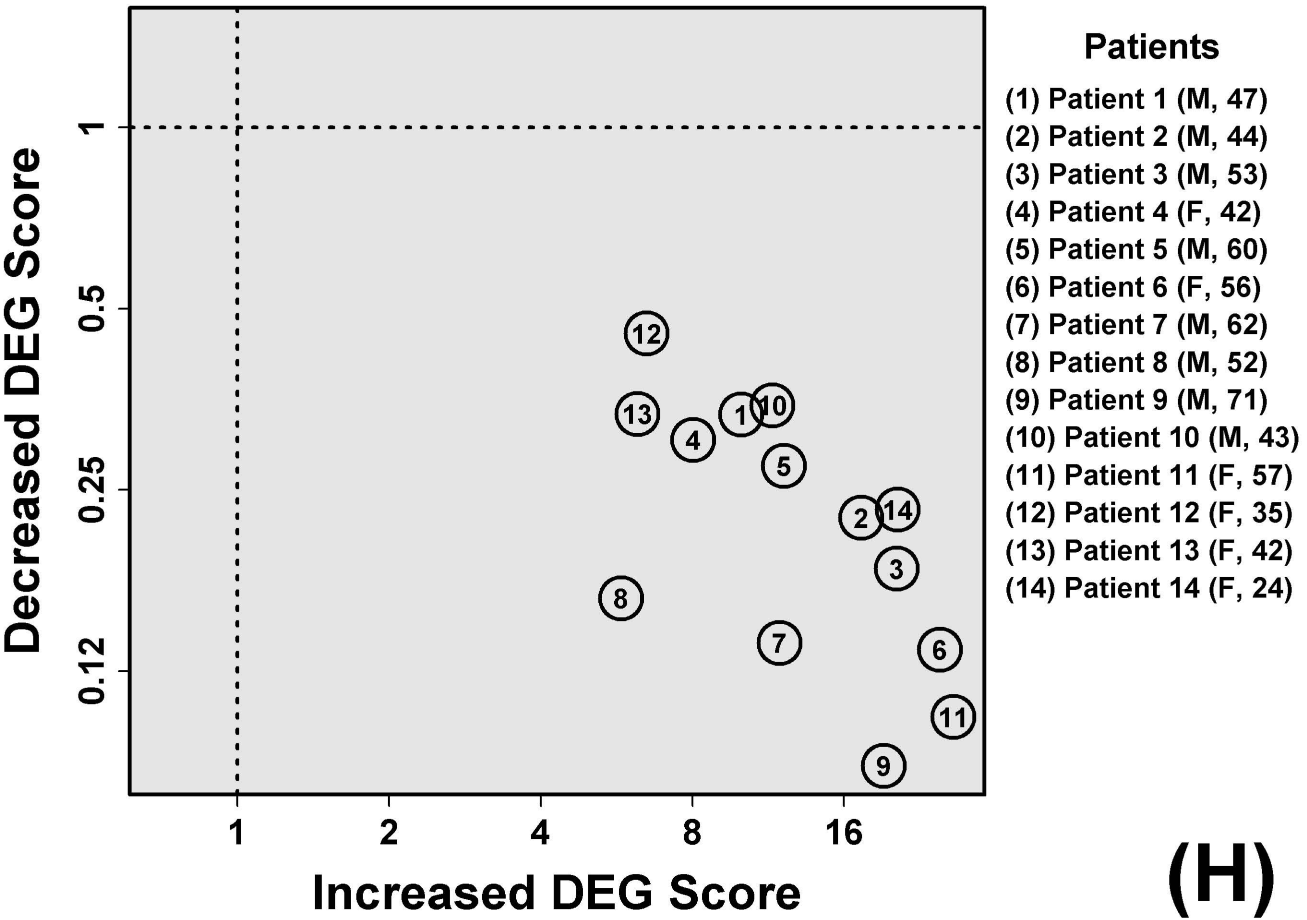
Patients
(1) Patient 1 (M, 47)
(2) Patient 2 (M, 44)
(3) Patient 3 (M, 53)
(4) Patient 4 (F, 42)
(5) Patient 5 (M, 60)
(6) Patient 6 (F, 56)
(7) Patient 7 (M, 62)
(8) Patient 8 (M, 52)
(9) Patient 9 (M, 71)
(10) Patient 10 (M, 43)
(11) Patient 11 (F, 57)
(12) Patient 12 (F, 35)
(13) Patient 13 (F, 42)
(14) Patient 14 (F, 24)
Decreased DEG Score
1
0.5
0.25
0.12
Increased DEG Score
1
2
4
8
16
(H)
