# Supplementary figures and images for: Proteogenomic analysis of psoriasis reveals discordant and concordant changes in mRNA and protein abundance
Source: Genome Med. 2015 Aug 4;7(1):86. doi: 10.1186/s13073-015-0208-5 (PMC4527112; doi:10.1186/s13073-015-0208-5)

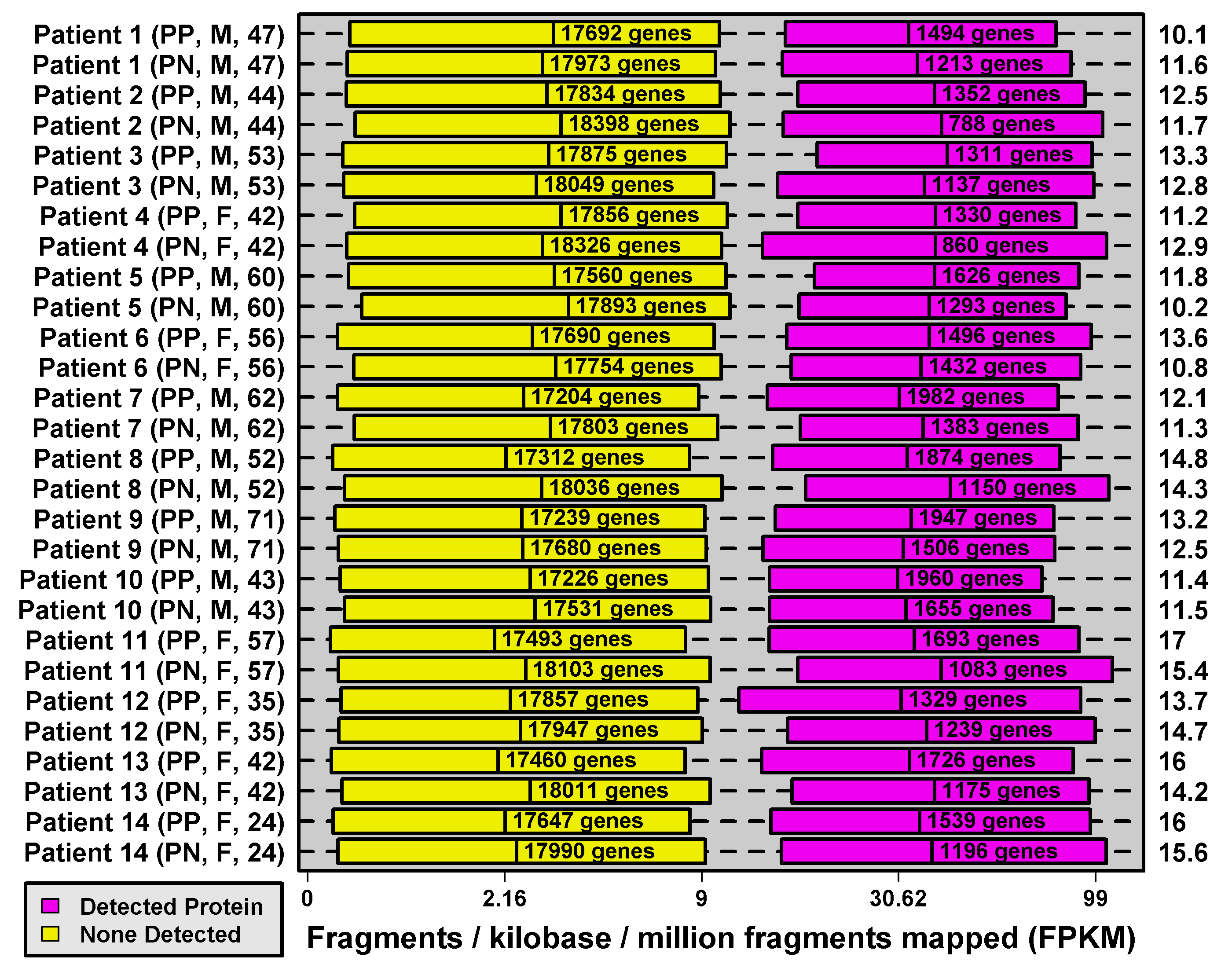

Supplement: Additional file 4: — Genes associated with LC-MS/MS-detected proteins have FPKM values 10–17 times greater than genes not associated with LC-MS/MS-detected proteins. FPKM values for each sample were compared between genes associated with detected proteins (≥1 SpC) and all other protein-coding genes not associated with a detected protein. Boxes outline the middle 50 % of FPKM values among genes belonging to each group (i.e., 25th–75th percentile). The ratio between median FPKM values for genes belonging to each group is listed (right margin). (TIFF 306 kb) [file 13073_2015_208_MOESM4_ESM.tif]

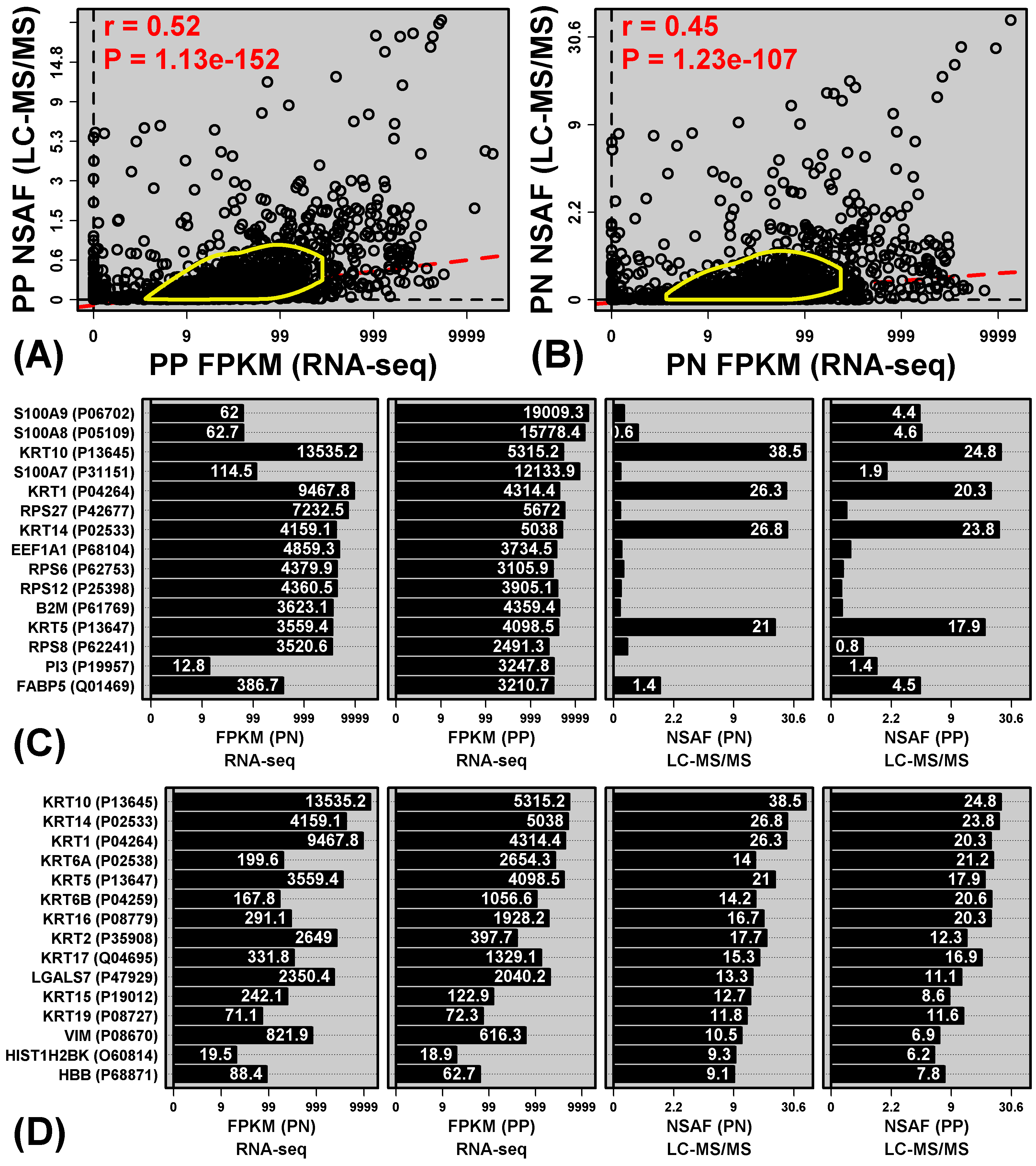

Supplement: Additional file 5: — Correlation between mRNA and protein abundance as measured by RNA-seq (FPKM) and LC-MS/MS (NSAF). We identified 2172 mRNA–protein pairs for which the mRNA was detected by RNA-seq and the protein was detected by LC-MS/MS. For each mRNA, average FPKM was calculated across the 14 patients in PP and PN skin, respectively. Likewise, for each protein, average NSAF was calculated across the 14 patients in PP and PN skin, respectively. Scatterplots compare average FPKM and NSAF values in PP (a) and PN (b) skin. The dashed red line is a least-square regression estimate and the yellow ellipse outlines the middle 50 % of data points nearest to the bivariate mean (Mahalanobis distance). c mRNAs most highly expressed in PP and PN skin (FPKM). mRNAs were ranked and selected based upon the larger of the two average FPKM values calculated for PP and PN skin, respectively. d Proteins most highly expressed in PP and PN skin (NSAF). Proteins were ranked and selected based upon the larger of the two average NSAF values calculated for PP and PN skin, respectively. (TIFF 1109 kb) [file 13073_2015_208_MOESM5_ESM.tif]

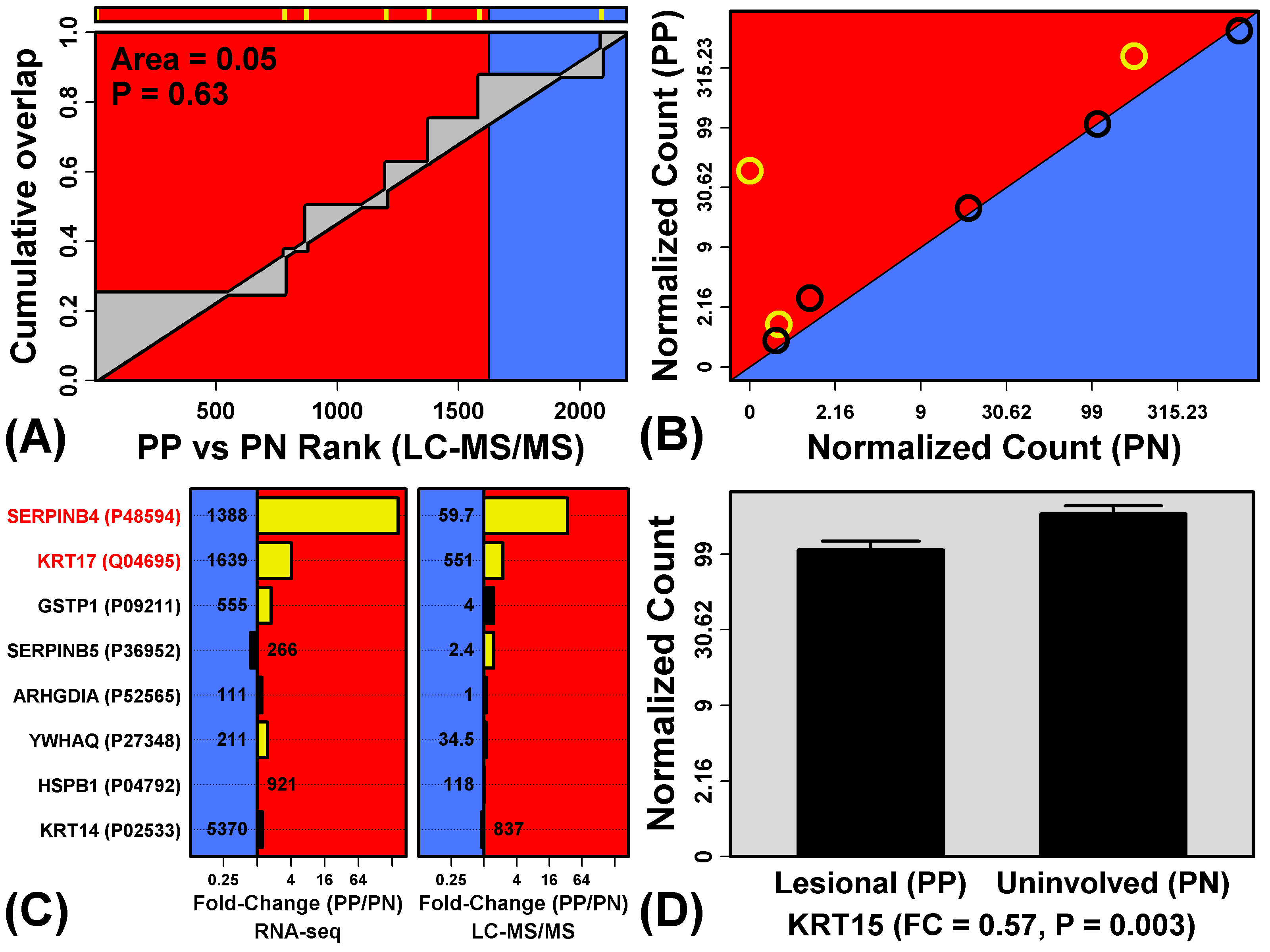

Supplement: Additional file 7: — Differentially expressed proteins previously identified by Carlén et al. [19]. Carlén et al. identified eight proteins with significantly increased abundance in psoriasis lesions. a The eight proteins are not significantly enriched among the PP-increased proteins identified in our analysis (p = 0.63). Proteins we detected by LC-MS/MS were ranked in descending order according to the estimated PP/PN fold-change (horizontal axis; red, PP-increased; blue, PP-decreased). The cumulative overlap between the eight proteins and this ranked protein list is shown. Yellow hash marks (top) denote placement of the eight proteins relative to the ranked protein list from our analysis. b Association between SpC values in PP and PN skin for the eight proteins (yellow symbols indicate DEPs). c mRNA (RNA-seq) and protein (LC-MS/MS) fold change estimates. Average FPKM or NSAF values are listed at the base of each bar. Average values were calculated for PP and PN samples, respectively, and the higher of the two values is listed. d Carlén et al. identified KRT15 as decreased in PP skin. The bar graph shows the average normalized SpC value with respect to PP and PN skin samples (±1 standard error). (TIFF 1061 kb) [file 13073_2015_208_MOESM7_ESM.tif]

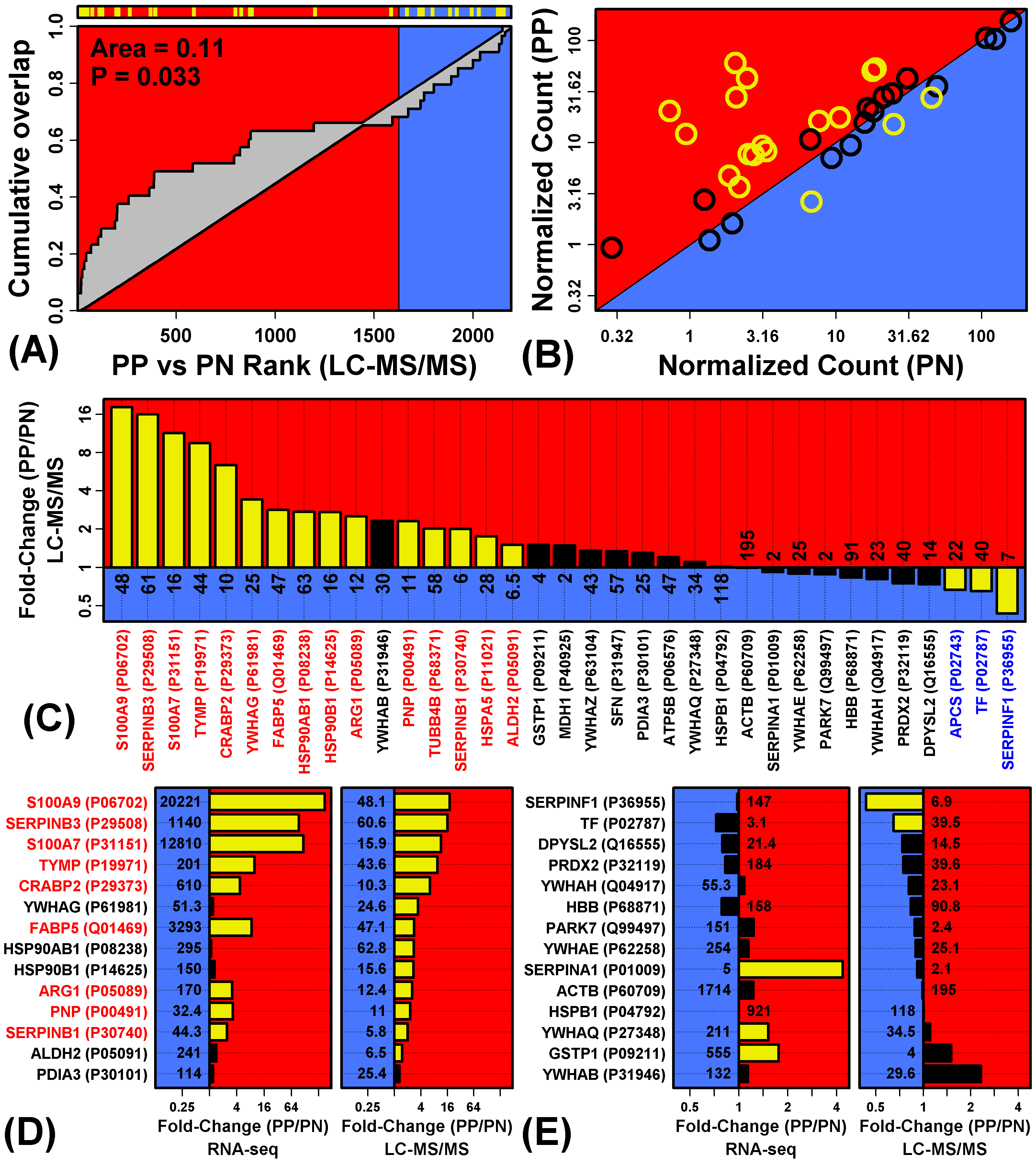

Supplement: Additional file 8: — Differentially expressed proteins previously identified by Ryu et al. [20]. Ryu et al. identified 36 proteins with significantly increased abundance in psoriasis lesions compared with normal skin. a The 36 proteins significantly enriched among the PP-increased proteins identified in our analysis (p = 0.033). Proteins we detected by LC-MS/MS were ranked in descending order according to the estimated PP/PN fold-change (horizontal axis; red, PP-increased; blue, PP-decreased). The cumulative overlap between the 36 proteins and this ranked protein list is shown. Yellow hash marks (top) denote placement of the 36 proteins relative to the ranked protein list from our analysis. b Association between SpC values in PP and PN skin for the 36 proteins (yellow symbols indicate DEPs). c LC-MS/MS-estimated fold changes for each of the 36 proteins (n = 14 patients; yellow bars indicate DEPs). d Twelve of the 36 proteins showing the strongest mRNA and protein increase. e Twelve of the 36 proteins showing the strongest mRNA and protein decrease. (TIFF 1598 kb) [file 13073_2015_208_MOESM8_ESM.tif]

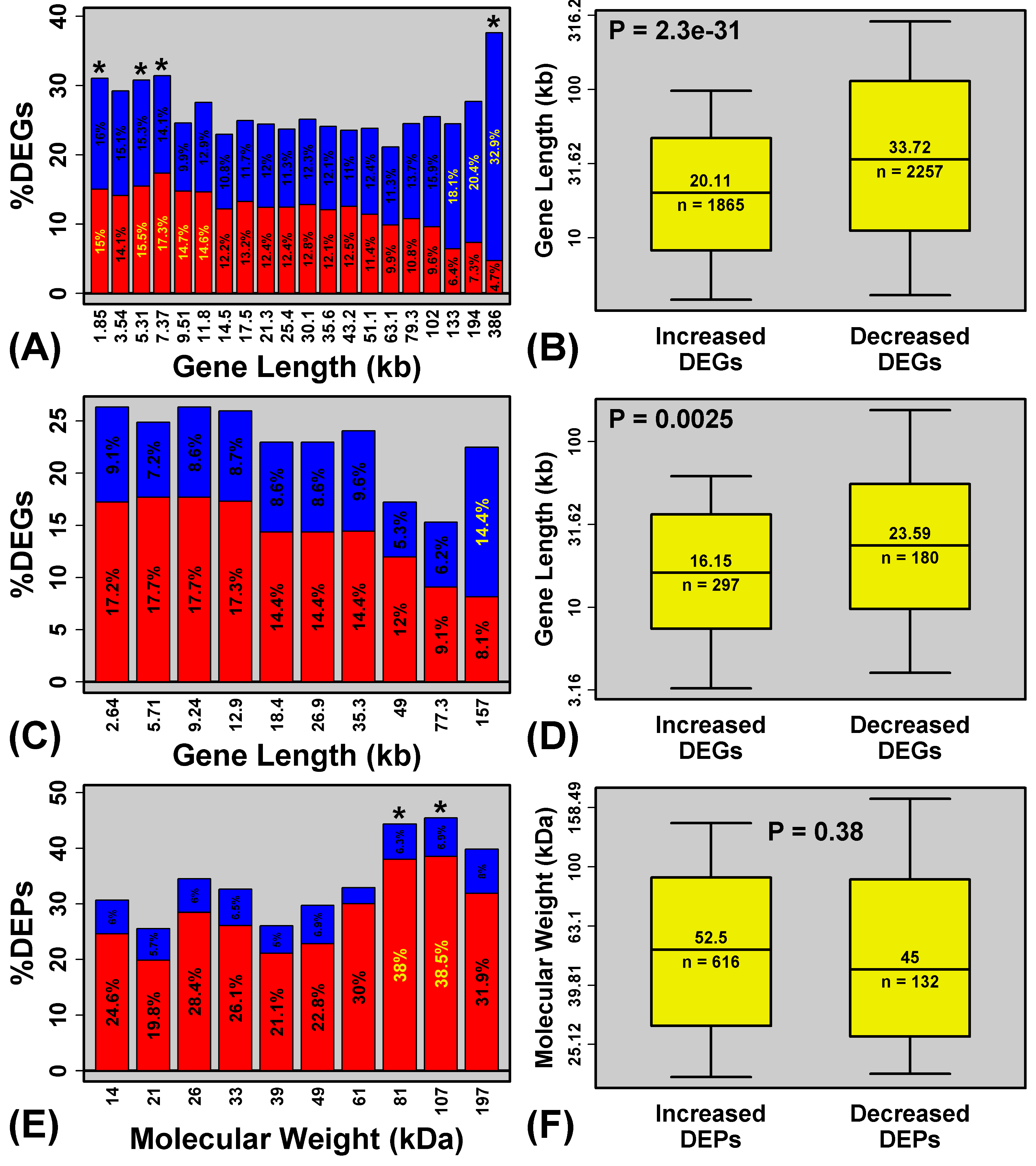

Supplement: Additional file 9: — Shifts in protein abundance in psoriasis are not associated with molecular weight, despite gene length bias affecting mRNA differential expression. a Gene length bias affecting differential mRNA expression in psoriasis (15,616 skin-expressed genes). The figure shows the percentage of DEGs among genes within different bins varying by gene length (red, percentage of PP-increased DEGs; blue, percentage of PP-decreased DEGs). The percentage of PP-increased and PP-decreased DEGs is listed within each bar (yellow: p < 0.05, Fisher’s exact test). Asterisks above bars indicate significant over-abundance of DEGs (PP-increased plus PP-decreased) with respect to a given gene bin (FDR < 0.05, Fisher’s exact test). b Median gene length among PP-decreased DEGs is greater than among PP-increased DEGs. Boxes outline the middle 50 % of gene lengths in each DEG group (25th percentile, median and 75th percentile; whiskers span the 10th to 90th percentile). The median gene length is listed for each group along with the number of DEGs, with p value generated from the comparison of gene lengths between groups (Wilcoxon rank sum test). Parts (c) and (d) are the same as (a) and (b), respectively, except only 2088 genes associated with detected proteins are analyzed. Similarly, parts (e) and (f) are the same as (a) and (b), respectively, except 2194 proteins are analyzed to assess whether the percentage of PP-increased and PP-decreased DEPs differs according to protein molecular weight (kDa). (TIFF 1532 kb) [file 13073_2015_208_MOESM9_ESM.tif]

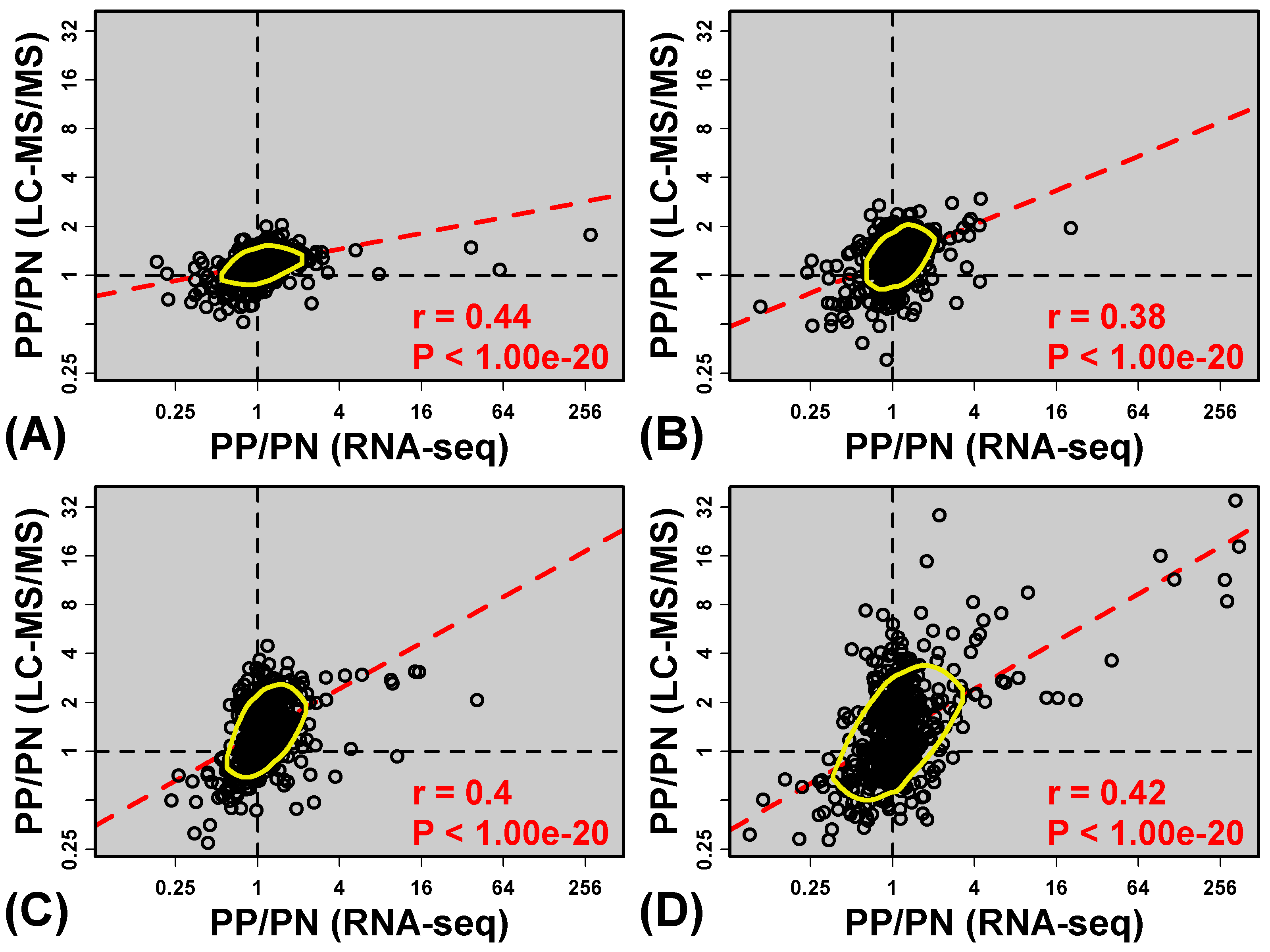

Supplement: Additional file 11: — The association between mRNA (RNA-seq) and protein (LC-MS/MS) fold changes (PP/PN) does not differ between low- and high-expressed genes. The 2087 mRNA–protein pairs were divided into four groups based upon average FPKM. Average FPKM was calculated with respect to PP and PN samples, respectively, and the higher of the two values was used to assign mRNA–protein pairs to each group (approximately 520 pairs per group). The figure shows the association between RNA-seq- and LC-MS/MS-estimated fold changes for mRNAs with average FPKM values beneath the 25th percentile (a), within the 25–50th percentile (b), within the 50–75th percentile (c), and above the 75th percentile (d). Dashed red lines represent least-square regression estimates and yellow ellipses encompass the 50 % of proteins nearest to the bivariate mean (Mahalanobis distance). (TIFF 185 kb) [file 13073_2015_208_MOESM11_ESM.tif]

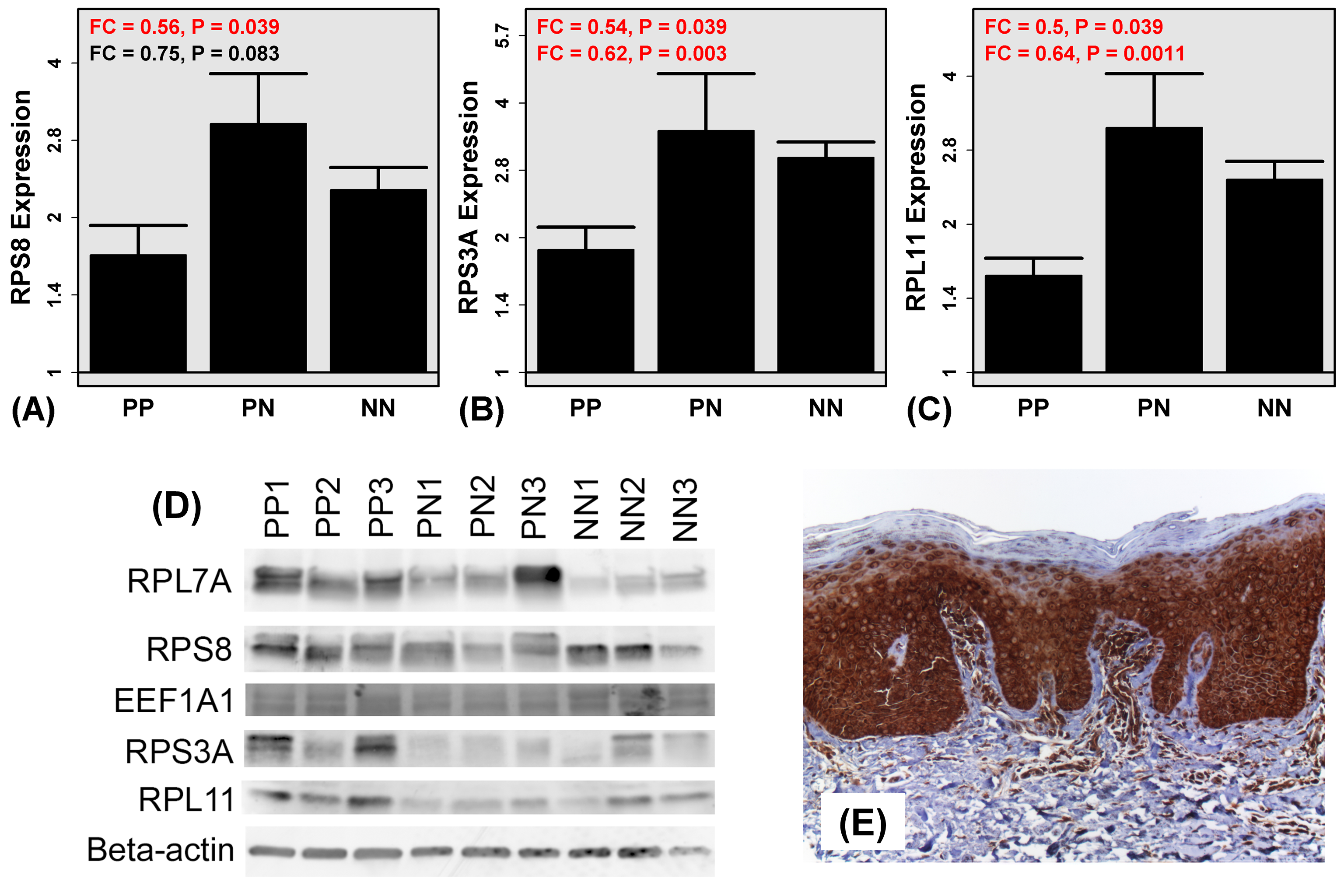

Supplement: Additional file 12: — Decreased abundance of ribosomal subunit mRNA and protein in psoriasis lesions. a–c RT-PCR was used to measure expression of three genes in lesional skin from psoriasis patients (PP), uninvolved skin from psoriasis patients (PN), and normal skin from healthy control subjects (NN) (n = 8 per group; ±1 standard error). Gene expression for each sample is normalized to the expression of glyceraldehyde-3-phosphate dehydrogenase (GAPDH). Fold-changes from p values are listed for the comparison between PP and PN groups (top; paired Wilcoxon rank sum test) and between PP and NN groups (bottom; Wilcoxon rank sum test). b Western blot analysis of RPL7A, RPS8, EEF1A1, RPS3A and RPL11 in PP, PN and NN skin (n = 3 per group). c Immunohistochemical staining of RPL7A in lesional psoriasis skin. (TIFF 3429 kb) [file 13073_2015_208_MOESM12_ESM.tif]

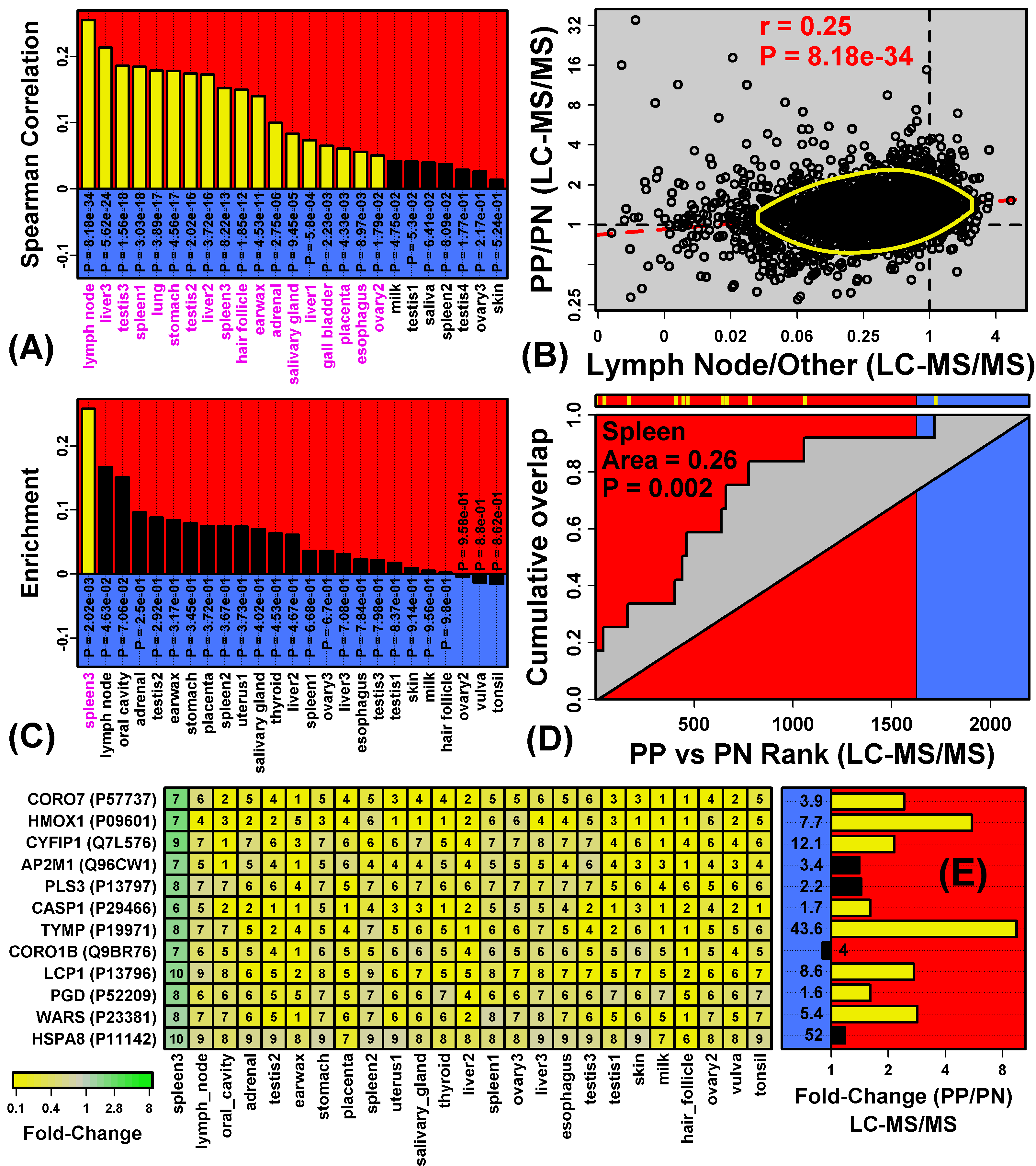

Supplement: Additional file 13: — Proteins expressed in lymphoid organs are elevated in psoriasis lesions (ProteomicsDB). a ProteomicsDB cell types ranked by the correlation between cell type-specific expression and LC-MS/MS-estimated fold change (PP/PN; 2198 proteins). b Association between cell type-specific expression and fold change (PP/PN) for lymph node. c ProteomicsDB cell types ranked according to how strongly the 12 best signature proteins for each cell type are enriched among those proteins with elevated abundance in PP skin. d Enrichment of spleen signature proteins among PP-increased proteins. e Spleen signature proteins and their relative abundance across ProteomicsDB cell types. See Fig. 4 legend for further details. (TIFF 1865 kb) [file 13073_2015_208_MOESM13_ESM.tif]

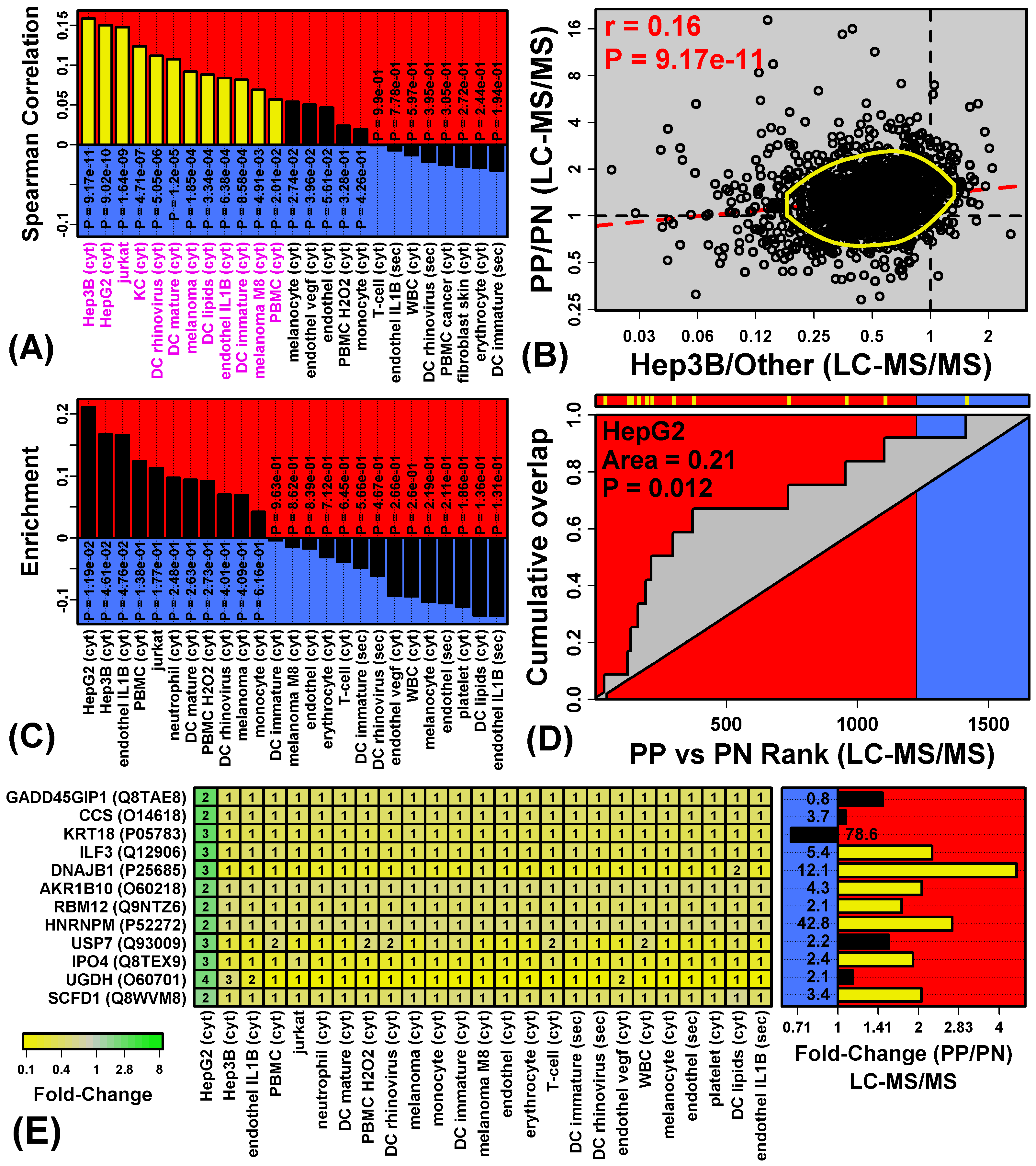

Supplement: Additional file 14: — Proteins expressed in transformed cells are elevated in psoriasis lesions (CPL/MUW database). a CPL/MUW database cell types ranked by the correlation between cell type-specific expression and LC-MS/MS-estimated fold change (PP/PN; 1656 proteins). b Association between cell type-specific expression and fold change (PP/PN) for Hep3B cells. c CPL/MUW database cell types ranked according to how strongly the 12 best signature proteins for each cell type are enriched among those proteins with elevated abundance in PP skin. d Enrichment of HepG2 signature proteins among PP-increased proteins. e HepG2 signature proteins and their relative abundance across CPL/MUW cell types. See Fig. 4 legend for further details. (TIFF 1652 kb) [file 13073_2015_208_MOESM14_ESM.tif]

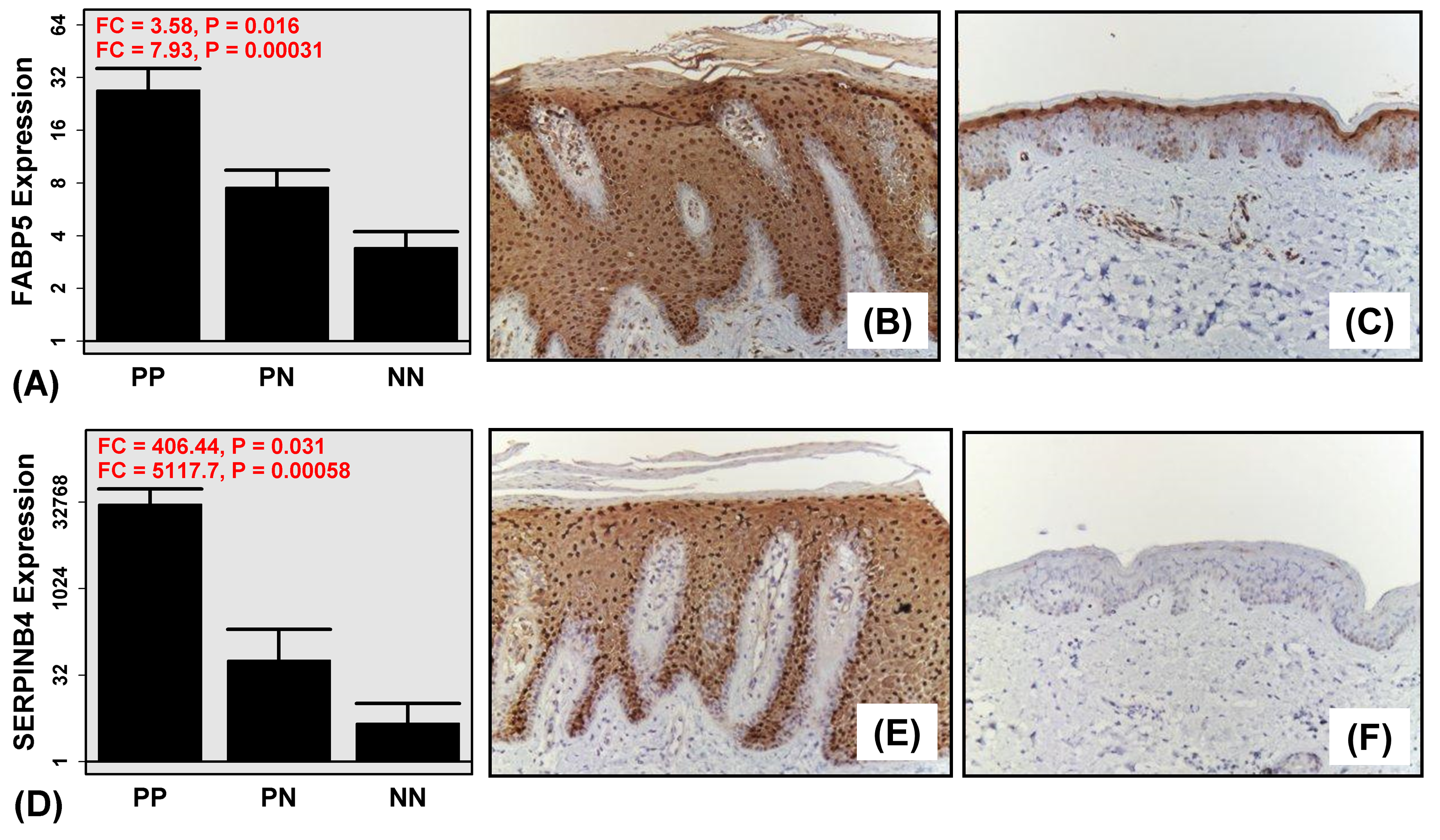

Supplement: Additional file 15: — Fatty acid binding protein 5 (FABP5) and serpin peptidase inhibitor 4 (SERPINB4) show correspondent increases in mRNA and protein abundance in psoriasis lesions. (A) Expression of FABP5 in lesional (PP), uninvolved (PN) and normal skin from healthy controls (NN) (RT-PCR; n = 8 per group). (B) FABP5 immunohistochemistry staining in PP skin. (C) FABP5 immunohistochemistry staining in PN skin. (D) Expression of SERPINB4 in lesional (PP), uninvolved (PN) and normal skin from healthy controls (NN) (RT-PCR; n = 8 per group). For RT-PCR analyses (A and D), two sets of fold-changes and p values are listed (top: PP versus PN; bottom: PP versus NN). Gene expression for each sample is normalized to glyceraldehyde-3-phosphate dehydrogenase (GAPDH). (TIFF 4624 kb) [file 13073_2015_208_MOESM15_ESM.tif]

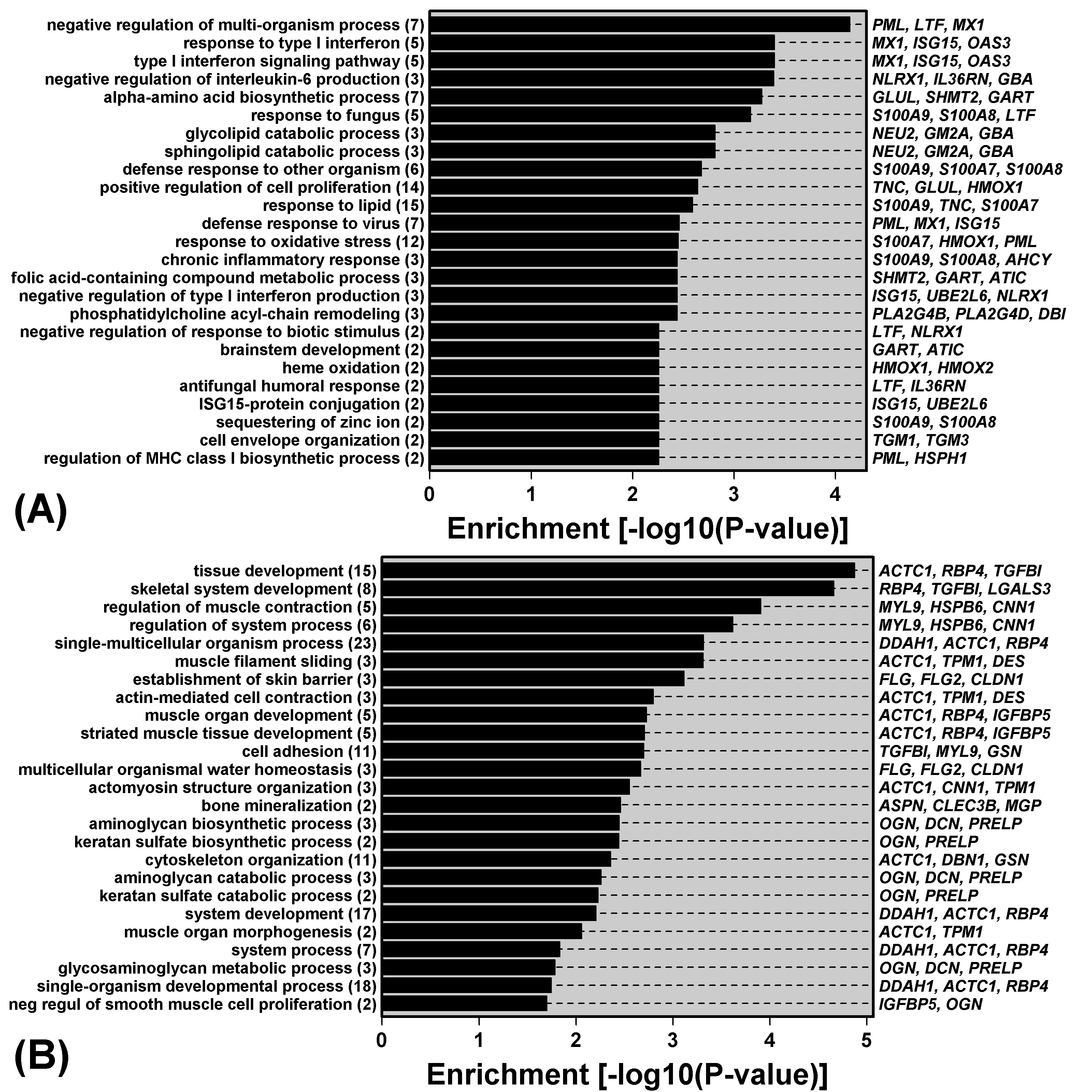

Supplement: Additional file 16: — GO biological process terms enriched among PP-increased DEGPs and PP-decreased DEGPs. a Top-ranked GO biological process (BP) terms enriched among the 153 PP-increased DEGPs. b Top-ranked GO BP terms enriched among the 56 PP-decreased DEGPs. In both (a) and (b), enrichment was evaluated with respect to a background set of 2087 genes associated with mRNAs and proteins detected using RNA-seq and LC-MS/MS, respectively. The right margin lists example DEGPs associated with each GO BP term. (TIFF 1028 kb) [file 13073_2015_208_MOESM16_ESM.tif]

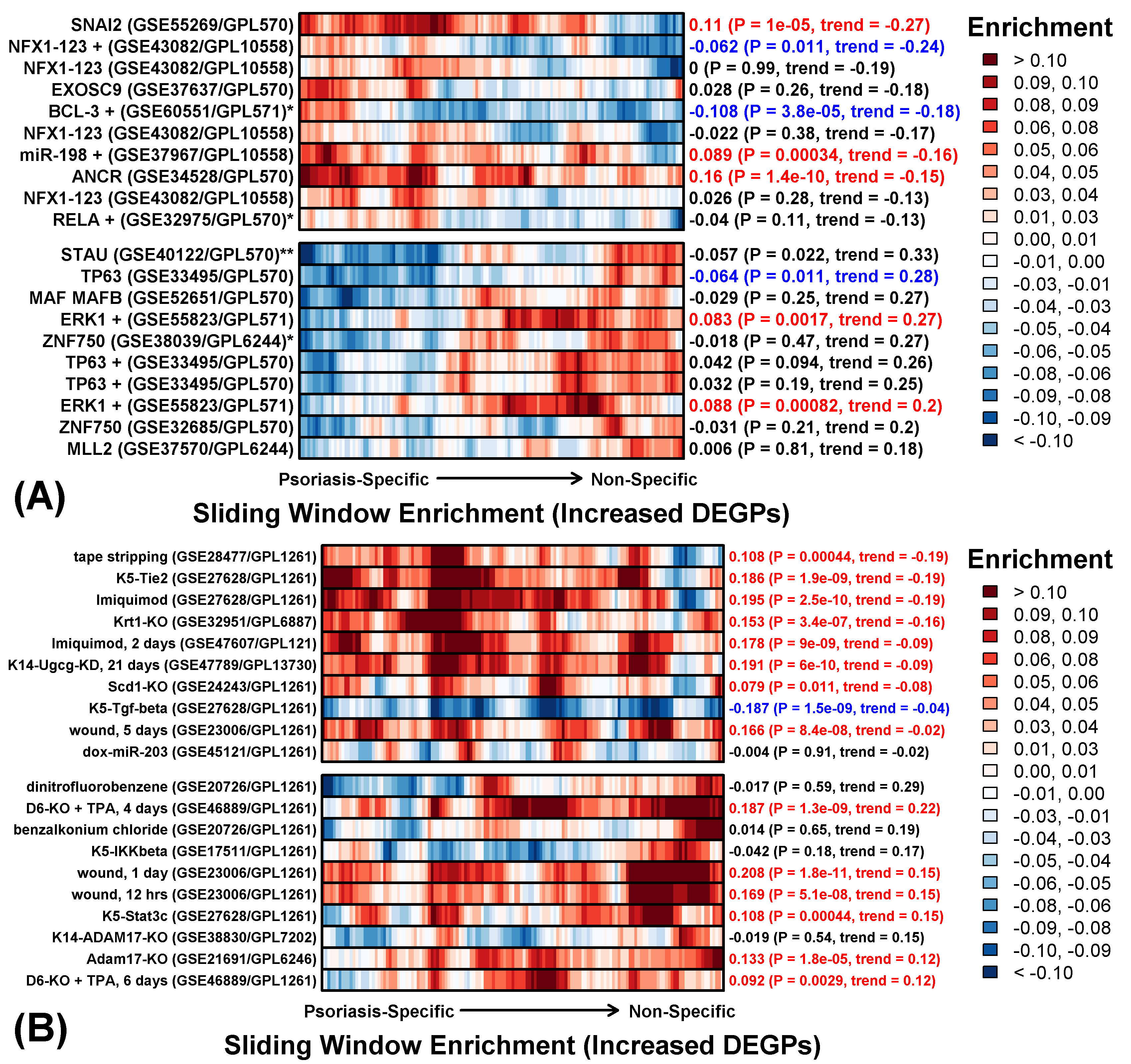

Supplement: Additional file 18: — Psoriasis-specific and non-specific DEGPs show divergent responses in KCs following gene perturbations and in mouse skin phenotypes. Analyses shown in Figs. 6 and 7 were repeated with respect to KC gene perturbations (a) (65 lists; *HaCaT; **reconstituted epidermis; “ + ” denotes overexpression of a gene rather than RNA interference knockdown) and laboratory mouse phenotypes (b) (35 lists). For each ordered gene list set, the sliding window approach was used to identify the ten lists with the strongest enrichment differences between psoriasis-specific DEGPs and non-specific DEGPs (see Figs. 6 and 7 legends). (TIFF 2368 kb) [file 13073_2015_208_MOESM18_ESM.tif]
