# Supplementary material for: Proteogenomic analysis of psoriasis reveals discordant and concordant changes in mRNA and protein abundance
Source: Genome Med. 2015 Aug 4;7(1):86. doi: 10.1186/s13073-015-0208-5 (PMC4527112; doi:10.1186/s13073-015-0208-5)
Supplement: Additional file 6: — Association between mRNA (FPKM) and protein (NSAF) abundance for each patient sample. Scatterplots show the correlation between FPKM and NSAF values with respect to each individual patient sample (PP and PN). Dashed red lines represent the least-squares regression estimate and yellow ellipses encompass the 50 % of proteins nearest to the bivariate median (Mahalanobis distance). (PDF 1055 kb) [file 13073_2015_208_MOESM6_ESM.pdf]

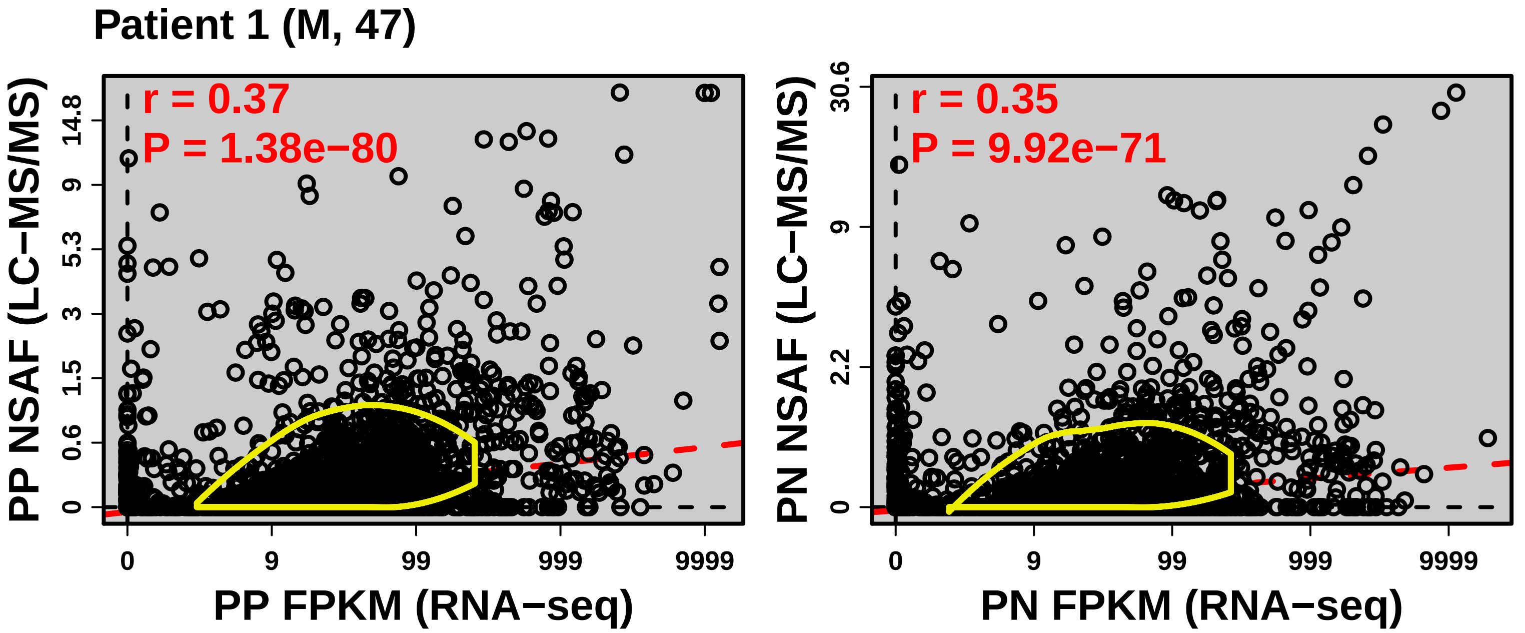
Patient 1 (M, 47)
r = 0.37
P = 1.38e−80
PP NSAF (LC−MS/MS)
0
0.6
1.5
3
5.3
9
14.8
0
9
99
999
9999
PP FPKM (RNA−seq)
r = 0.35
P = 9.92e−71
PN NSAF (LC−MS/MS)
0
2.2
9
30.6
0
9
99
999
9999
PN FPKM (RNA−seq)

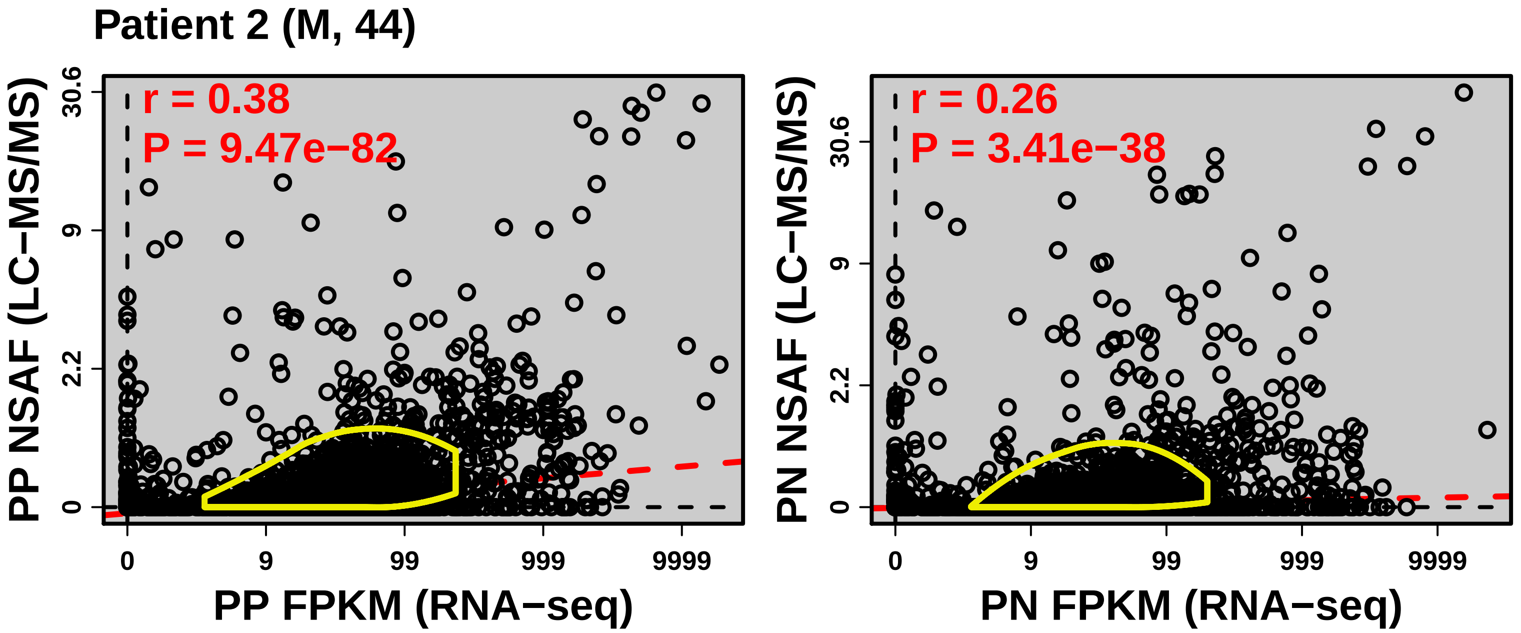

Patient 2 (M, 44)
r = 0.38
P = 9.47e−82
PP NSAF (LC−MS/MS)
PP FPKM (RNA−seq)
30.6
9
2.2
0
0
9
99
999
9999
r = 0.26
P = 3.41e−38
PN NSAF (LC−MS/MS)
PN FPKM (RNA−seq)

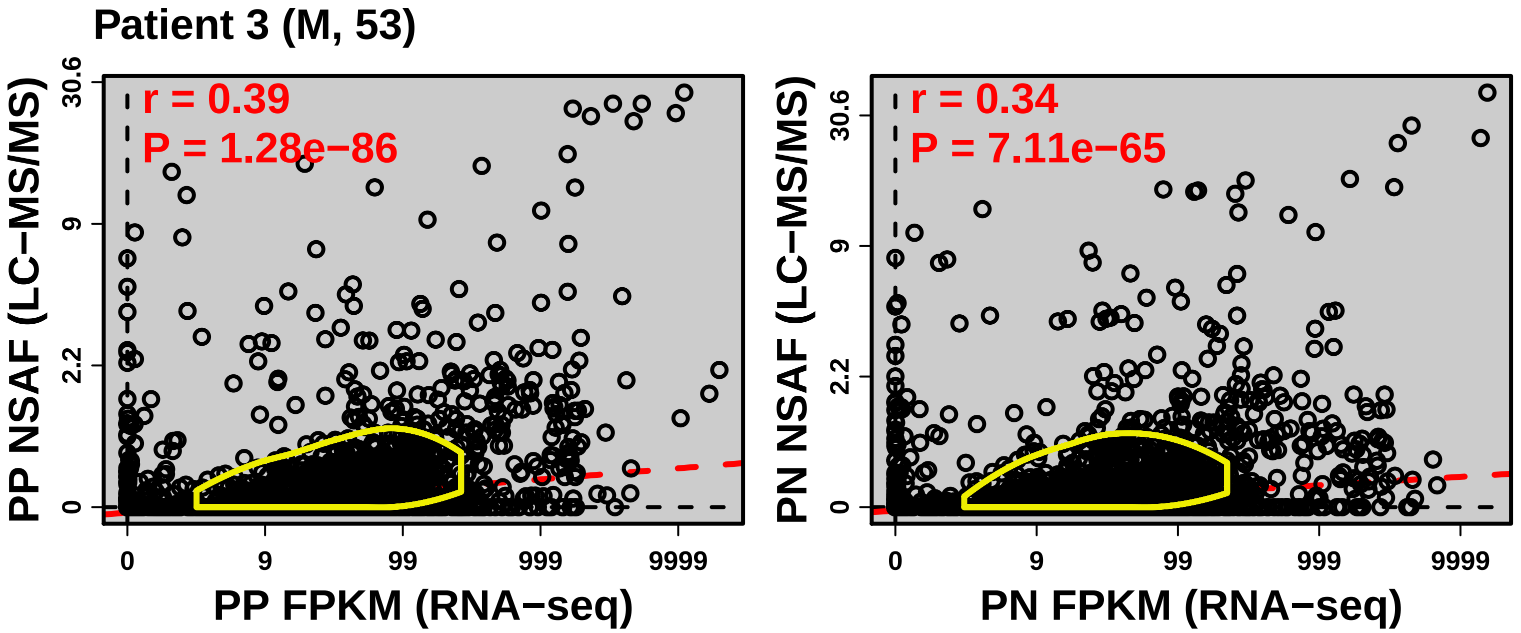

Patient 3 (M, 53)
r = 0.39
P = 1.28e−86
PP NSAF (LC−MS/MS)
30.6
9
2.2
0
0
9
99
999
9999
PP FPKM (RNA−seq)
r = 0.34
P = 7.11e−65
PN NSAF (LC−MS/MS)
30.6
9
2.2
0
0
9
99
999
9999
PN FPKM (RNA−seq)

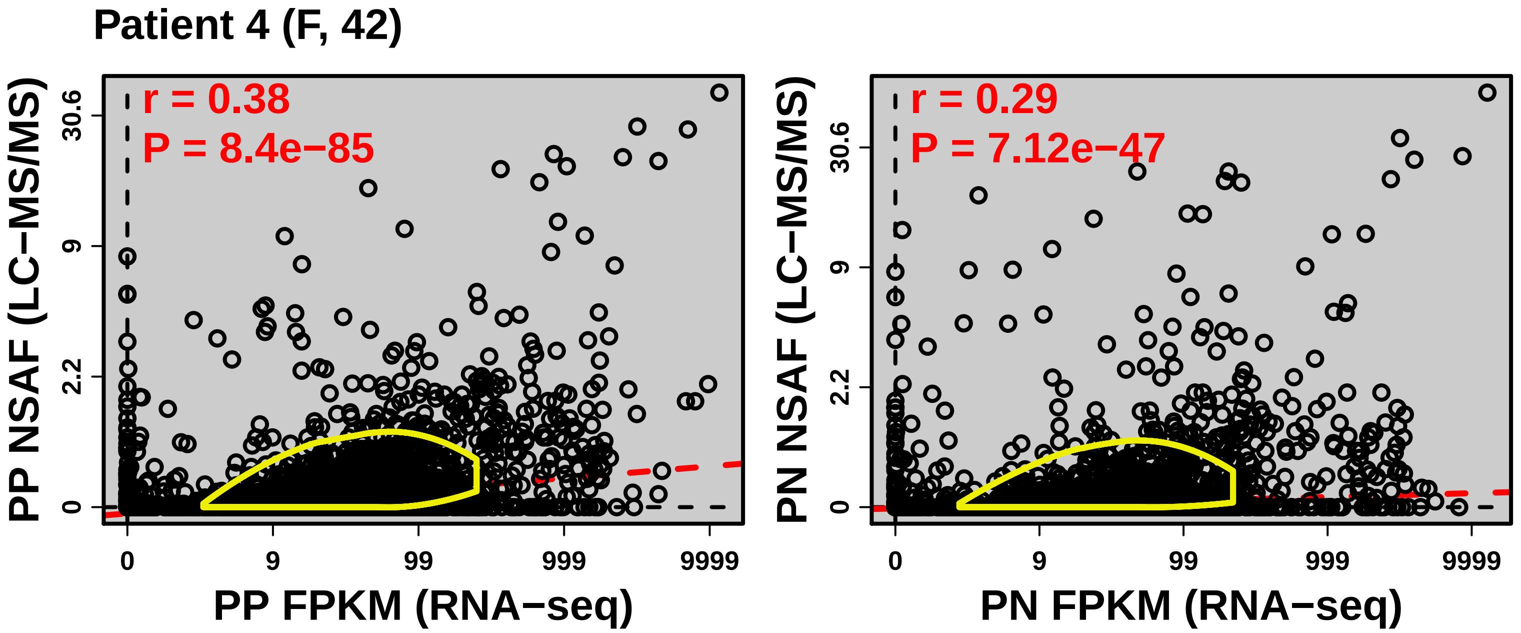

Patient 4 (F, 42)
r = 0.38
P = 8.4e−85
PP NSAF (LC−MS/MS)
PP FPKM (RNA−seq)
0
2.2
9
30.6
0
9
99
999
9999
r = 0.29
P = 7.12e−47
PN NSAF (LC−MS/MS)
PN FPKM (RNA−seq)

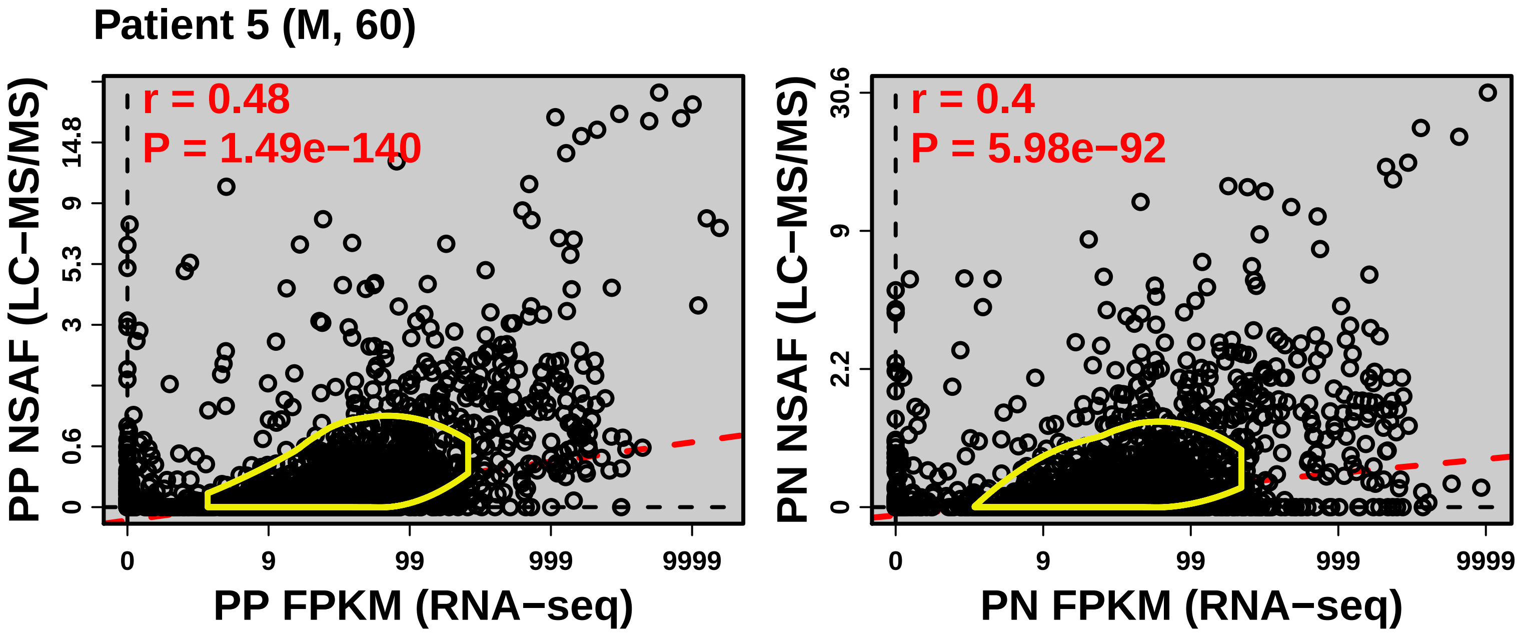

Patient 5 (M, 60)
r = 0.48
P = 1.49e−140
PP NSAF (LC−MS/MS)
0
0.6
3
5.3
9
14.8
9
99
999
9999
PP FPKM (RNA−seq)
r = 0.4
P = 5.98e−92
PN NSAF (LC−MS/MS)
2.2
30.6
PN FPKM (RNA−seq)

Patient 6 (F, 56)

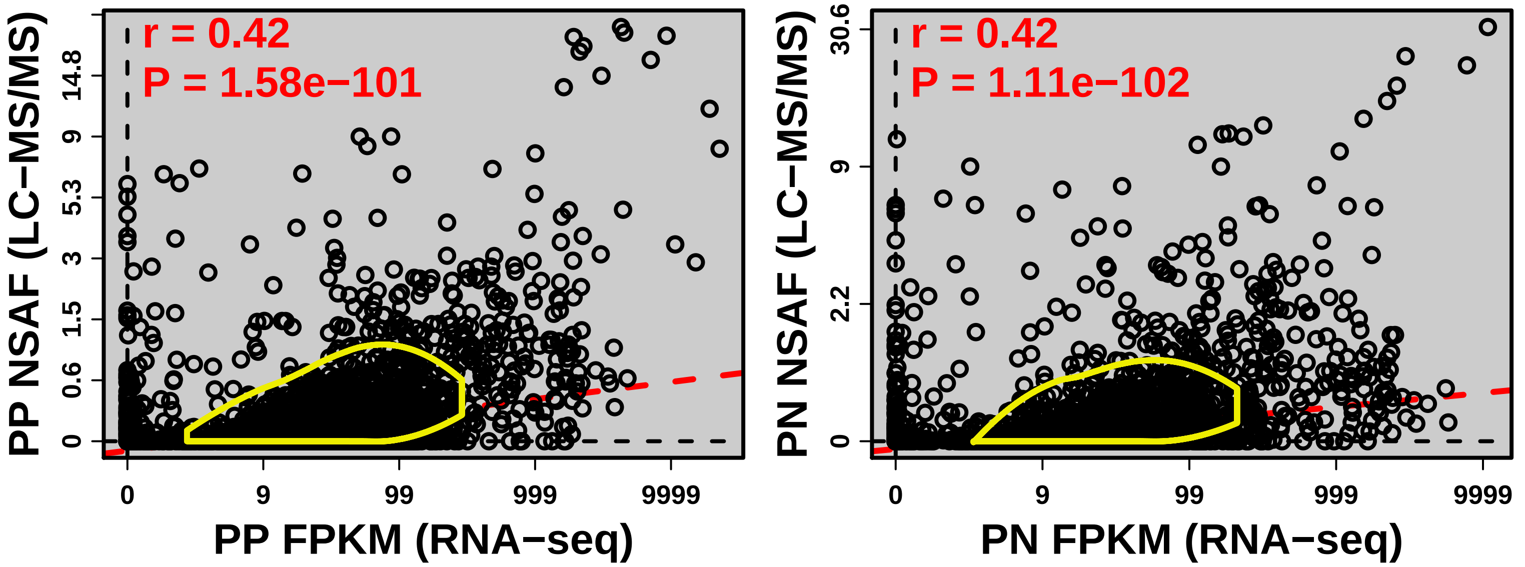

Patient 7 (M, 62)

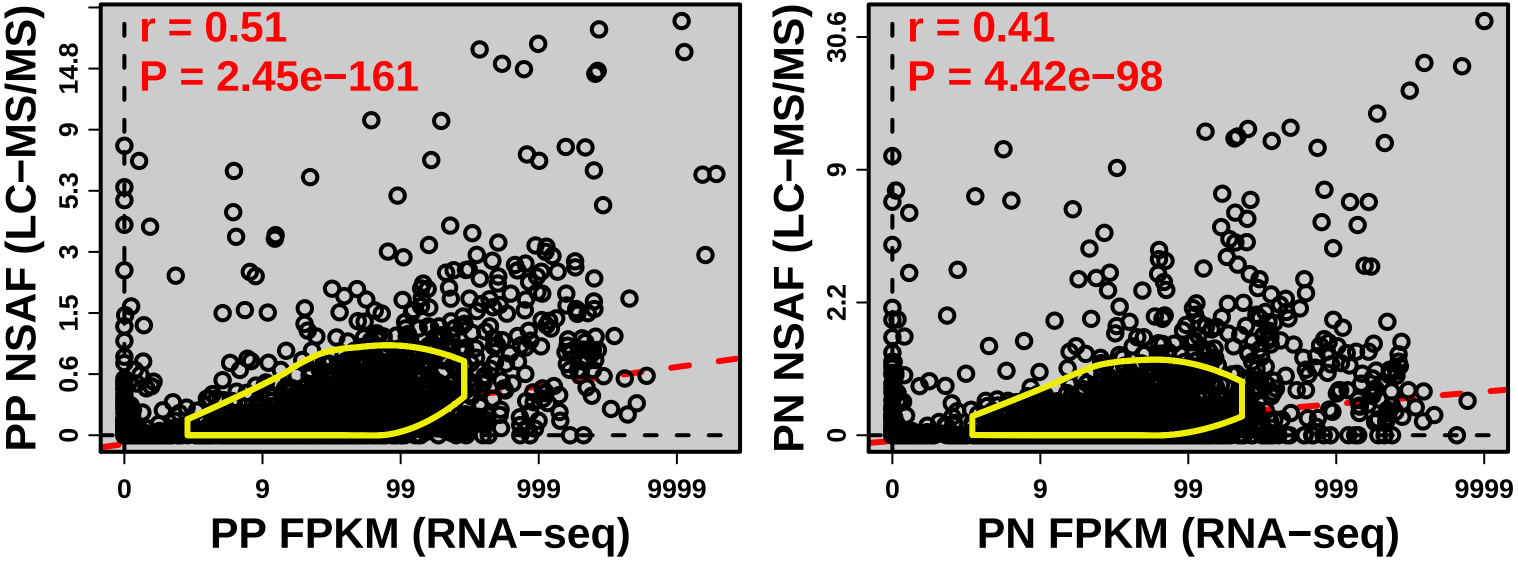

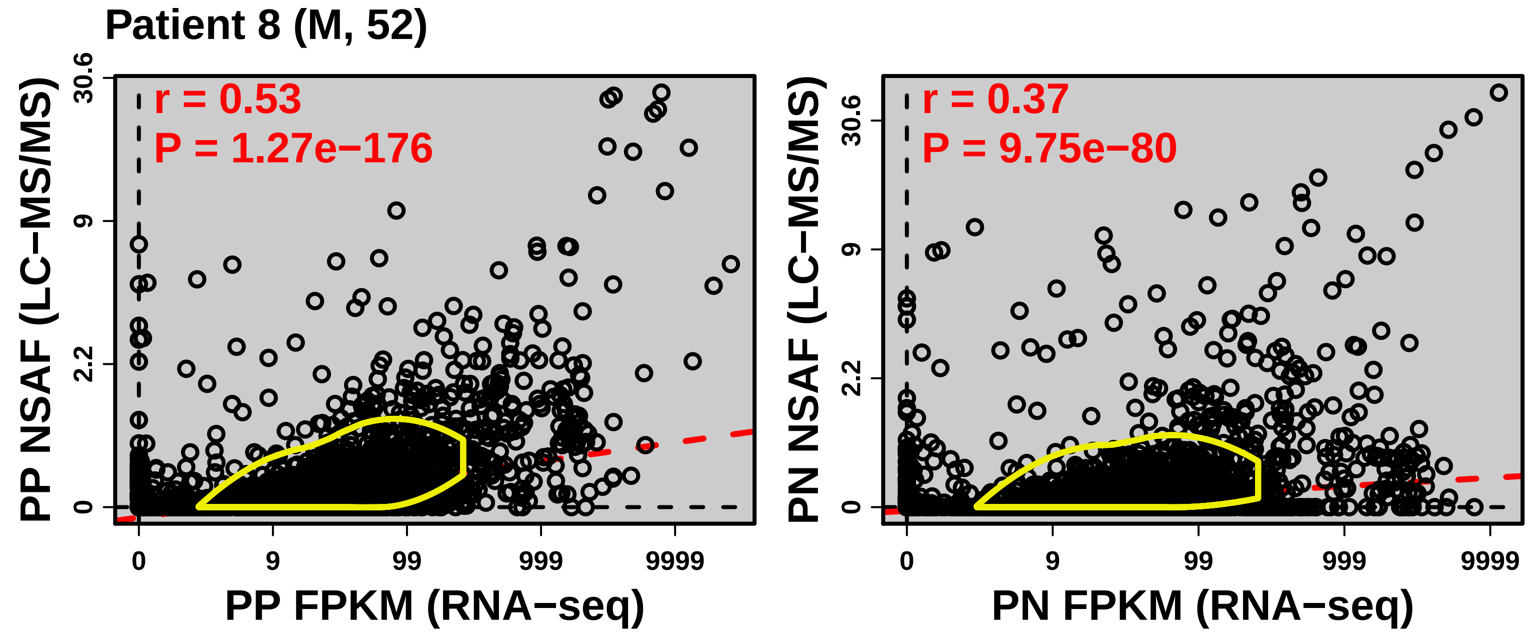
Patient 8 (M, 52)
r = 0.53
P = 1.27e−176
PP NSAF (LC−MS/MS)
PP FPKM (RNA−seq)
r = 0.37
P = 9.75e−80
PN NSAF (LC−MS/MS)
PN FPKM (RNA−seq)
0
9
99
999
9999
2.2
30.6

Patient 9 (M, 71)

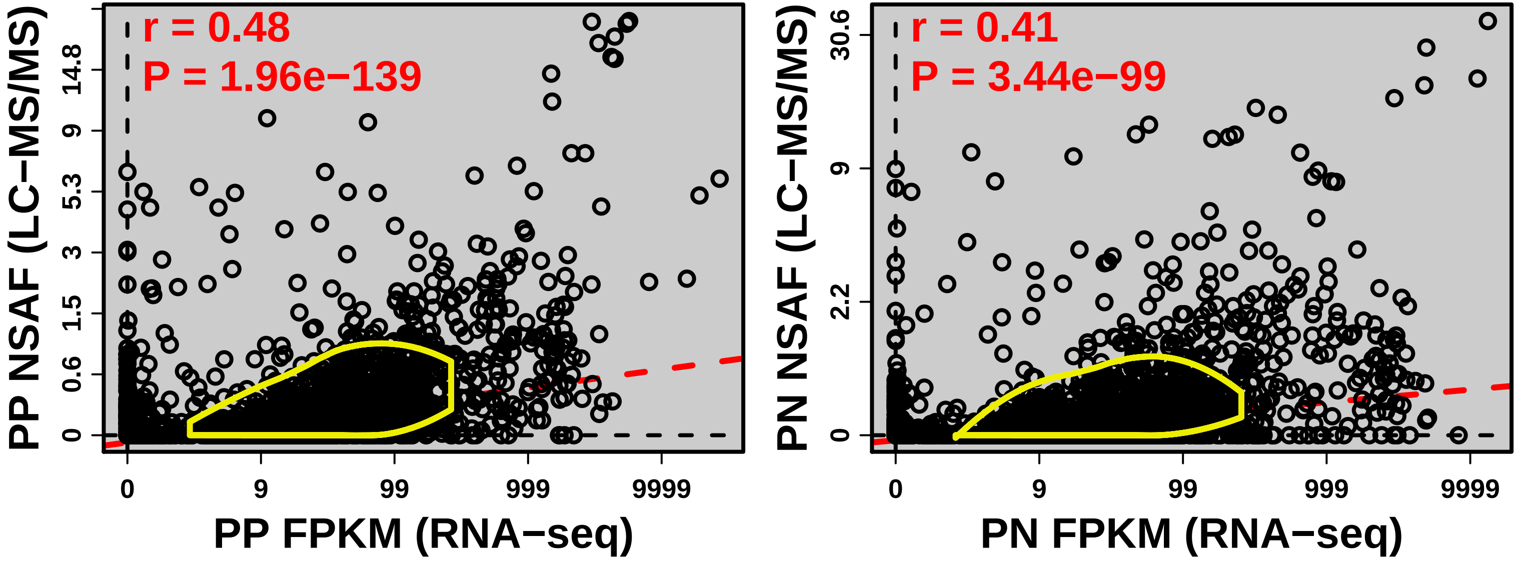

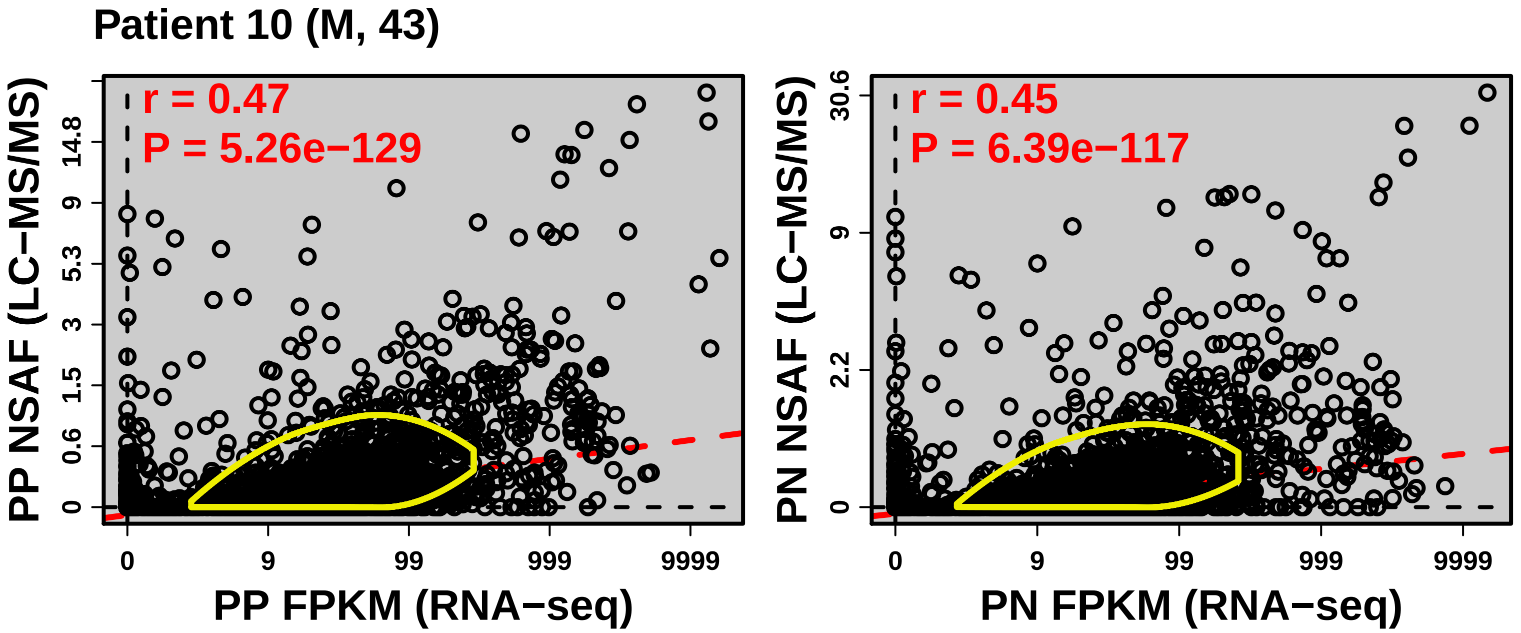

Patient 10 (M, 43)
r = 0.47
P = 5.26e−129
PP NSAF (LC−MS/MS)
PP FPKM (RNA−seq)
0
0.6
1.5
3
5.3
9
14.8
0
9
99
999
9999
r = 0.45
P = 6.39e−117
PN NSAF (LC−MS/MS)
PN FPKM (RNA−seq)
0
2.2
9
30.6
0
9
99
999
9999

Patient 11 (F, 57)

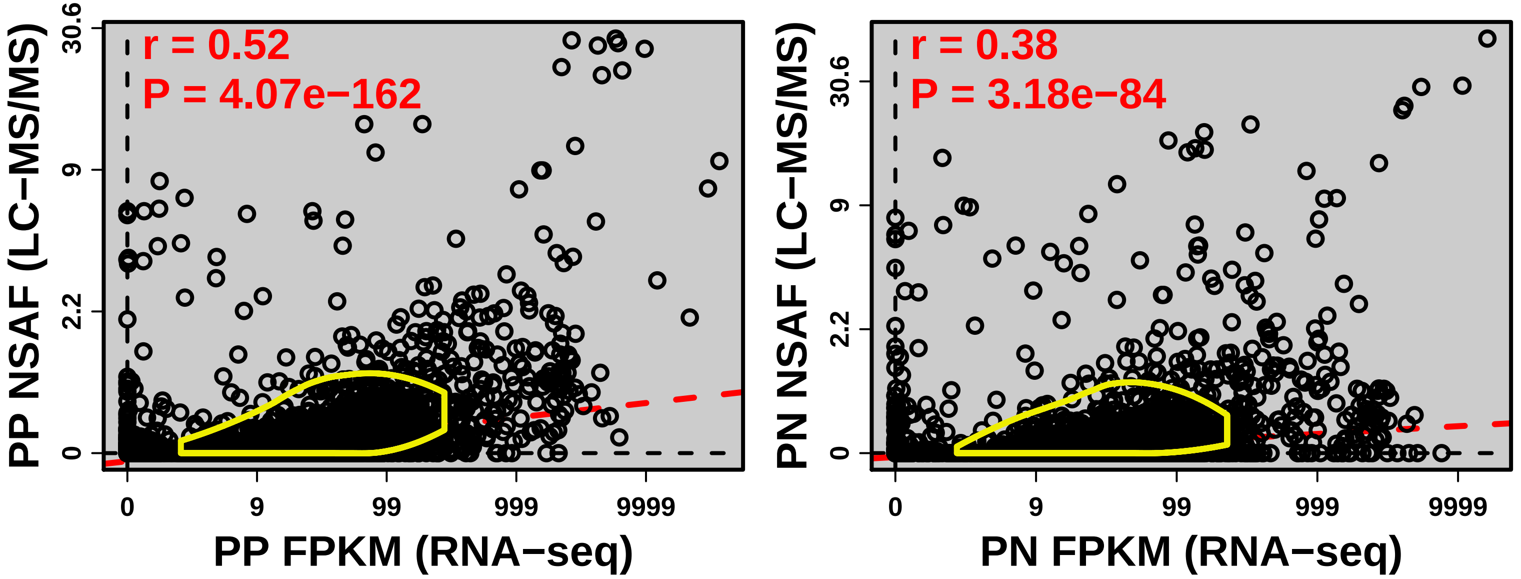

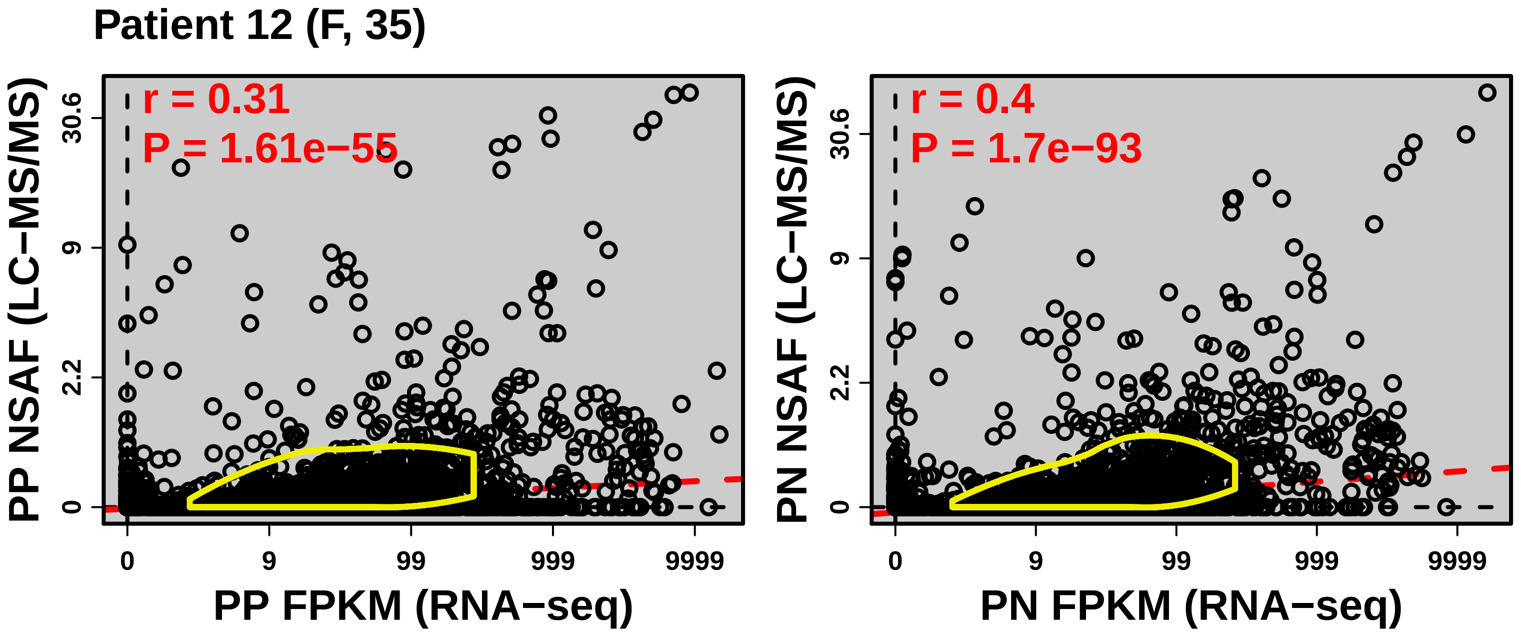
Patient 12 (F, 35)
r = 0.31
P = 1.61e−55
PP NSAF (LC−MS/MS)
PP FPKM (RNA−seq)
0
2.2
9
30.6
0
9
99
999
9999
r = 0.4
P = 1.7e−93
PN NSAF (LC−MS/MS)
PN FPKM (RNA−seq)
0
2.2
9
30.6
0
9
99
999
9999

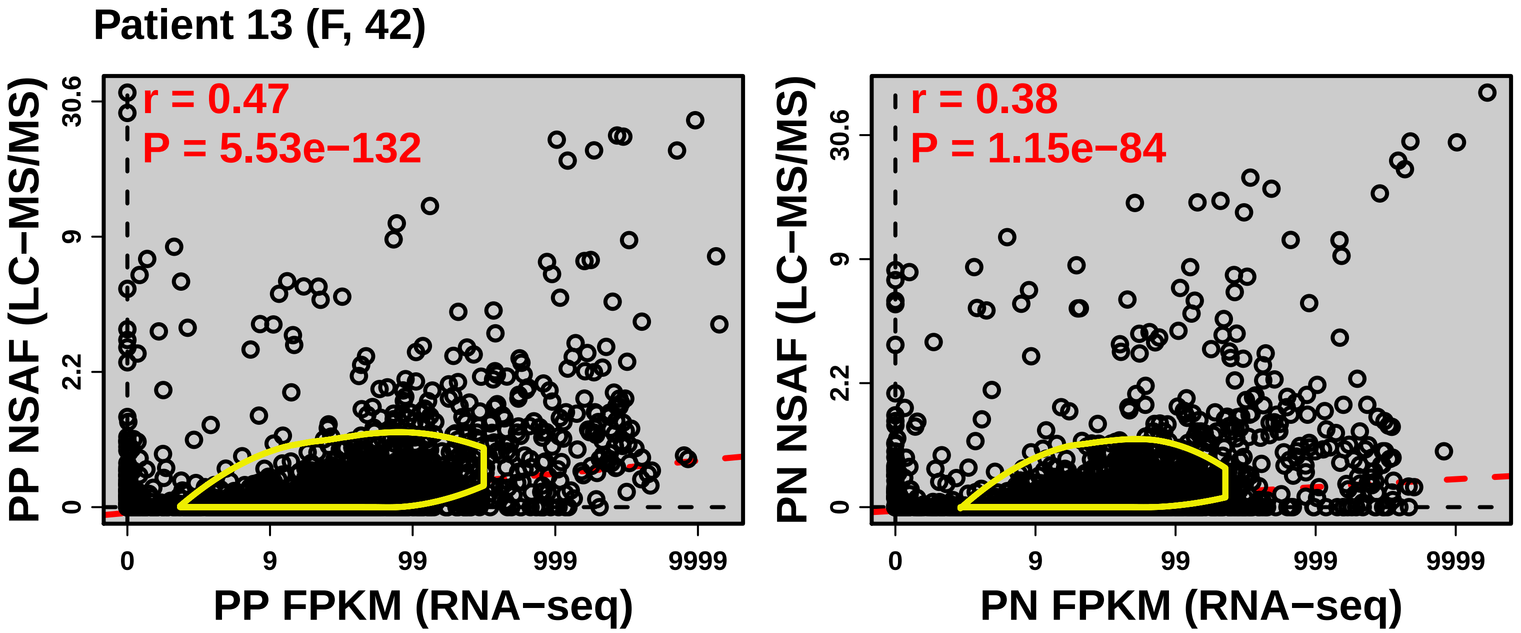
Patient 13 (F, 42)
r = 0.47
P = 5.53e−132
PP NSAF (LC−MS/MS)
PP FPKM (RNA−seq)
0
2.2
9
30.6
0
9
99
999
9999
r = 0.38
P = 1.15e−84
PN NSAF (LC−MS/MS)
PN FPKM (RNA−seq)

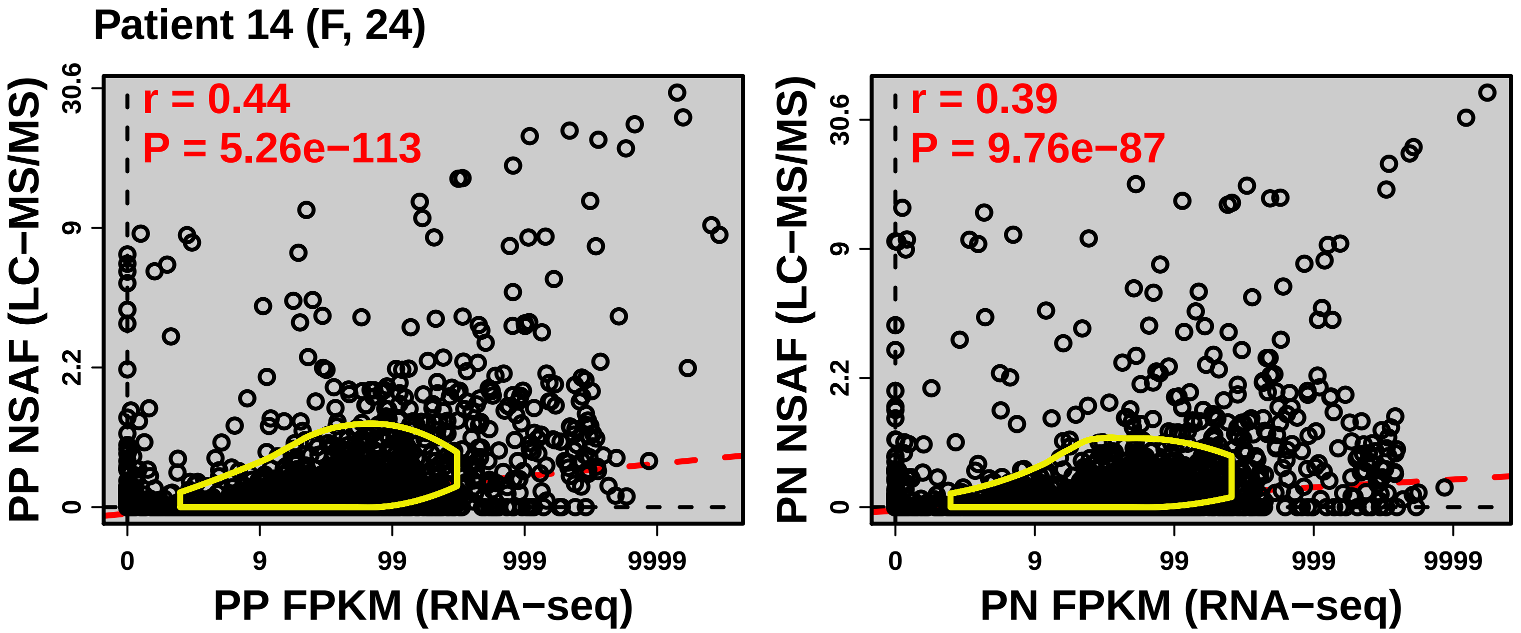
Patient 14 (F, 24)
r = 0.44
P = 5.26e−113
PP NSAF (LC−MS/MS)
PP FPKM (RNA−seq)
0
9
99
999
9999
2.2
30.6
r = 0.39
P = 9.76e−87
PN NSAF (LC−MS/MS)
PN FPKM (RNA−seq)
