# Supplementary material for: Proteogenomic analysis of psoriasis reveals discordant and concordant changes in mRNA and protein abundance
Source: Genome Med. 2015 Aug 4;7(1):86. doi: 10.1186/s13073-015-0208-5 (PMC4527112; doi:10.1186/s13073-015-0208-5)
Supplement: Additional file 10: — Association between mRNA (RNA-seq) and protein (LC-MS/MS) fold changes (PP/PN) with respect to individual patients. Fold changes generated by RNA-seq and LC-MS/MS were compared for each individual patient (2087 mRNA–protein pairs). Dashed red lines represent least-square regression estimates and yellow ellipses encompass 50 % of proteins nearest to the bivariate mean (Mahalanobis distance). (PDF 459 kb) [file 13073_2015_208_MOESM10_ESM.pdf]

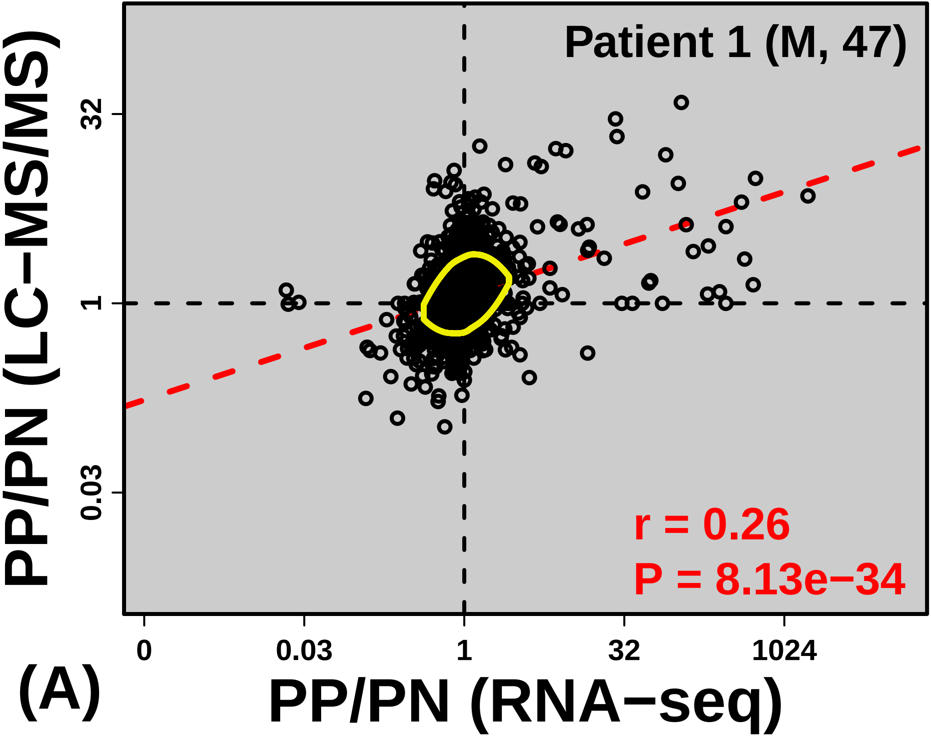

Patient 1 (M, 47)
PP/PN (LC−MS/MS)
32
1
0.03
r = 0.26
P = 8.13e−34
0
0.03
1
32
1024
PP/PN (RNA−seq)

(A)

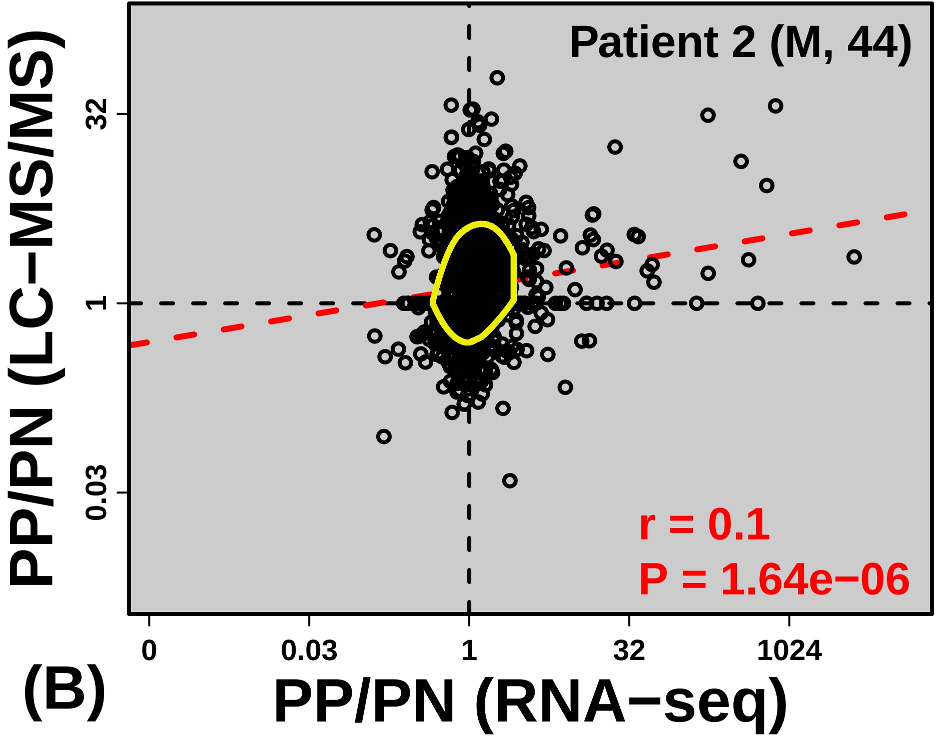

Patient 2 (M, 44)
PP/PN (LC−MS/MS)
32
1
0.03
0
0.03
1
32
1024
r = 0.1
P = 1.64e−06
PP/PN (RNA−seq)

(B)

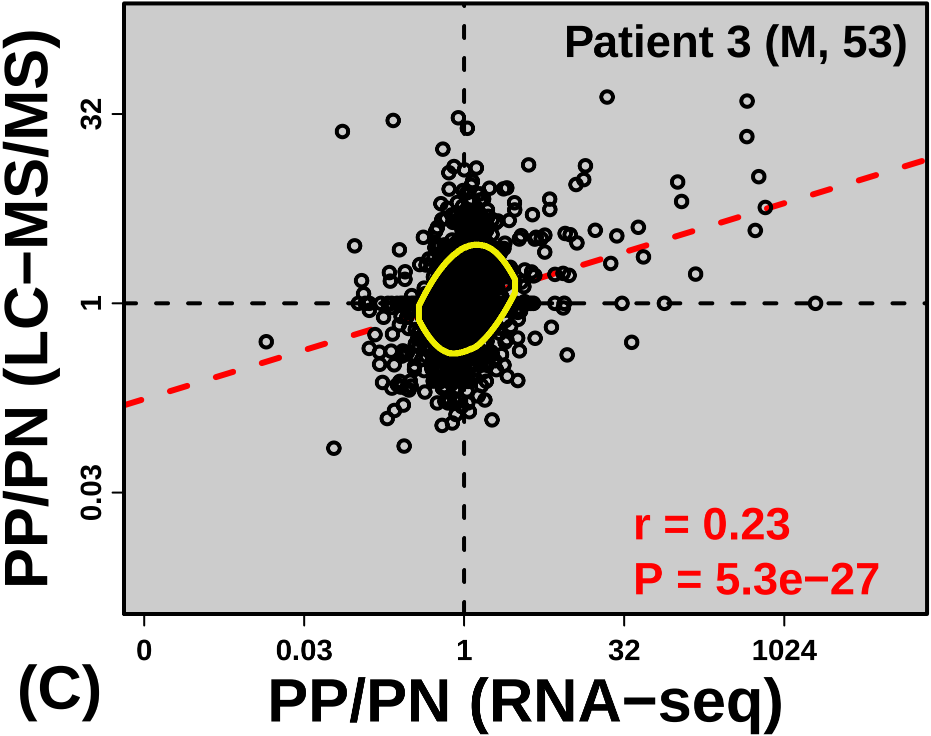

Patient 3 (M, 53)
r = 0.23
P = 5.3e−27
32
1
0.03
0
0.03
1
32
1024
PP/PN (LC−MS/MS)
PP/PN (RNA−seq)

(C)

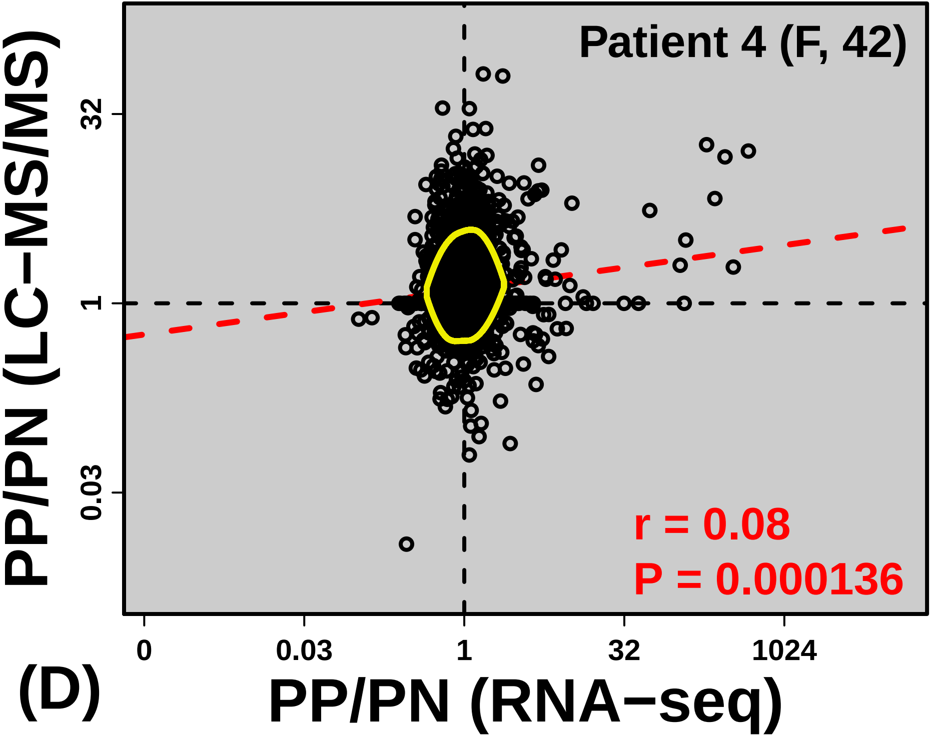
Patient 4 (F, 42)
r = 0.08
P = 0.000136
PP/PN (LC−MS/MS)
32
1
0.03
0
0.03
1
32
1024
PP/PN (RNA−seq)
(D)

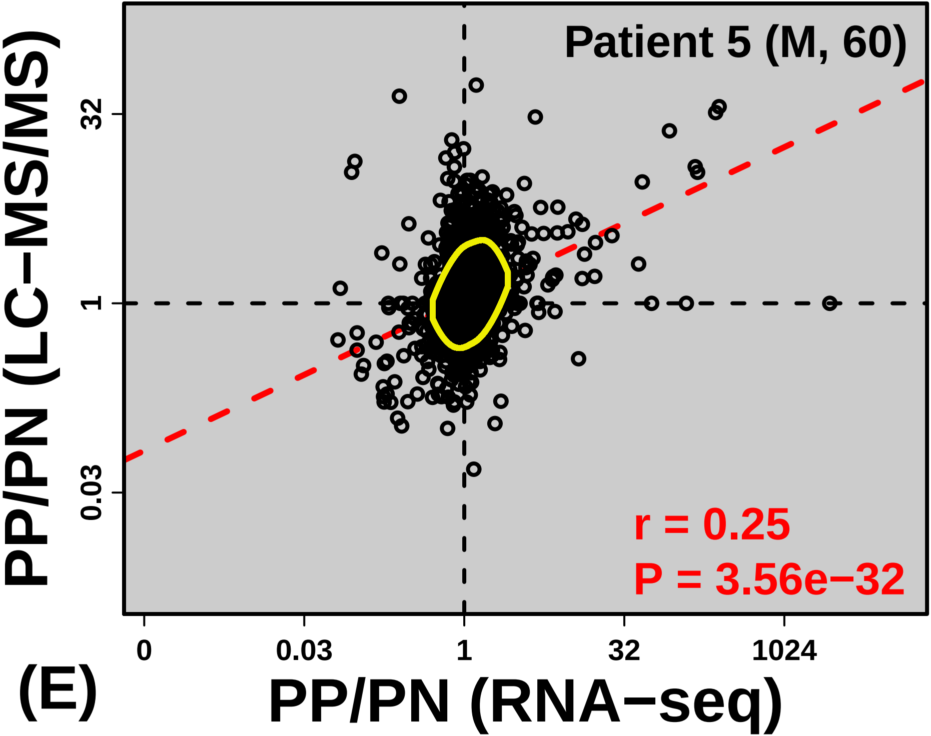

Patient 5 (M, 60)
r = 0.25
P = 3.56e−32
PP/PN (LC−MS/MS)
32
1
0.03
0
0.03
1
32
1024
PP/PN (RNA−seq)

(E)

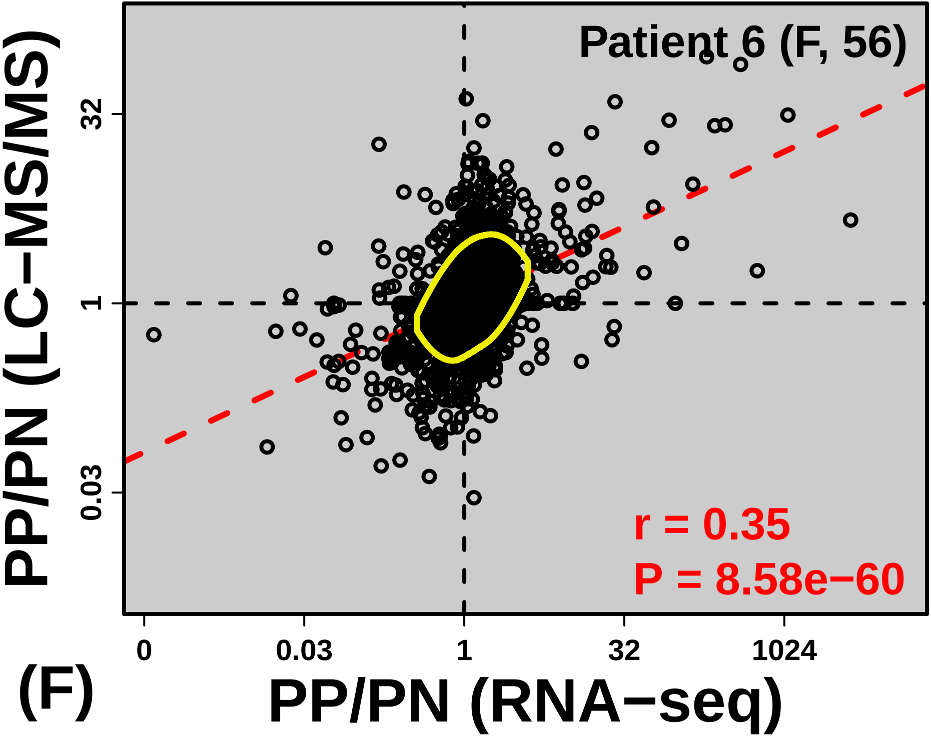
Patient 6 (F, 56)
r = 0.35
P = 8.58e−60
PP/PN (LC−MS/MS)
32
1
0.03
0
0.03
1
32
1024
PP/PN (RNA−seq)
(F)

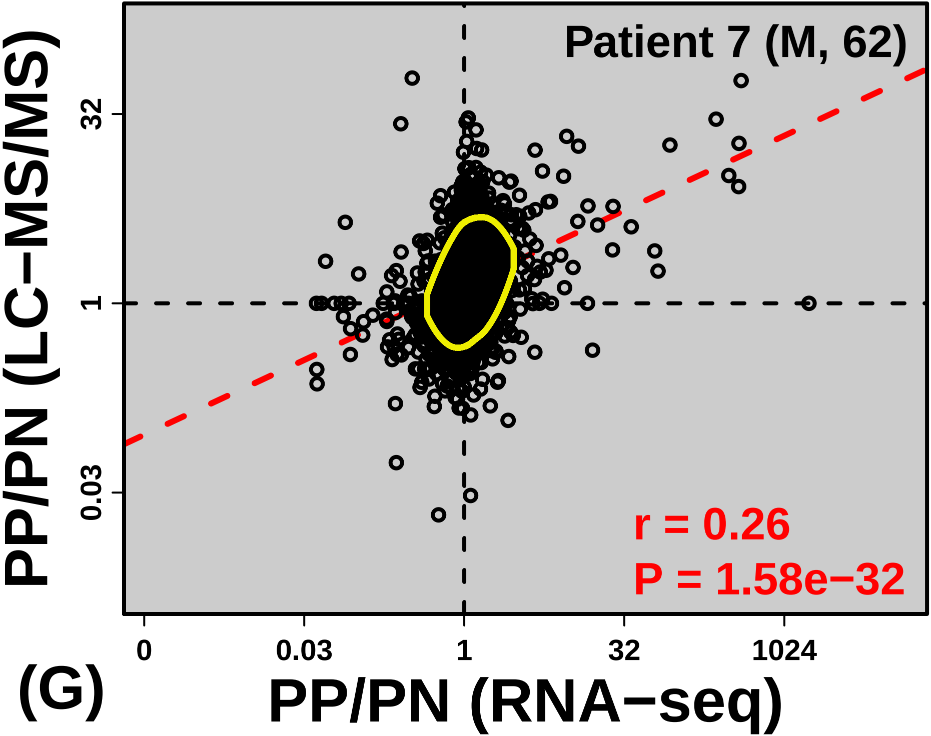

Patient 7 (M, 62)
r = 0.26
P = 1.58e−32
PP/PN (LC−MS/MS)
32
1
0.03
0
0.03
1
32
1024
PP/PN (RNA−seq)

(G)

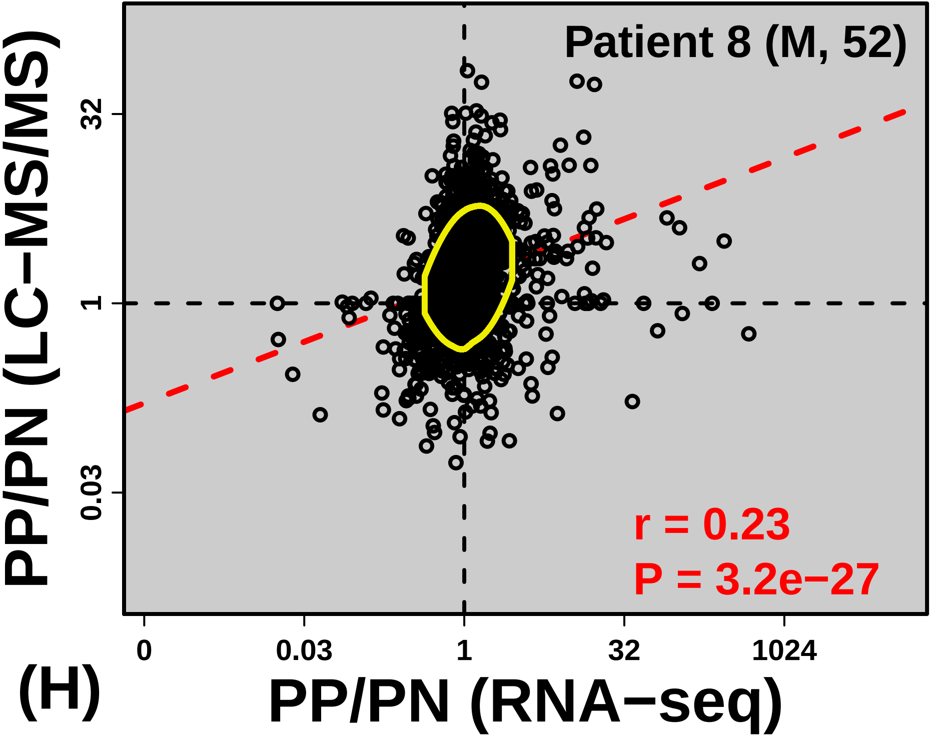

Patient 8 (M, 52)
r = 0.23
P = 3.2e−27
PP/PN (LC−MS/MS)
32
1
0.03
0
0.03
1
32
1024
PP/PN (RNA−seq)

(H)

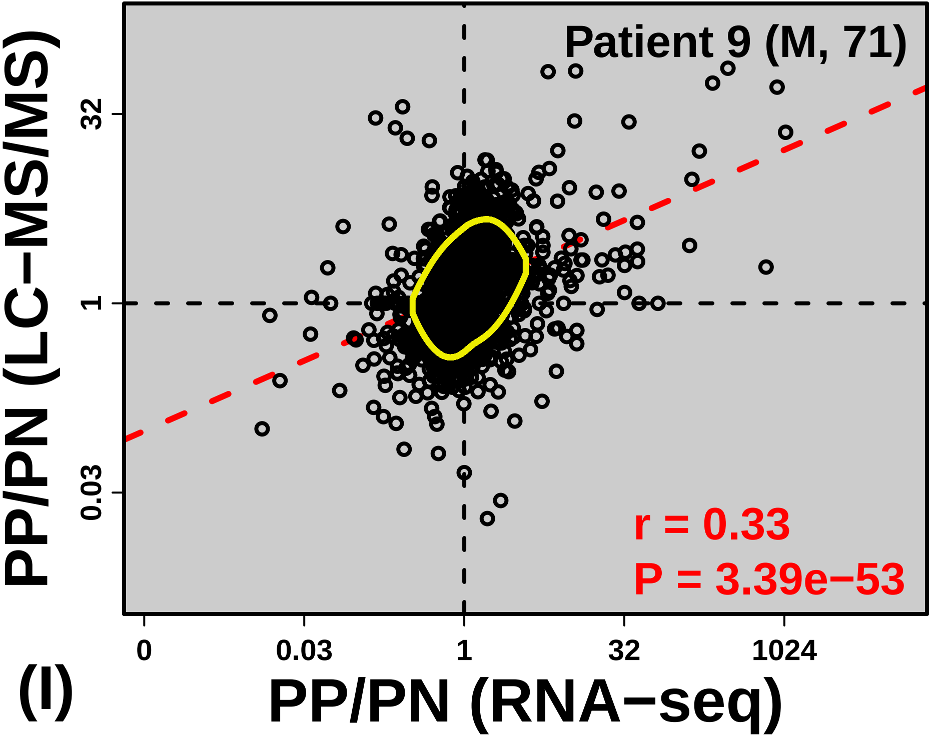

Patient 9 (M, 71)
r = 0.33
P = 3.39e−53
PP/PN (LC−MS/MS)
32
1
0.03
0
0.03
1
32
1024
PP/PN (RNA−seq)

(I)

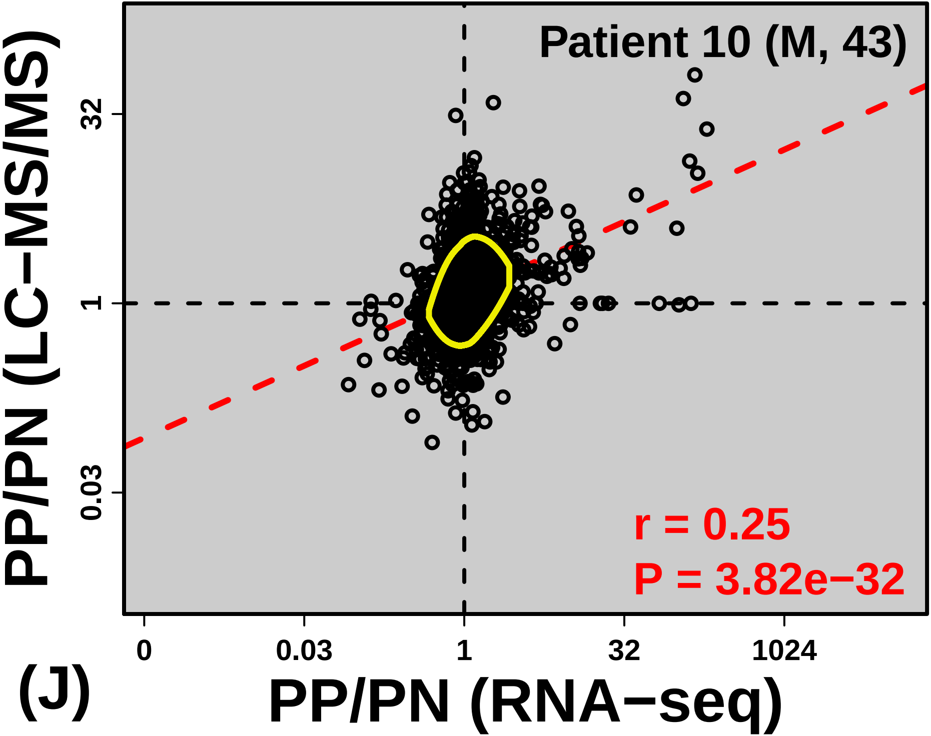

Patient 10 (M, 43)
r = 0.25
P = 3.82e−32
PP/PN (LC−MS/MS)
32
1
0.03
0
0.03
1
32
1024
PP/PN (RNA−seq)

(J)

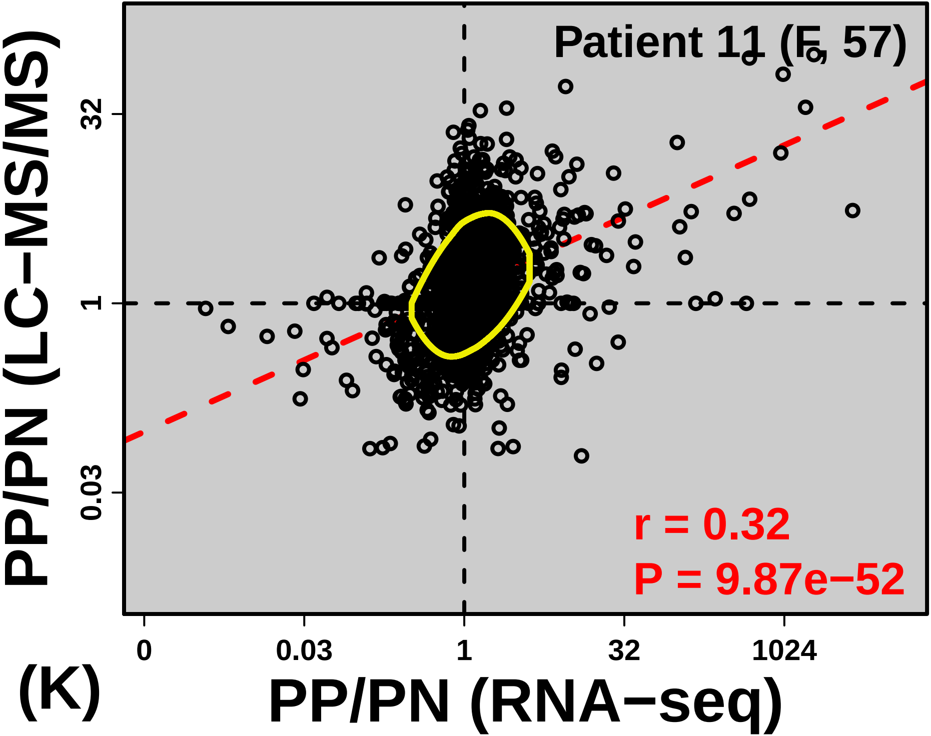
Patient 11 (F, 57)
PP/PN (LC−MS/MS)
32
1
0.03
0
0.03
1
32
1024
r = 0.32
P = 9.87e−52
PP/PN (RNA−seq)

(K)

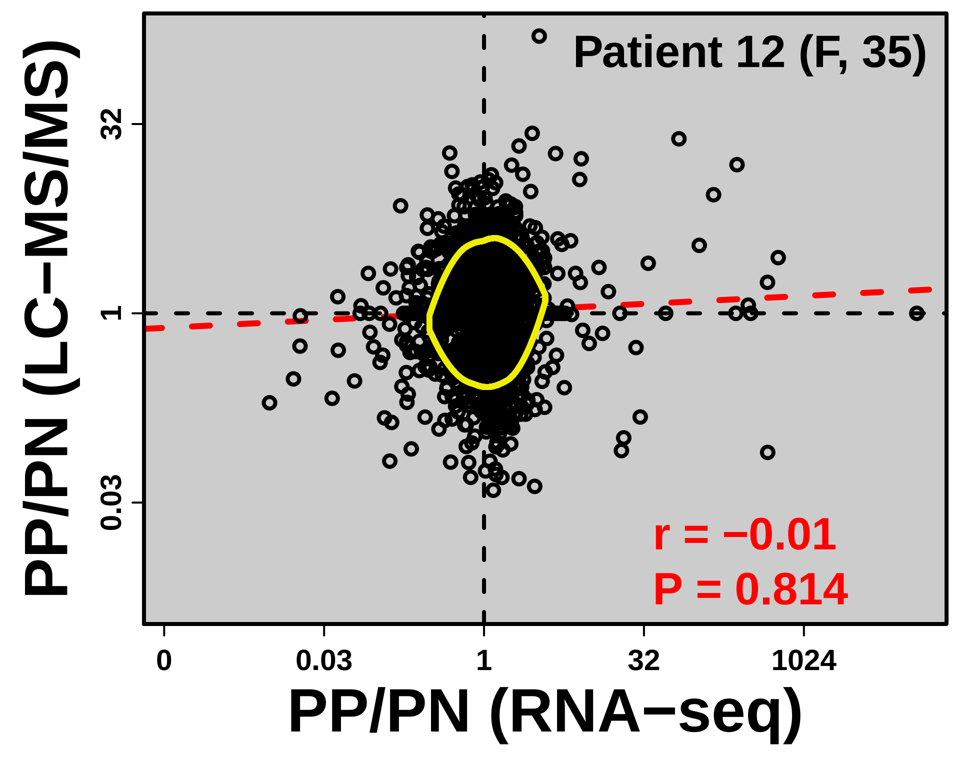
Patient 12 (F, 35)
r = −0.01
P = 0.814
PP/PN (LC−MS/MS)
32
1
0.03
0
0.03
1
32
1024
PP/PN (RNA−seq)

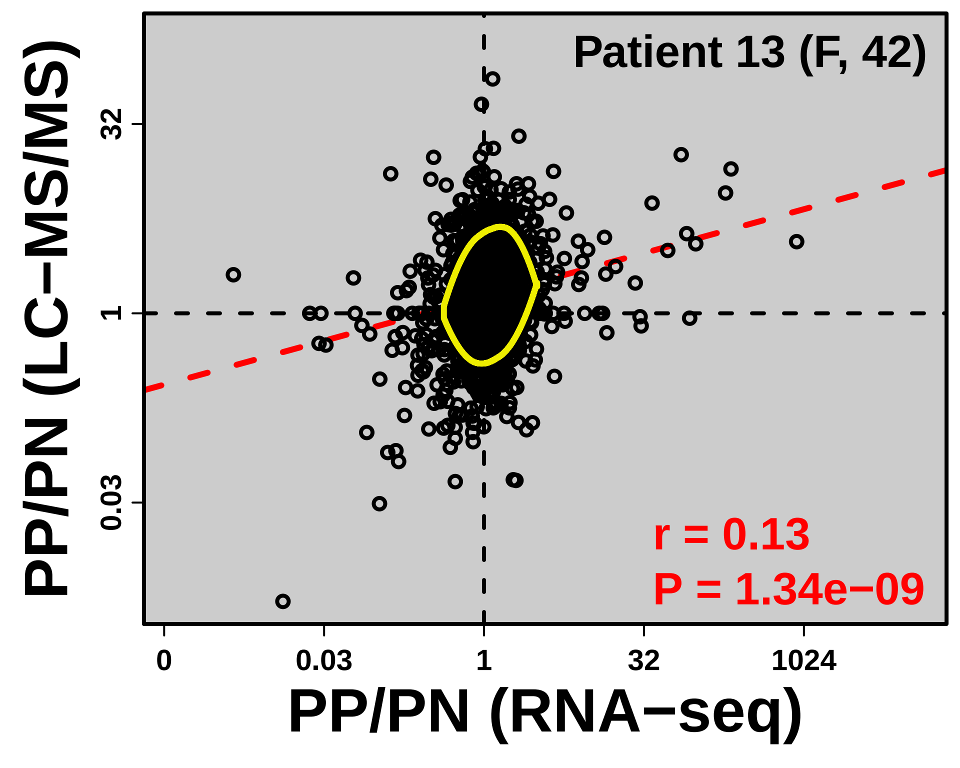

Patient 13 (F, 42)
r = 0.13
P = 1.34e−09
PP/PN (LC−MS/MS)
32
1
0.03
0
0.03
1
32
1024
PP/PN (RNA−seq)

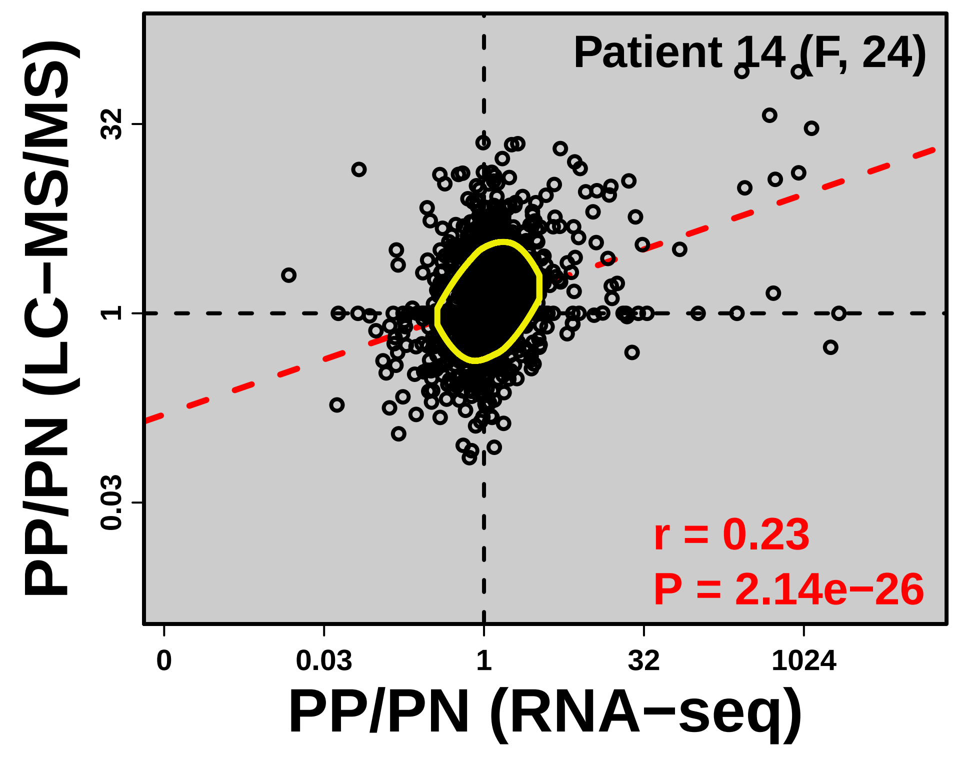
Patient 14 (F, 24)
r = 0.23
P = 2.14e−26
PP/PN (LC−MS/MS)
32
1
0.03
0
0.03
1
32
1024
PP/PN (RNA−seq)
