# Supplementary material for: Proteogenomic analysis of psoriasis reveals discordant and concordant changes in mRNA and protein abundance
Source: Genome Med. 2015 Aug 4;7(1):86. doi: 10.1186/s13073-015-0208-5 (PMC4527112; doi:10.1186/s13073-015-0208-5)
Supplement: Additional file 17: — DEGP enrichment with respect to ordered gene lists from microarray experiments. We screened 2178 ordered gene lists to identify microarray experiments in which PP-increased and PP-decreased DEGPs were disproportionately increased or decreased but in opposite directions. The top-ranked 20 experiments are listed for each of 11 experiment types (a–k; see Additional file 3). Each figure (a–k) shows enrichment statistics for both the 153 PP-increased (left panel) and 56 PP-decreased DEGPs (right panel) with respect to each ordered gene list (described in left margin). Positive enrichment statistics indicate that DEGPs are elevated in the experiment relative to control samples. Negative enrichment statistics indicate that DEGPs are repressed in the experiment relative to control samples. P values are listed in the right margin (Wilcoxon rank sum test). For each experiment, p values were calculated for PP-increased DEGPs and PP-decreased DEGPs, respectively, and experiments are ranked according to the higher of these two p values. (PDF 6387 kb) [file 13073_2015_208_MOESM17_ESM.pdf]

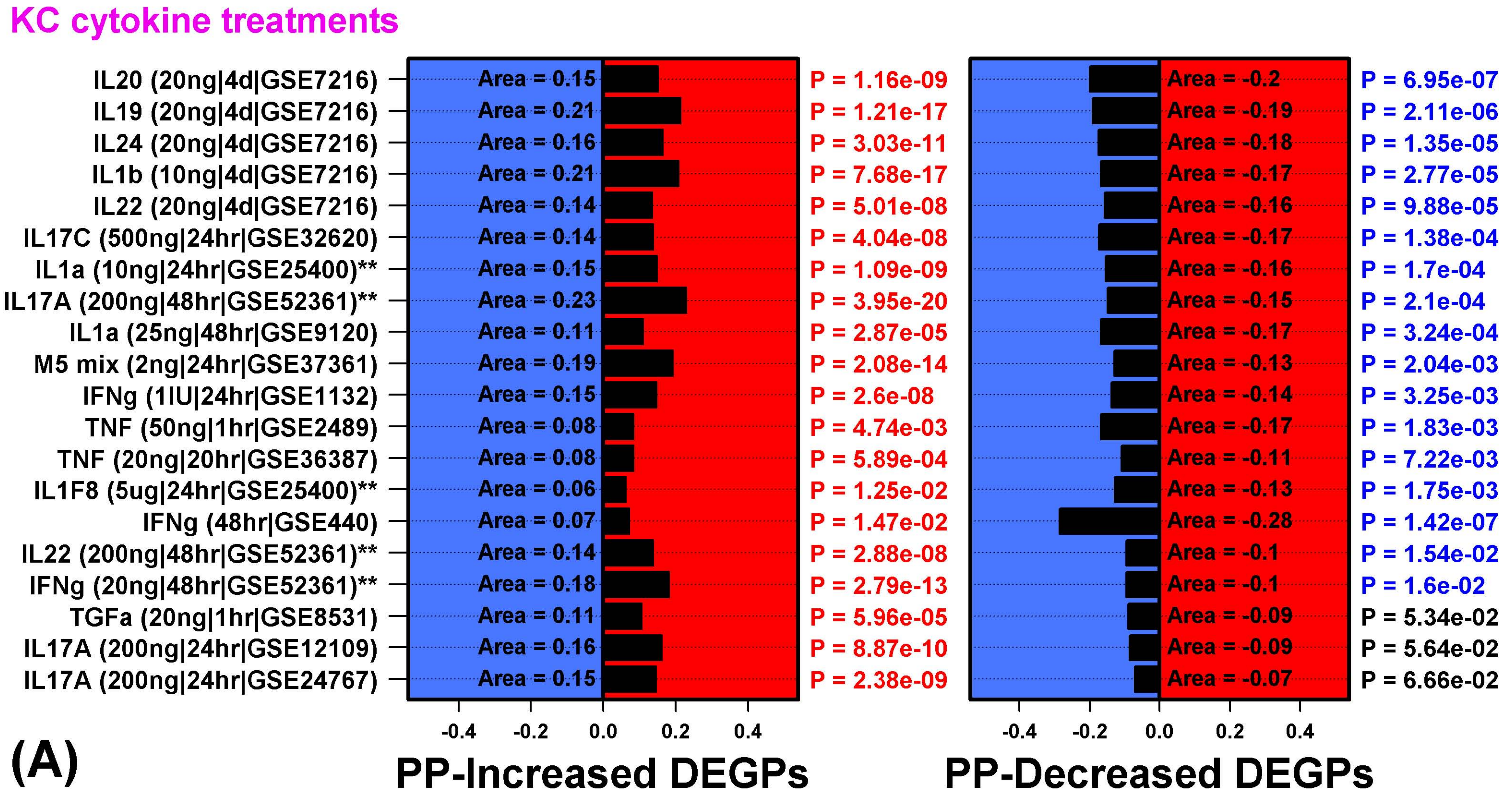
KC cytokine treatments
IL20 (20ng|4d|GSE7216)
IL19 (20ng|4d|GSE7216)
IL24 (20ng|4d|GSE7216)
IL1b (10ng|4d|GSE7216)
IL22 (20ng|4d|GSE7216)
IL17C (500ng|24hr|GSE32620)
IL1a (10ng|24hr|GSE25400)**
IL17A (200ng|48hr|GSE52361)**
IL1a (25ng|48hr|GSE9120)
M5 mix (2ng|24hr|GSE37361)
IFNg (1IU|24hr|GSE1132)
TNF (50ng|1hr|GSE2489)
TNF (20ng|20hr|GSE36387)
IL1F8 (5ug|24hr|GSE25400)**
IFNg (48hr|GSE440)
IL22 (200ng|48hr|GSE52361)**
IFNg (20ng|48hr|GSE52361)**
TGFa (20ng|1hr|GSE8531)
IL17A (200ng|24hr|GSE12109)
IL17A (200ng|24hr|GSE24767)
Area = 0.15
Area = 0.21
Area = 0.16
Area = 0.21
Area = 0.14
Area = 0.14
Area = 0.15
Area = 0.23
Area = 0.11
Area = 0.19
Area = 0.15
Area = 0.08
Area = 0.08
Area = 0.06
Area = 0.07
Area = 0.14
Area = 0.18
Area = 0.11
Area = 0.16
Area = 0.15
P = 1.16e-09
P = 1.21e-17
P = 3.03e-11
P = 7.68e-17
P = 5.01e-08
P = 4.04e-08
P = 1.09e-09
P = 3.95e-20
P = 2.87e-05
P = 2.08e-14
P = 2.6e-08
P = 4.74e-03
P = 5.89e-04
P = 1.25e-02
P = 1.47e-02
P = 2.88e-08
P = 2.79e-13
P = 5.96e-05
P = 8.87e-10
P = 2.38e-09
Area = -0.2
Area = -0.19
Area = -0.18
Area = -0.17
Area = -0.16
Area = -0.17
Area = -0.16
Area = -0.15
Area = -0.17
Area = -0.13
Area = -0.14
Area = -0.17
Area = -0.11
Area = -0.13
Area = -0.28
Area = -0.1
Area = -0.1
Area = -0.09
Area = -0.09
Area = -0.07
P = 6.95e-07
P = 2.11e-06
P = 1.35e-05
P = 2.77e-05
P = 9.88e-05
P = 1.38e-04
P = 1.7e-04
P = 2.1e-04
P = 3.24e-04
P = 2.04e-03
P = 3.25e-03
P = 1.83e-03
P = 7.22e-03
P = 1.75e-03
P = 1.42e-07
P = 1.54e-02
P = 1.6e-02
P = 5.34e-02
P = 5.64e-02
P = 6.66e-02
-0.4
-0.2
0.0
0.2
0.4
-0.4
-0.2
0.0
0.2
0.4
(A)
PP-Increased DEGPs
PP-Decreased DEGPs

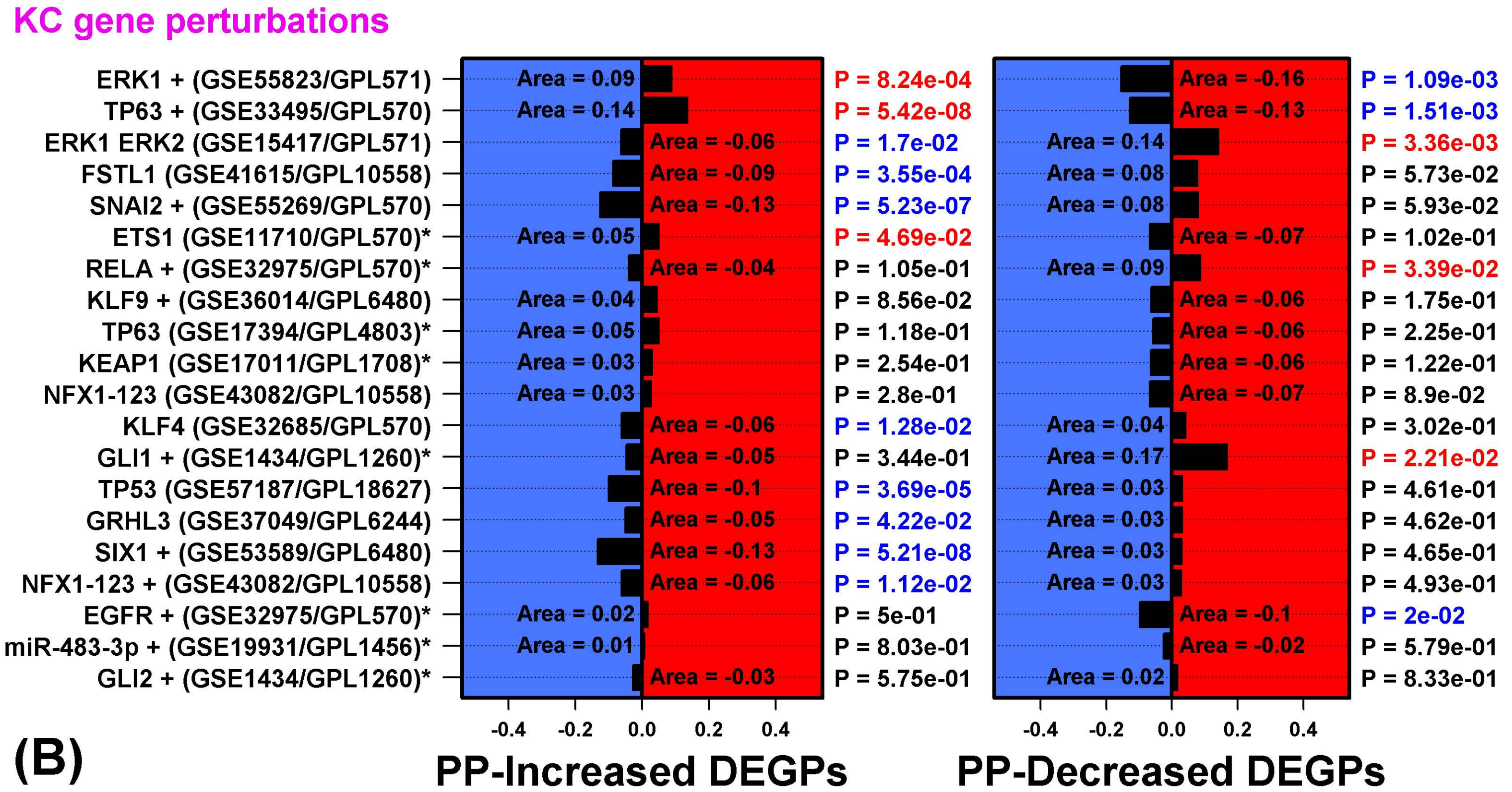

KC gene perturbations
ERK1 + (GSE55823/GPL571)
TP63 + (GSE33495/GPL570)
ERK1 ERK2 (GSE15417/GPL571)
FSTL1 (GSE41615/GPL10558)
SNAI2 + (GSE55269/GPL570)
ETS1 (GSE11710/GPL570)*
RELA + (GSE32975/GPL570)*
KLF9 + (GSE36014/GPL6480)
TP63 (GSE17394/GPL4803)*
KEAP1 (GSE17011/GPL1708)*
NFX1-123 (GSE43082/GPL10558)
KLF4 (GSE32685/GPL570)
GLI1 + (GSE1434/GPL1260)*
TP53 (GSE57187/GPL18627)
GRHL3 (GSE37049/GPL6244)
SIX1 + (GSE53589/GPL6480)
NFX1-123 + (GSE43082/GPL10558)
EGFR + (GSE32975/GPL570)*
miR-483-3p + (GSE19931/GPL1456)*
GLI2 + (GSE1434/GPL1260)*
Area = 0.09
Area = 0.14
Area = -0.06
Area = -0.09
Area = -0.13
Area = 0.05
Area = -0.04
Area = 0.04
Area = 0.05
Area = 0.03
Area = 0.03
Area = -0.06
Area = -0.05
Area = -0.1
Area = -0.05
Area = -0.13
Area = -0.06
Area = 0.02
Area = 0.01
Area = -0.03
P = 8.24e-04
P = 5.42e-08
P = 1.7e-02
P = 3.55e-04
P = 5.23e-07
P = 4.69e-02
P = 1.05e-01
P = 8.56e-02
P = 1.18e-01
P = 2.54e-01
P = 2.8e-01
P = 1.28e-02
P = 3.44e-01
P = 3.69e-05
P = 4.22e-02
P = 5.21e-08
P = 1.12e-02
P = 5e-01
P = 8.03e-01
P = 5.75e-01
-0.4 -0.2 0.0 0.2 0.4
PP-Increased DEGPs
Area = -0.16
Area = -0.13
Area = 0.14
Area = 0.08
Area = 0.08
Area = -0.07
Area = 0.09
Area = -0.06
Area = -0.06
Area = -0.06
Area = -0.07
Area = 0.04
Area = 0.17
Area = 0.03
Area = 0.03
Area = 0.03
Area = 0.03
Area = -0.1
Area = -0.02
Area = 0.02
P = 1.09e-03
P = 1.51e-03
P = 3.36e-03
P = 5.73e-02
P = 5.93e-02
P = 1.02e-01
P = 3.39e-02
P = 1.75e-01
P = 2.25e-01
P = 1.22e-01
P = 8.9e-02
P = 3.02e-01
P = 2.21e-02
P = 4.61e-01
P = 4.62e-01
P = 4.65e-01
P = 4.93e-01
P = 2e-02
P = 5.79e-01
P = 8.33e-01
-0.4 -0.2 0.0 0.2 0.4
PP-Decreased DEGPs
(B)

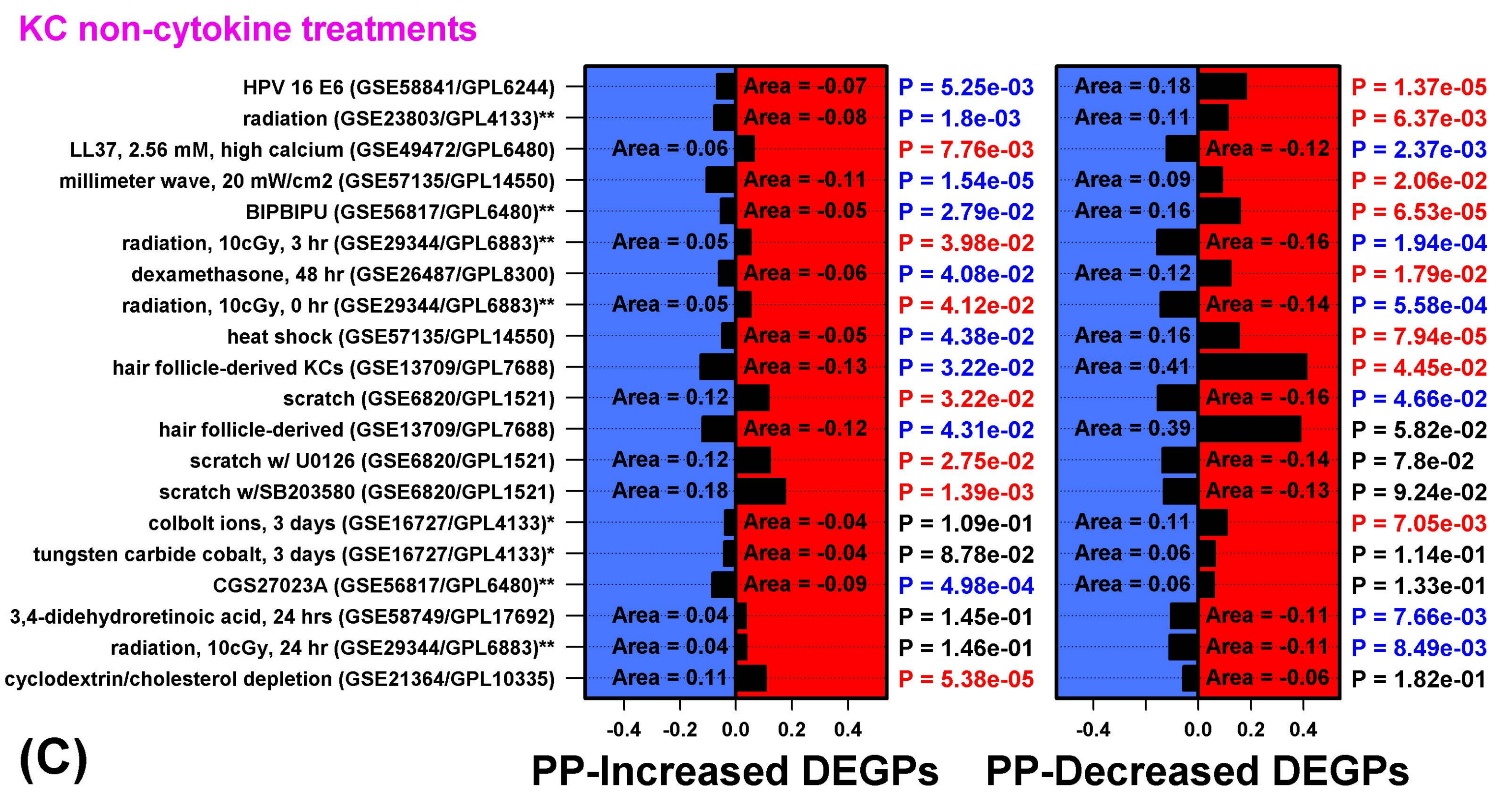
KC non-cytokine treatments
HPV 16 E6 (GSE58841/GPL6244)
radiation (GSE23803/GPL4133)**
LL37, 2.56 mM, high calcium (GSE49472/GPL6480)
millimeter wave, 20 mW/cm2 (GSE57135/GPL14550)
BIPBIPU (GSE56817/GPL6480)**
radiation, 10cGy, 3 hr (GSE29344/GPL6883)**
dexamethasone, 48 hr (GSE26487/GPL8300)
radiation, 10cGy, 0 hr (GSE29344/GPL6883)**
heat shock (GSE57135/GPL14550)
hair follicle-derived KCs (GSE13709/GPL7688)
scratch (GSE6820/GPL1521)
hair follicle-derived (GSE13709/GPL7688)
scratch w/ U0126 (GSE6820/GPL1521)
scratch w/SB203580 (GSE6820/GPL1521)
colbolt ions, 3 days (GSE16727/GPL4133)*
tungsten carbide cobalt, 3 days (GSE16727/GPL4133)*
CGS27023A (GSE56817/GPL6480)**
3,4-didehydroretinoic acid, 24 hrs (GSE58749/GPL17692)
radiation, 10cGy, 24 hr (GSE29344/GPL6883)**
cyclodextrin/cholesterol depletion (GSE21364/GPL10335)
Area = -0.07
P = 5.25e-03
Area = -0.08
P = 1.8e-03
Area = 0.06
P = 7.76e-03
Area = -0.11
P = 1.54e-05
Area = -0.05
P = 2.79e-02
Area = 0.05
P = 3.98e-02
Area = -0.06
P = 4.08e-02
Area = 0.05
P = 4.12e-02
Area = -0.05
P = 4.38e-02
Area = -0.13
P = 3.22e-02
Area = 0.12
P = 3.22e-02
Area = -0.12
P = 4.31e-02
Area = 0.12
P = 2.75e-02
Area = 0.18
P = 1.39e-03
Area = -0.04
P = 1.09e-01
Area = -0.04
P = 8.78e-02
Area = -0.09
P = 4.98e-04
Area = 0.04
P = 1.45e-01
Area = 0.04
P = 1.46e-01
Area = 0.11
P = 5.38e-05
Area = 0.18
P = 1.37e-05
Area = 0.11
P = 6.37e-03
Area = -0.12
P = 2.37e-03
Area = 0.09
P = 2.06e-02
Area = 0.16
P = 6.53e-05
Area = -0.16
P = 1.94e-04
Area = 0.12
P = 1.79e-02
Area = -0.14
P = 5.58e-04
Area = 0.16
P = 7.94e-05
Area = 0.41
P = 4.45e-02
Area = -0.16
P = 4.66e-02
Area = 0.39
P = 5.82e-02
Area = -0.14
P = 7.8e-02
Area = -0.13
P = 9.24e-02
Area = 0.11
P = 7.05e-03
Area = 0.06
P = 1.14e-01
Area = 0.06
P = 1.33e-01
Area = -0.11
P = 7.66e-03
Area = -0.11
P = 8.49e-03
Area = -0.06
P = 1.82e-01
-0.4 -0.2 0.0 0.2 0.4
-0.4 0.0 0.2 0.4
(C)
PP-Increased DEGPs
PP-Decreased DEGPs

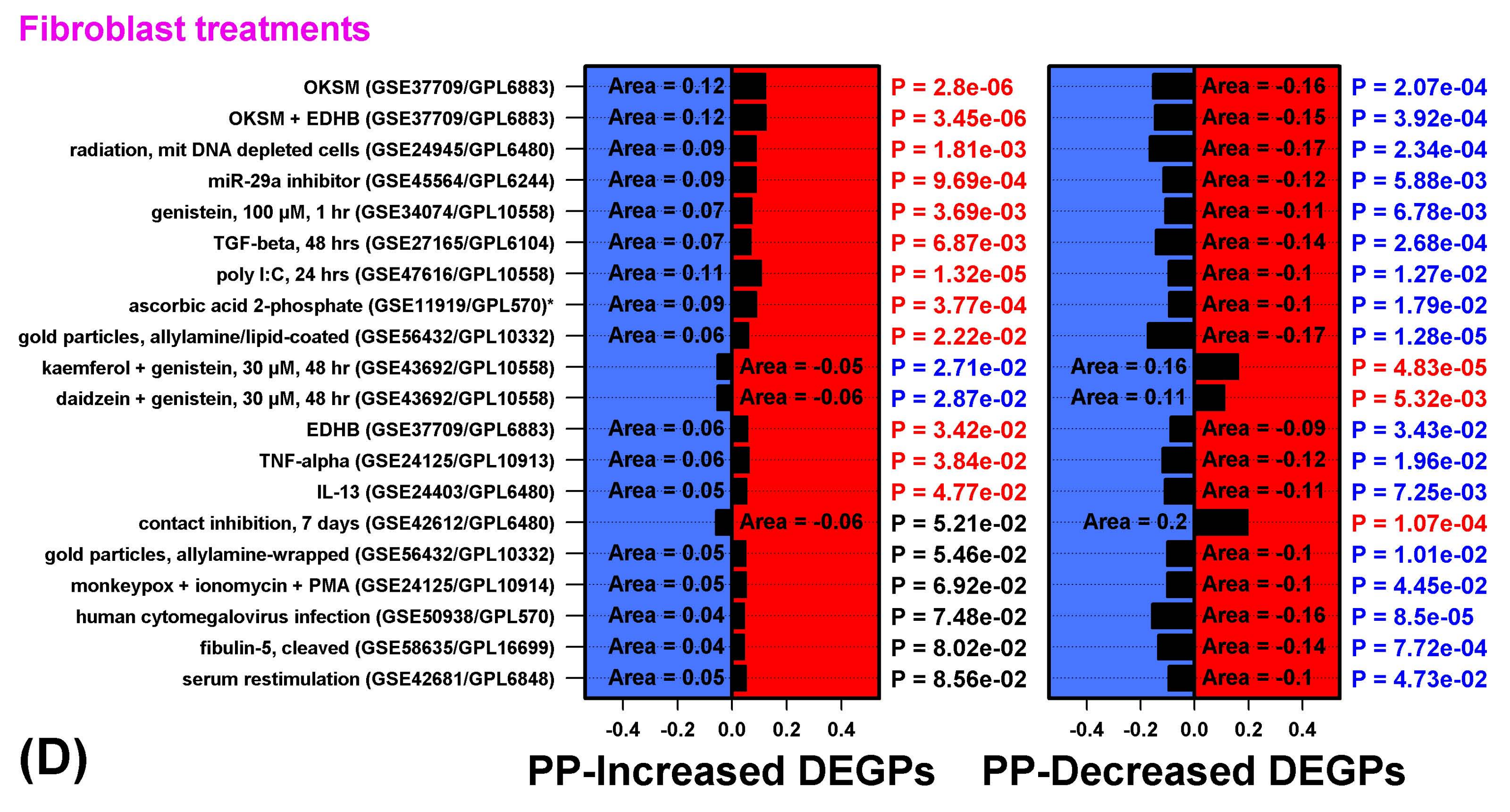

Fibroblast treatments
OKSM (GSE37709/GPL6883)
OKSM + EDHB (GSE37709/GPL6883)
radiation, mit DNA depleted cells (GSE24945/GPL6480)
miR-29a inhibitor (GSE45564/GPL6244)
genistein, 100 μM, 1 hr (GSE34074/GPL10558)
TGF-beta, 48 hrs (GSE27165/GPL6104)
poly I:C, 24 hrs (GSE47616/GPL10558)
ascorbic acid 2-phosphate (GSE11919/GPL570)*
gold particles, allylamine/lipid-coated (GSE56432/GPL10332)
kaemferol + genistein, 30 μM, 48 hr (GSE43692/GPL10558)
daidzein + genistein, 30 μM, 48 hr (GSE43692/GPL10558)
EDHB (GSE37709/GPL6883)
TNF-alpha (GSE24125/GPL10913)
IL-13 (GSE24403/GPL6480)
contact inhibition, 7 days (GSE42612/GPL6480)
gold particles, allylamine-wrapped (GSE56432/GPL10332)
monkeypox + ionomycin + PMA (GSE24125/GPL10914)
human cytomegalovirus infection (GSE50938/GPL570)
fibulin-5, cleaved (GSE58635/GPL16699)
serum restimulation (GSE42681/GPL6848)
Area = 0.12
P = 2.8e-06
Area = 0.12
P = 3.45e-06
Area = 0.09
P = 1.81e-03
Area = 0.09
P = 9.69e-04
Area = 0.07
P = 3.69e-03
Area = 0.07
P = 6.87e-03
Area = 0.11
P = 1.32e-05
Area = 0.09
P = 3.77e-04
Area = 0.06
P = 2.22e-02
Area = -0.05
P = 2.71e-02
Area = -0.06
P = 2.87e-02
Area = 0.06
P = 3.42e-02
Area = 0.06
P = 3.84e-02
Area = 0.05
P = 4.77e-02
Area = -0.06
P = 5.21e-02
Area = 0.05
P = 5.46e-02
Area = 0.05
P = 6.92e-02
Area = 0.04
P = 7.48e-02
Area = 0.04
P = 8.02e-02
Area = 0.05
P = 8.56e-02
Area = -0.16
P = 2.07e-04
Area = -0.15
P = 3.92e-04
Area = -0.17
P = 2.34e-04
Area = -0.12
P = 5.88e-03
Area = -0.11
P = 6.78e-03
Area = -0.14
P = 2.68e-04
Area = -0.1
P = 1.27e-02
Area = -0.1
P = 1.79e-02
Area = -0.17
P = 1.28e-05
Area = 0.16
P = 4.83e-05
Area = 0.11
P = 5.32e-03
Area = -0.09
P = 3.43e-02
Area = -0.12
P = 1.96e-02
Area = -0.11
P = 7.25e-03
Area = 0.2
P = 1.07e-04
Area = -0.1
P = 1.01e-02
Area = -0.1
P = 4.45e-02
Area = -0.16
P = 8.5e-05
Area = -0.14
P = 7.72e-04
Area = -0.1
P = 4.73e-02
-0.4 -0.2 0.0 0.2 0.4
-0.4 -0.2 0.0 0.2 0.4
(D)
PP-Increased DEGPs
PP-Decreased DEGPs

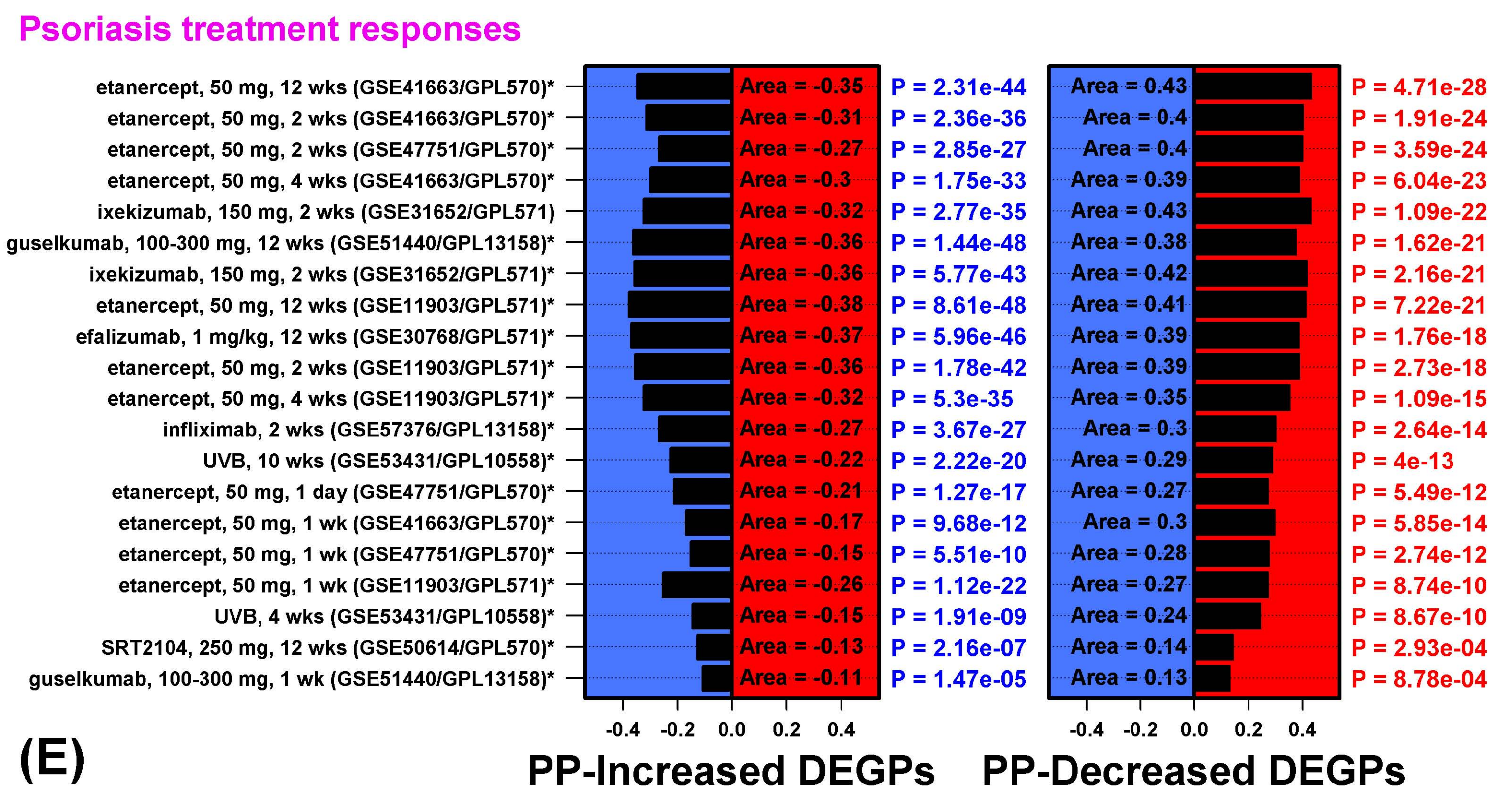

Psoriasis treatment responses
etanercept, 50 mg, 12 wks (GSE41663/GPL570)*
etanercept, 50 mg, 2 wks (GSE41663/GPL570)*
etanercept, 50 mg, 2 wks (GSE47751/GPL570)*
etanercept, 50 mg, 4 wks (GSE41663/GPL570)*
ixekizumab, 150 mg, 2 wks (GSE31652/GPL571)
guselkumab, 100-300 mg, 12 wks (GSE51440/GPL13158)*
ixekizumab, 150 mg, 2 wks (GSE31652/GPL571)*
etanercept, 50 mg, 12 wks (GSE11903/GPL571)*
efalizumab, 1 mg/kg, 12 wks (GSE30768/GPL571)*
etanercept, 50 mg, 2 wks (GSE11903/GPL571)*
etanercept, 50 mg, 4 wks (GSE11903/GPL571)*
infliximab, 2 wks (GSE57376/GPL13158)*
UVB, 10 wks (GSE53431/GPL10558)*
etanercept, 50 mg, 1 day (GSE47751/GPL570)*
etanercept, 50 mg, 1 wk (GSE41663/GPL570)*
etanercept, 50 mg, 1 wk (GSE47751/GPL570)*
etanercept, 50 mg, 1 wk (GSE11903/GPL571)*
UVB, 4 wks (GSE53431/GPL10558)*
SRT2104, 250 mg, 12 wks (GSE50614/GPL570)*
guselkumab, 100-300 mg, 1 wk (GSE51440/GPL13158)*
Area = -0.35
Area = -0.31
Area = -0.27
Area = -0.3
Area = -0.32
Area = -0.36
Area = -0.36
Area = -0.38
Area = -0.37
Area = -0.36
Area = -0.32
Area = -0.27
Area = -0.22
Area = -0.21
Area = -0.17
Area = -0.15
Area = -0.26
Area = -0.15
Area = -0.13
Area = -0.11
P = 2.31e-44
P = 2.36e-36
P = 2.85e-27
P = 1.75e-33
P = 2.77e-35
P = 1.44e-48
P = 5.77e-43
P = 8.61e-48
P = 5.96e-46
P = 1.78e-42
P = 5.3e-35
P = 3.67e-27
P = 2.22e-20
P = 1.27e-17
P = 9.68e-12
P = 5.51e-10
P = 1.12e-22
P = 1.91e-09
P = 2.16e-07
P = 1.47e-05
Area = 0.43
Area = 0.4
Area = 0.4
Area = 0.39
Area = 0.43
Area = 0.38
Area = 0.42
Area = 0.41
Area = 0.39
Area = 0.39
Area = 0.35
Area = 0.3
Area = 0.29
Area = 0.27
Area = 0.3
Area = 0.28
Area = 0.27
Area = 0.24
Area = 0.14
Area = 0.13
P = 4.71e-28
P = 1.91e-24
P = 3.59e-24
P = 6.04e-23
P = 1.09e-22
P = 1.62e-21
P = 2.16e-21
P = 7.22e-21
P = 1.76e-18
P = 2.73e-18
P = 1.09e-15
P = 2.64e-14
P = 4e-13
P = 5.49e-12
P = 5.85e-14
P = 2.74e-12
P = 8.74e-10
P = 8.67e-10
P = 2.93e-04
P = 8.78e-04
-0.4 -0.2 0.0 0.2 0.4
-0.4 -0.2 0.0 0.2 0.4
(E)
PP-Increased DEGPs
PP-Decreased DEGPs

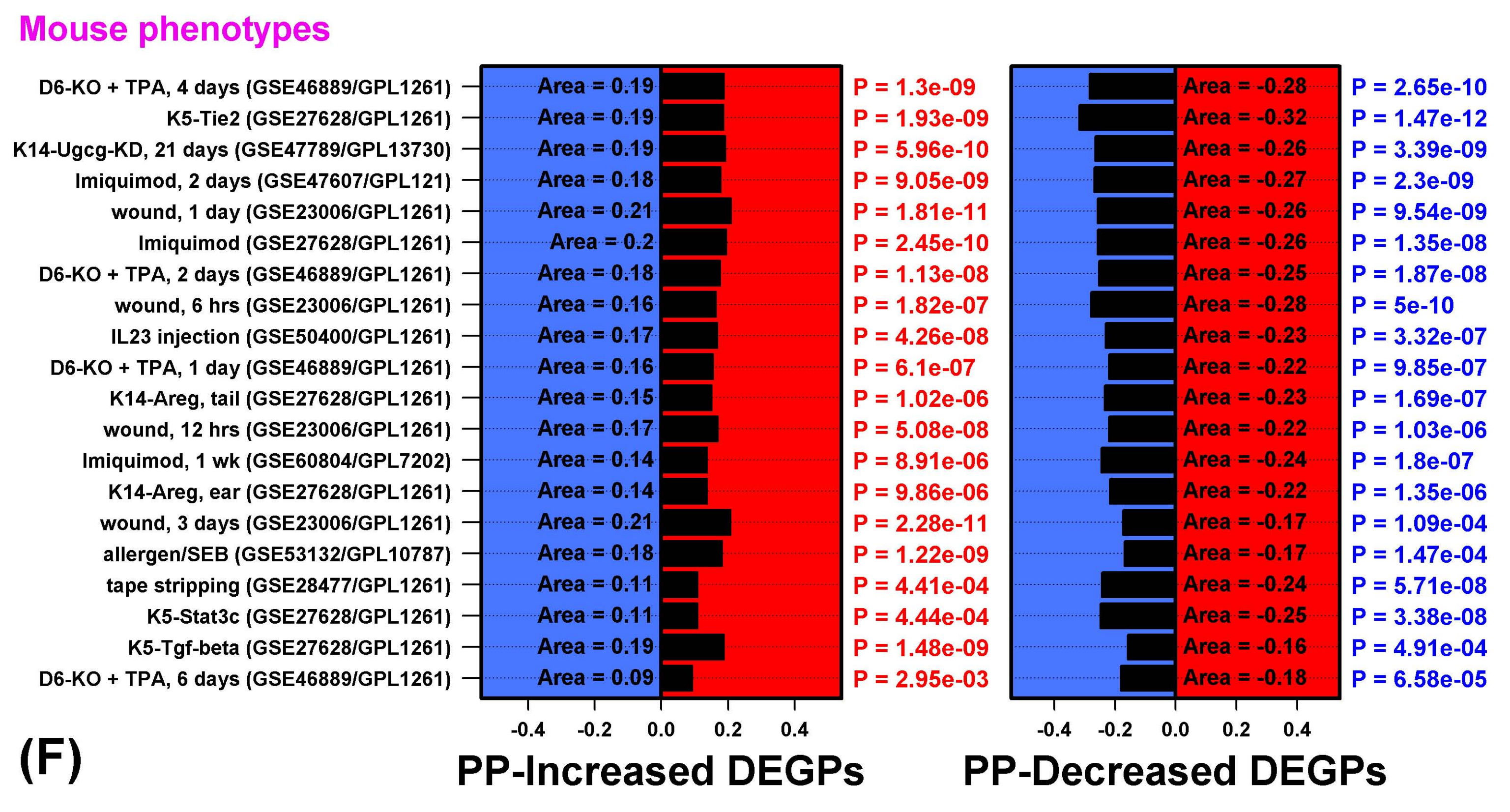

Mouse phenotypes
D6-KO + TPA, 4 days (GSE46889/GPL1261)
K5-Tie2 (GSE27628/GPL1261)
K14-Ugcg-KD, 21 days (GSE47789/GPL13730)
Imiquimod, 2 days (GSE47607/GPL121)
wound, 1 day (GSE23006/GPL1261)
Imiquimod (GSE27628/GPL1261)
D6-KO + TPA, 2 days (GSE46889/GPL1261)
wound, 6 hrs (GSE23006/GPL1261)
IL23 injection (GSE50400/GPL1261)
D6-KO + TPA, 1 day (GSE46889/GPL1261)
K14-Areg, tail (GSE27628/GPL1261)
wound, 12 hrs (GSE23006/GPL1261)
Imiquimod, 1 wk (GSE60804/GPL7202)
K14-Areg, ear (GSE27628/GPL1261)
wound, 3 days (GSE23006/GPL1261)
allergen/SEB (GSE53132/GPL10787)
tape stripping (GSE28477/GPL1261)
K5-Stat3c (GSE27628/GPL1261)
K5-Tgf-beta (GSE27628/GPL1261)
D6-KO + TPA, 6 days (GSE46889/GPL1261)
Area = 0.19
Area = 0.19
Area = 0.19
Area = 0.18
Area = 0.21
Area = 0.2
Area = 0.18
Area = 0.16
Area = 0.17
Area = 0.16
Area = 0.15
Area = 0.17
Area = 0.14
Area = 0.14
Area = 0.21
Area = 0.18
Area = 0.11
Area = 0.11
Area = 0.19
Area = 0.09
P = 1.3e-09
P = 1.93e-09
P = 5.96e-10
P = 9.05e-09
P = 1.81e-11
P = 2.45e-10
P = 1.13e-08
P = 1.82e-07
P = 4.26e-08
P = 6.1e-07
P = 1.02e-06
P = 5.08e-08
P = 8.91e-06
P = 9.86e-06
P = 2.28e-11
P = 1.22e-09
P = 4.41e-04
P = 4.44e-04
P = 1.48e-09
P = 2.95e-03
Area = -0.28
Area = -0.32
Area = -0.26
Area = -0.27
Area = -0.26
Area = -0.26
Area = -0.25
Area = -0.28
Area = -0.23
Area = -0.22
Area = -0.23
Area = -0.22
Area = -0.24
Area = -0.22
Area = -0.17
Area = -0.17
Area = -0.24
Area = -0.25
Area = -0.16
Area = -0.18
P = 2.65e-10
P = 1.47e-12
P = 3.39e-09
P = 2.3e-09
P = 9.54e-09
P = 1.35e-08
P = 1.87e-08
P = 5e-10
P = 3.32e-07
P = 9.85e-07
P = 1.69e-07
P = 1.03e-06
P = 1.8e-07
P = 1.35e-06
P = 1.09e-04
P = 1.47e-04
P = 5.71e-08
P = 3.38e-08
P = 4.91e-04
P = 6.58e-05
-0.4
-0.2
0.0
0.2
0.4
-0.4
-0.2
0.0
0.2
0.4
PP-Increased DEGPs
PP-Decreased DEGPs
(F)

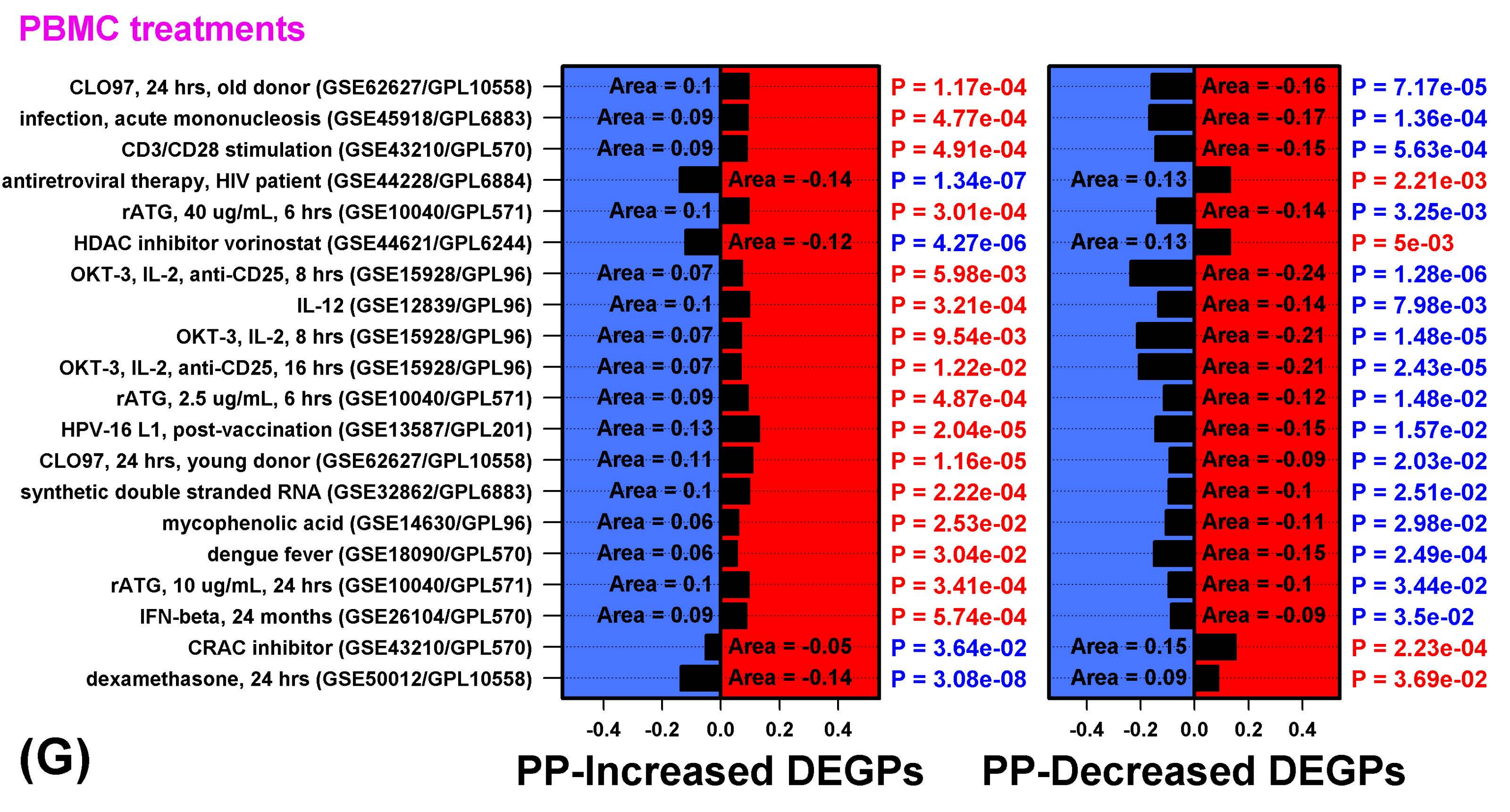
PBMC treatments
CLO97, 24 hrs, old donor (GSE62627/GPL10558)
infection, acute mononucleosis (GSE45918/GPL6883)
CD3/CD28 stimulation (GSE43210/GPL570)
antiretroviral therapy, HIV patient (GSE44228/GPL6884)
rATG, 40 ug/mL, 6 hrs (GSE10040/GPL571)
HDAC inhibitor vorinostat (GSE44621/GPL6244)
OKT-3, IL-2, anti-CD25, 8 hrs (GSE15928/GPL96)
IL-12 (GSE12839/GPL96)
OKT-3, IL-2, 8 hrs (GSE15928/GPL96)
OKT-3, IL-2, anti-CD25, 16 hrs (GSE15928/GPL96)
rATG, 2.5 ug/mL, 6 hrs (GSE10040/GPL571)
HPV-16 L1, post-vaccination (GSE13587/GPL201)
CLO97, 24 hrs, young donor (GSE62627/GPL10558)
synthetic double stranded RNA (GSE32862/GPL6883)
mycophenolic acid (GSE14630/GPL96)
dengue fever (GSE18090/GPL570)
rATG, 10 ug/mL, 24 hrs (GSE10040/GPL571)
IFN-beta, 24 months (GSE26104/GPL570)
CRAC inhibitor (GSE43210/GPL570)
dexamethasone, 24 hrs (GSE50012/GPL10558)
Area = 0.1
Area = 0.09
Area = 0.09
Area = -0.14
Area = 0.1
Area = -0.12
Area = 0.07
Area = 0.1
Area = 0.07
Area = 0.07
Area = 0.09
Area = 0.13
Area = 0.11
Area = 0.1
Area = 0.06
Area = 0.06
Area = 0.1
Area = 0.09
Area = -0.05
Area = -0.14
P = 1.17e-04
P = 4.77e-04
P = 4.91e-04
P = 1.34e-07
P = 3.01e-04
P = 4.27e-06
P = 5.98e-03
P = 3.21e-04
P = 9.54e-03
P = 1.22e-02
P = 4.87e-04
P = 2.04e-05
P = 1.16e-05
P = 2.22e-04
P = 2.53e-02
P = 3.04e-02
P = 3.41e-04
P = 5.74e-04
P = 3.64e-02
P = 3.08e-08
Area = -0.16
Area = -0.17
Area = -0.15
Area = 0.13
Area = -0.14
Area = 0.13
Area = -0.24
Area = -0.14
Area = -0.21
Area = -0.21
Area = -0.12
Area = -0.15
Area = -0.09
Area = -0.1
Area = -0.11
Area = -0.15
Area = -0.1
Area = -0.09
Area = 0.15
Area = 0.09
P = 7.17e-05
P = 1.36e-04
P = 5.63e-04
P = 2.21e-03
P = 3.25e-03
P = 5e-03
P = 1.28e-06
P = 7.98e-03
P = 1.48e-05
P = 2.43e-05
P = 1.48e-02
P = 1.57e-02
P = 2.03e-02
P = 2.51e-02
P = 2.98e-02
P = 2.49e-04
P = 3.44e-02
P = 3.5e-02
P = 2.23e-04
P = 3.69e-02
-0.4
-0.2
0.0
0.2
0.4
-0.4
-0.2
0.0
0.2
0.4
(G)
PP-Increased DEGPs
PP-Decreased DEGPs

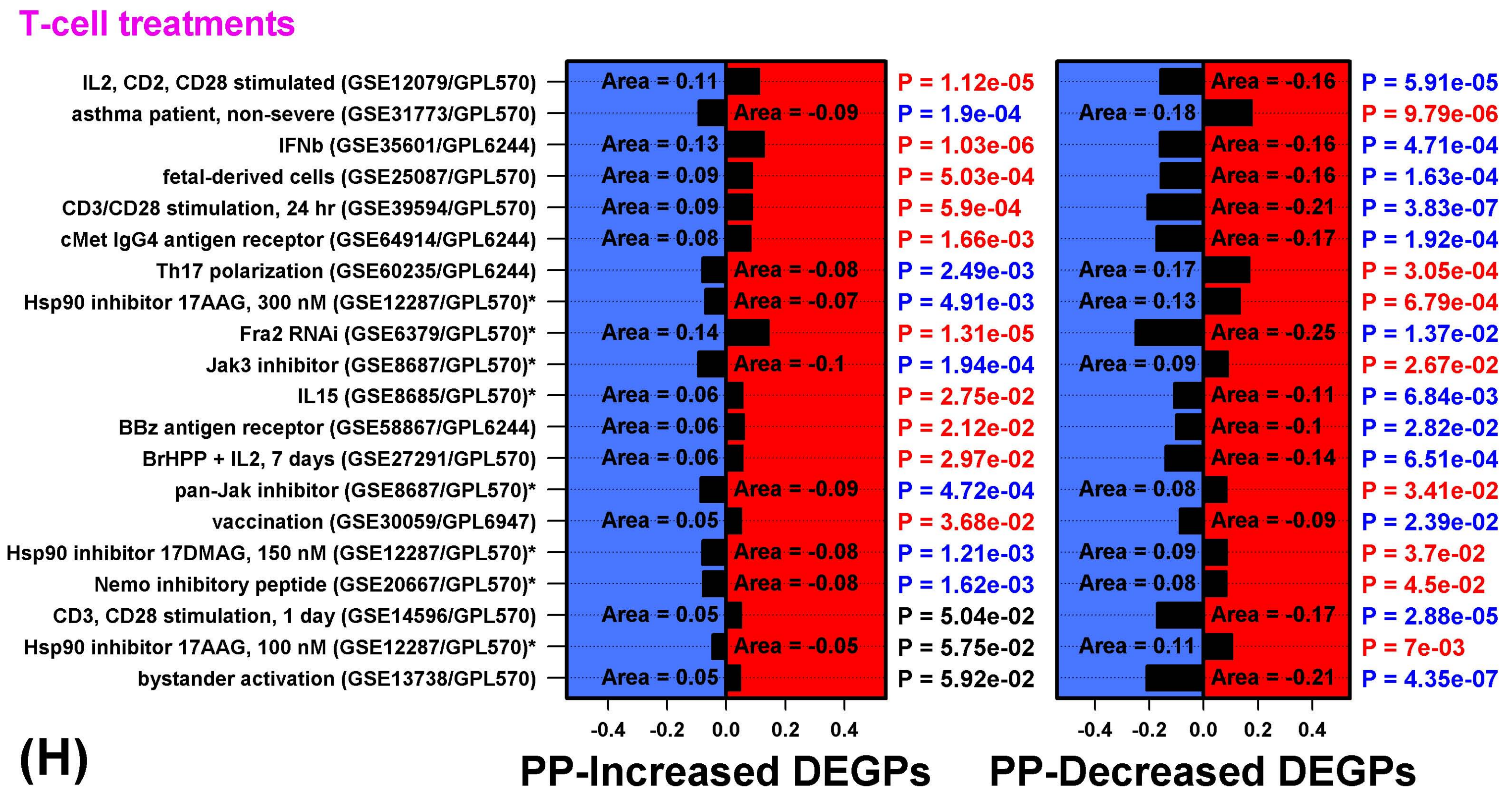
T-cell treatments
IL2, CD2, CD28 stimulated (GSE12079/GPL570)
asthma patient, non-severe (GSE31773/GPL570)
IFNb (GSE35601/GPL6244)
fetal-derived cells (GSE25087/GPL570)
CD3/CD28 stimulation, 24 hr (GSE39594/GPL570)
cMet IgG4 antigen receptor (GSE64914/GPL6244)
Th17 polarization (GSE60235/GPL6244)
Hsp90 inhibitor 17AAG, 300 nM (GSE12287/GPL570)*
Fra2 RNAi (GSE6379/GPL570)*
Jak3 inhibitor (GSE8687/GPL570)*
IL15 (GSE8685/GPL570)*
BBz antigen receptor (GSE58867/GPL6244)
BrHPP + IL2, 7 days (GSE27291/GPL570)
pan-Jak inhibitor (GSE8687/GPL570)*
vaccination (GSE30059/GPL6947)
Hsp90 inhibitor 17DMAG, 150 nM (GSE12287/GPL570)*
Nemo inhibitory peptide (GSE20667/GPL570)*
CD3, CD28 stimulation, 1 day (GSE14596/GPL570)
Hsp90 inhibitor 17AAG, 100 nM (GSE12287/GPL570)*
bystander activation (GSE13738/GPL570)
Area = 0.11
Area = -0.09
Area = 0.13
Area = 0.09
Area = 0.09
Area = 0.08
Area = -0.08
Area = -0.07
Area = 0.14
Area = -0.1
Area = 0.06
Area = 0.06
Area = 0.06
Area = -0.09
Area = 0.05
Area = -0.08
Area = -0.08
Area = 0.05
Area = -0.05
Area = 0.05
P = 1.12e-05
P = 1.9e-04
P = 1.03e-06
P = 5.03e-04
P = 5.9e-04
P = 1.66e-03
P = 2.49e-03
P = 4.91e-03
P = 1.31e-05
P = 1.94e-04
P = 2.75e-02
P = 2.12e-02
P = 2.97e-02
P = 4.72e-04
P = 3.68e-02
P = 1.21e-03
P = 1.62e-03
P = 5.04e-02
P = 5.75e-02
P = 5.92e-02
-0.4 -0.2 0.0 0.2 0.4
PP-Increased DEGPs
Area = -0.16
Area = 0.18
Area = -0.16
Area = -0.16
Area = -0.21
Area = -0.17
Area = 0.17
Area = 0.13
Area = -0.25
Area = 0.09
Area = -0.11
Area = -0.1
Area = -0.14
Area = 0.08
Area = -0.09
Area = 0.09
Area = 0.08
Area = -0.17
Area = 0.11
Area = -0.21
P = 5.91e-05
P = 9.79e-06
P = 4.71e-04
P = 1.63e-04
P = 3.83e-07
P = 1.92e-04
P = 3.05e-04
P = 6.79e-04
P = 1.37e-02
P = 2.67e-02
P = 6.84e-03
P = 2.82e-02
P = 6.51e-04
P = 3.41e-02
P = 2.39e-02
P = 3.7e-02
P = 4.5e-02
P = 2.88e-05
P = 7e-03
P = 4.35e-07
-0.4 -0.2 0.0 0.2 0.4
PP-Decreased DEGPs
(H)

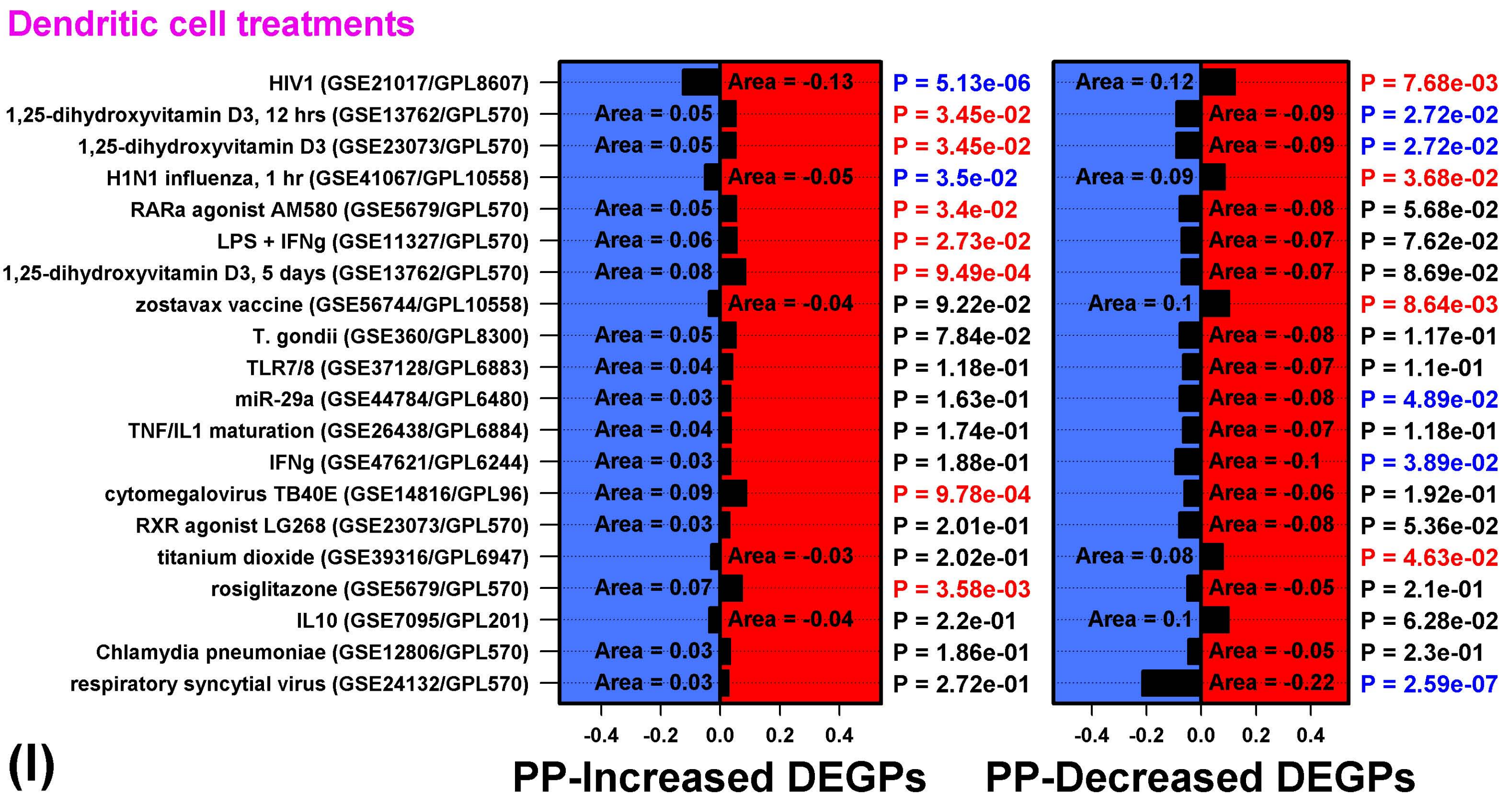

Dendritic cell treatments
HIV1 (GSE21017/GPL8607)
1,25-dihydroxyvitamin D3, 12 hrs (GSE13762/GPL570)
1,25-dihydroxyvitamin D3 (GSE23073/GPL570)
H1N1 influenza, 1 hr (GSE41067/GPL10558)
RARa agonist AM580 (GSE5679/GPL570)
LPS + IFNg (GSE11327/GPL570)
1,25-dihydroxyvitamin D3, 5 days (GSE13762/GPL570)
zostavax vaccine (GSE56744/GPL10558)
T. gondii (GSE360/GPL8300)
TLR7/8 (GSE37128/GPL6883)
miR-29a (GSE44784/GPL6480)
TNF/IL1 maturation (GSE26438/GPL6884)
IFNg (GSE47621/GPL6244)
cytomegalovirus TB40E (GSE14816/GPL96)
RXR agonist LG268 (GSE23073/GPL570)
titanium dioxide (GSE39316/GPL6947)
rosiglitazone (GSE5679/GPL570)
IL10 (GSE7095/GPL201)
Chlamydia pneumoniae (GSE12806/GPL570)
respiratory syncytial virus (GSE24132/GPL570)
Area = -0.13
Area = 0.05
Area = 0.05
Area = -0.05
Area = 0.05
Area = 0.06
Area = 0.08
Area = -0.04
Area = 0.05
Area = 0.04
Area = 0.03
Area = 0.04
Area = 0.03
Area = 0.09
Area = 0.03
Area = -0.03
Area = 0.07
Area = -0.04
Area = 0.03
Area = 0.03
P = 5.13e-06
P = 3.45e-02
P = 3.45e-02
P = 3.5e-02
P = 3.4e-02
P = 2.73e-02
P = 9.49e-04
P = 9.22e-02
P = 7.84e-02
P = 1.18e-01
P = 1.63e-01
P = 1.74e-01
P = 1.88e-01
P = 9.78e-04
P = 2.01e-01
P = 2.02e-01
P = 3.58e-03
P = 2.2e-01
P = 1.86e-01
P = 2.72e-01
-0.4 -0.2 0.0 0.2 0.4
PP-Increased DEGPs
Area = 0.12
Area = -0.09
Area = -0.09
Area = 0.09
Area = -0.08
Area = -0.07
Area = -0.07
Area = 0.1
Area = -0.08
Area = -0.07
Area = -0.08
Area = -0.07
Area = -0.1
Area = -0.06
Area = -0.08
Area = 0.08
Area = -0.05
Area = 0.1
Area = -0.05
Area = -0.22
P = 7.68e-03
P = 2.72e-02
P = 2.72e-02
P = 3.68e-02
P = 5.68e-02
P = 7.62e-02
P = 8.69e-02
P = 8.64e-03
P = 1.17e-01
P = 1.1e-01
P = 4.89e-02
P = 1.18e-01
P = 3.89e-02
P = 1.92e-01
P = 5.36e-02
P = 4.63e-02
P = 2.1e-01
P = 6.28e-02
P = 2.3e-01
P = 2.59e-07
-0.4 -0.2 0.0 0.2 0.4
PP-Decreased DEGPs
(I)

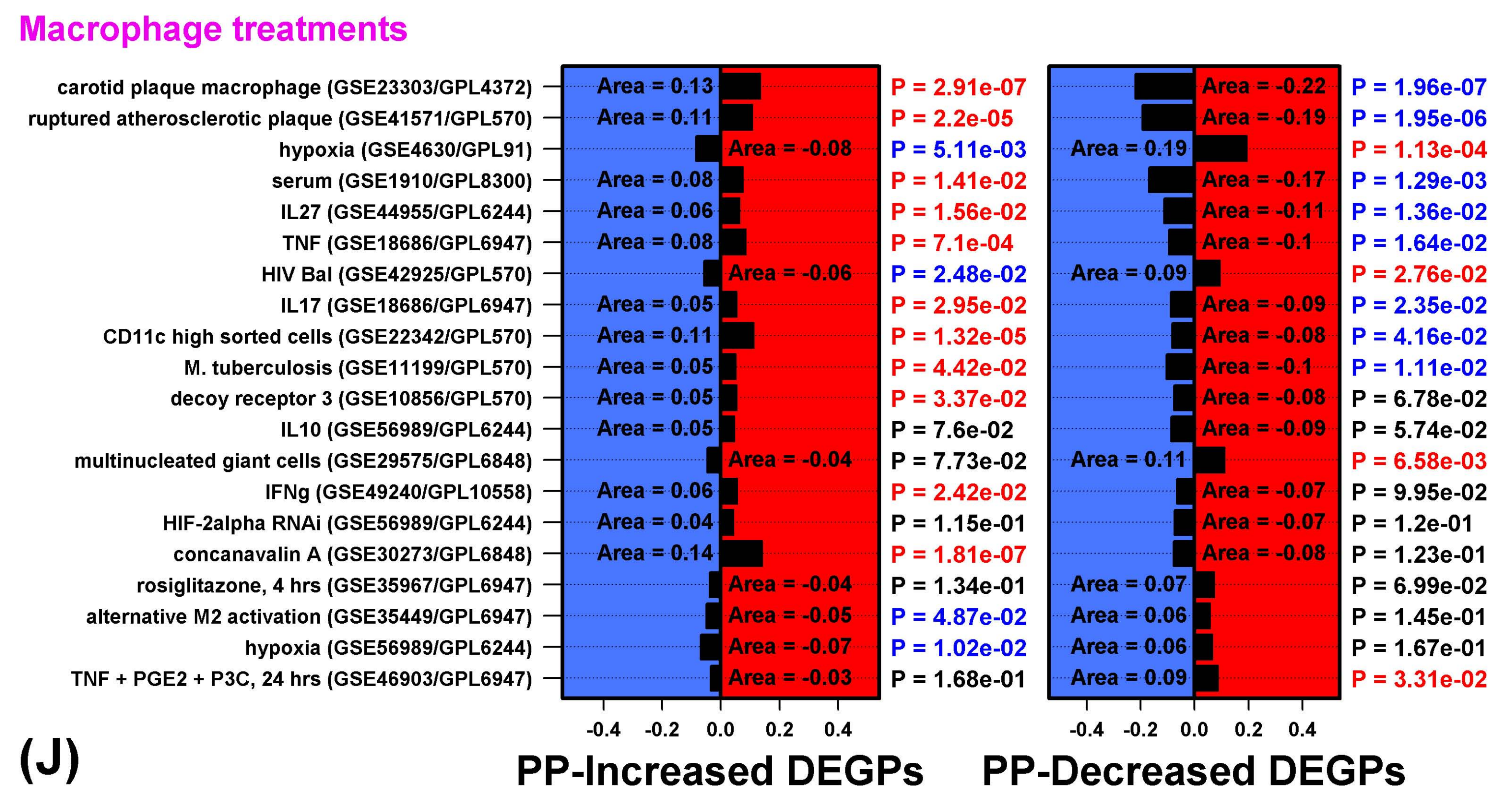
Macrophage treatments
carotid plaque macrophage (GSE23303/GPL4372)
ruptured atherosclerotic plaque (GSE41571/GPL570)
hypoxia (GSE4630/GPL91)
serum (GSE1910/GPL8300)
IL27 (GSE44955/GPL6244)
TNF (GSE18686/GPL6947)
HIV Bal (GSE42925/GPL570)
IL17 (GSE18686/GPL6947)
CD11c high sorted cells (GSE22342/GPL570)
M. tuberculosis (GSE11199/GPL570)
decoy receptor 3 (GSE10856/GPL570)
IL10 (GSE56989/GPL6244)
multinucleated giant cells (GSE29575/GPL6848)
IFNg (GSE49240/GPL10558)
HIF-2alpha RNAi (GSE56989/GPL6244)
concanavalin A (GSE30273/GPL6848)
rosiglitazone, 4 hrs (GSE35967/GPL6947)
alternative M2 activation (GSE35449/GPL6947)
hypoxia (GSE56989/GPL6244)
TNF + PGE2 + P3C, 24 hrs (GSE46903/GPL6947)
Area = 0.13
Area = 0.11
Area = -0.08
Area = 0.08
Area = 0.06
Area = 0.08
Area = -0.06
Area = 0.05
Area = 0.11
Area = 0.05
Area = 0.05
Area = 0.05
Area = -0.04
Area = 0.06
Area = 0.04
Area = 0.14
Area = -0.04
Area = -0.05
Area = -0.07
Area = -0.03
P = 2.91e-07
P = 2.2e-05
P = 5.11e-03
P = 1.41e-02
P = 1.56e-02
P = 7.1e-04
P = 2.48e-02
P = 2.95e-02
P = 1.32e-05
P = 4.42e-02
P = 3.37e-02
P = 7.6e-02
P = 7.73e-02
P = 2.42e-02
P = 1.15e-01
P = 1.81e-07
P = 1.34e-01
P = 4.87e-02
P = 1.02e-02
P = 1.68e-01
-0.4
-0.2
0.0
0.2
0.4
PP-Increased DEGPs
Area = -0.22
Area = -0.19
Area = 0.19
Area = -0.17
Area = -0.11
Area = -0.1
Area = 0.09
Area = -0.09
Area = -0.08
Area = -0.1
Area = -0.08
Area = -0.09
Area = 0.11
Area = -0.07
Area = -0.07
Area = -0.08
Area = 0.07
Area = 0.06
Area = 0.06
Area = 0.09
P = 1.96e-07
P = 1.95e-06
P = 1.13e-04
P = 1.29e-03
P = 1.36e-02
P = 1.64e-02
P = 2.76e-02
P = 2.35e-02
P = 4.16e-02
P = 1.11e-02
P = 6.78e-02
P = 5.74e-02
P = 6.58e-03
P = 9.95e-02
P = 1.2e-01
P = 1.23e-01
P = 6.99e-02
P = 1.45e-01
P = 1.67e-01
P = 3.31e-02
-0.4
-0.2
0.0
0.2
0.4
PP-Decreased DEGPs
(J)

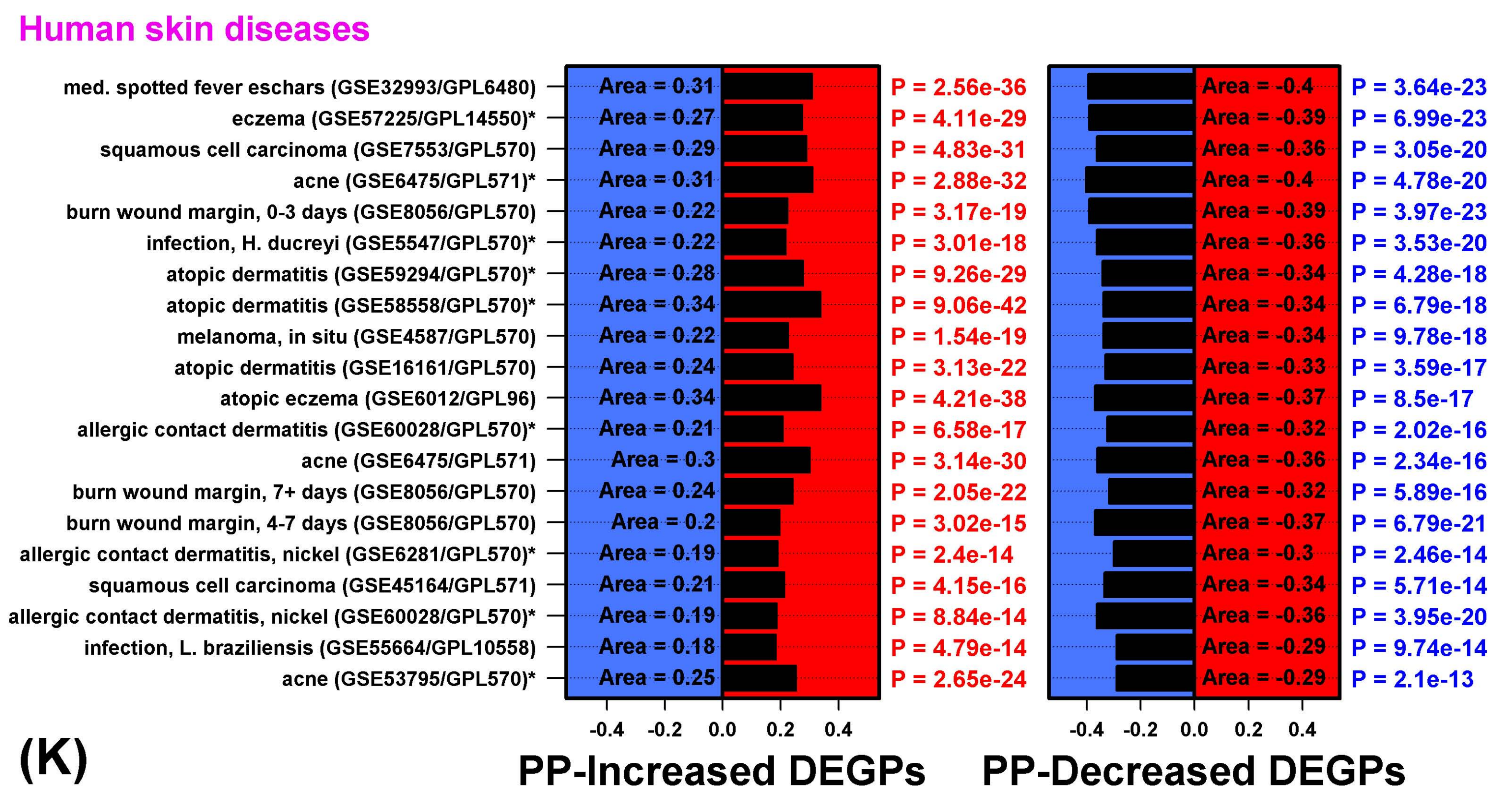

Human skin diseases
med. spotted fever eschars (GSE32993/GPL6480)
eczema (GSE57225/GPL14550)*
squamous cell carcinoma (GSE7553/GPL570)
acne (GSE6475/GPL571)*
burn wound margin, 0-3 days (GSE8056/GPL570)
infection, H. ducreyi (GSE5547/GPL570)*
atopic dermatitis (GSE59294/GPL570)*
atopic dermatitis (GSE58558/GPL570)*
melanoma, in situ (GSE4587/GPL570)
atopic dermatitis (GSE16161/GPL570)
atopic eczema (GSE6012/GPL96)
allergic contact dermatitis (GSE60028/GPL570)*
acne (GSE6475/GPL571)
burn wound margin, 7+ days (GSE8056/GPL570)
burn wound margin, 4-7 days (GSE8056/GPL570)
allergic contact dermatitis, nickel (GSE6281/GPL570)*
squamous cell carcinoma (GSE45164/GPL571)
allergic contact dermatitis, nickel (GSE60028/GPL570)*
infection, L. braziliensis (GSE55664/GPL10558)
acne (GSE53795/GPL570)*
Area = 0.31
Area = 0.27
Area = 0.29
Area = 0.31
Area = 0.22
Area = 0.22
Area = 0.28
Area = 0.34
Area = 0.22
Area = 0.24
Area = 0.34
Area = 0.21
Area = 0.3
Area = 0.24
Area = 0.2
Area = 0.19
Area = 0.21
Area = 0.19
Area = 0.18
Area = 0.25
P = 2.56e-36
P = 4.11e-29
P = 4.83e-31
P = 2.88e-32
P = 3.17e-19
P = 3.01e-18
P = 9.26e-29
P = 9.06e-42
P = 1.54e-19
P = 3.13e-22
P = 4.21e-38
P = 6.58e-17
P = 3.14e-30
P = 2.05e-22
P = 3.02e-15
P = 2.4e-14
P = 4.15e-16
P = 8.84e-14
P = 4.79e-14
P = 2.65e-24
Area = -0.4
Area = -0.39
Area = -0.36
Area = -0.4
Area = -0.39
Area = -0.36
Area = -0.34
Area = -0.34
Area = -0.34
Area = -0.33
Area = -0.37
Area = -0.32
Area = -0.36
Area = -0.32
Area = -0.37
Area = -0.3
Area = -0.34
Area = -0.36
Area = -0.29
Area = -0.29
P = 3.64e-23
P = 6.99e-23
P = 3.05e-20
P = 4.78e-20
P = 3.97e-23
P = 3.53e-20
P = 4.28e-18
P = 6.79e-18
P = 9.78e-18
P = 3.59e-17
P = 8.5e-17
P = 2.02e-16
P = 2.34e-16
P = 5.89e-16
P = 6.79e-21
P = 2.46e-14
P = 5.71e-14
P = 3.95e-20
P = 9.74e-14
P = 2.1e-13
-0.4 -0.2 0.0 0.2 0.4
-0.4 -0.2 0.0 0.2 0.4
(K)
PP-Increased DEGPs
PP-Decreased DEGPs
